# The genomes of *Dahlia pinnata, Cosmos bipinnatus*, and *Bidens alba* in tribe Coreopsideae provide insights into polyploid evolution and inulin biosynthesis

**DOI:** 10.1093/gigascience/giae032

**Published:** 2024-06-13

**Authors:** Hengchao Wang, Dong Xu, Fan Jiang, Sen Wang, Anqi Wang, Hangwei Liu, Lihong Lei, Wanqiang Qian, Wei Fan

**Affiliations:** Guangdong Laboratory for Lingnan Modern Agriculture (Shenzhen Branch), Genome Analysis Laboratory of the Ministry of Agriculture and Rural Affairs, Agricultural Genomics Institute at Shenzhen, Chinese Academy of Agricultural Sciences, Shenzhen, Guangdong 518120, China; Guangdong Laboratory for Lingnan Modern Agriculture (Shenzhen Branch), Genome Analysis Laboratory of the Ministry of Agriculture and Rural Affairs, Agricultural Genomics Institute at Shenzhen, Chinese Academy of Agricultural Sciences, Shenzhen, Guangdong 518120, China; Guangdong Laboratory for Lingnan Modern Agriculture (Shenzhen Branch), Genome Analysis Laboratory of the Ministry of Agriculture and Rural Affairs, Agricultural Genomics Institute at Shenzhen, Chinese Academy of Agricultural Sciences, Shenzhen, Guangdong 518120, China; Guangdong Laboratory for Lingnan Modern Agriculture (Shenzhen Branch), Genome Analysis Laboratory of the Ministry of Agriculture and Rural Affairs, Agricultural Genomics Institute at Shenzhen, Chinese Academy of Agricultural Sciences, Shenzhen, Guangdong 518120, China; Guangdong Laboratory for Lingnan Modern Agriculture (Shenzhen Branch), Genome Analysis Laboratory of the Ministry of Agriculture and Rural Affairs, Agricultural Genomics Institute at Shenzhen, Chinese Academy of Agricultural Sciences, Shenzhen, Guangdong 518120, China; Guangdong Laboratory for Lingnan Modern Agriculture (Shenzhen Branch), Genome Analysis Laboratory of the Ministry of Agriculture and Rural Affairs, Agricultural Genomics Institute at Shenzhen, Chinese Academy of Agricultural Sciences, Shenzhen, Guangdong 518120, China; Guangdong Laboratory for Lingnan Modern Agriculture (Shenzhen Branch), Genome Analysis Laboratory of the Ministry of Agriculture and Rural Affairs, Agricultural Genomics Institute at Shenzhen, Chinese Academy of Agricultural Sciences, Shenzhen, Guangdong 518120, China; Guangdong Laboratory for Lingnan Modern Agriculture (Shenzhen Branch), Genome Analysis Laboratory of the Ministry of Agriculture and Rural Affairs, Agricultural Genomics Institute at Shenzhen, Chinese Academy of Agricultural Sciences, Shenzhen, Guangdong 518120, China; Guangdong Laboratory for Lingnan Modern Agriculture (Shenzhen Branch), Genome Analysis Laboratory of the Ministry of Agriculture and Rural Affairs, Agricultural Genomics Institute at Shenzhen, Chinese Academy of Agricultural Sciences, Shenzhen, Guangdong 518120, China

**Keywords:** Coreopsideae, *Dahlia pinnata*, *Cosmos bipinnatus*, *Bidens alba*, whole genome duplication, inulin

## Abstract

**Background:**

The Coreopsideae tribe, a subset of the Asteraceae family, encompasses economically vital genera like *Dahlia, Cosmos*, and *Bidens*, which are widely employed in medicine, horticulture, ecology, and food applications. Nevertheless, the lack of reference genomes hinders evolutionary and biological investigations in this tribe.

**Results:**

Here, we present 3 haplotype-resolved chromosome-level reference genomes of the tribe Coreopsideae, including 2 popular flowering plants (*Dahlia pinnata* and *Cosmos bipinnatus*) and 1 invasive weed plant (*Bidens alba*), with assembled genome sizes 3.93 G, 1.02 G, and 1.87 G, respectively. We found that *Gypsy* transposable elements contribute mostly to the larger genome size of *D. pinnata*, and multiple chromosome rearrangements have occurred in tribe Coreopsideae. Besides the shared whole-genome duplication (WGD-2) in the Heliantheae alliance, our analyses showed that *D. pinnata* and *B. alba* each underwent an independent recent WGD-3 event: in *D. pinnata*, it is more likely to be a self-WGD, while in *B. alba*, it is from the hybridization of 2 ancestor species. Further, we identified key genes in the inulin metabolic pathway and found that the pseudogenization of *1-FEH1* and *1-FEH2* genes in *D. pinnata* and the deletion of 3 key residues of 1-FFT proteins in *C. bipinnatus* and *B. alba* may probably explain why *D. pinnata* produces much more inulin than the other 2 plants.

**Conclusions:**

Collectively, the genomic resources for the Coreopsideae tribe will promote phylogenomics in Asteraceae plants, facilitate ornamental molecular breeding improvements and inulin production, and help prevent invasive weeds.

## Introduction

The tribe Coreopsideae in Asteraceae consists of several economically important species in genera *Dahlia, Cosmos*, and *Bidens. Dahlia pinnata* (NCBI:txid101596) is a popular tuberous-rooted ornamental flower crop, widely grown for cut flowers and potted flowers. It is recognized as the national flower of Mexico, and more than 65,500 cultivars of *D. pinnata* have been bred worldwide [1]. *D. pinnata* also contains a lot of inulin and many nutritional compounds in tuberous roots, which can improve a healthy diet [2]. *Cosmos bipinnatus*, commonly known as the garden cosmos or lace cosmos, is popular not only as a garden or a bedding plant but also as cut flowers [3]. *Bidens alba* (NCBI:txid51260) is a worldwide invasive weed that has been listed among the first class of most malignant invasion in the Invasive Alien Species of China [4]. Additionally, both *C. bipinnatus* and *B. alba* have been widely used as traditional medicines, due to their anti-inflammatory, antioxidative, and antibacterial activities [5–8].

Comprising about 550 species, Coreopsideae is a tribe of flowering plants within the Heliantheae alliance of the Asteraceae family [9]. Coreopsideae consists of a diverse group of plants primarily found in the Americas, with the highest diversity in North America [10]. It was once thought of as the subtribe Coreopsidinae in the tribe Heliantheae according to the morphology taxonomy [11]. Similar to other Asteraceae sublineages, many species in this tribe are rich in inulin, a compound widely utilized in food, health, and cosmetic industries as sweetener, dietary fiber, viscosity modifier, fat replacer, and prebiotics [12–14]. In addition, most plants in the Asteraceae employed inulin as the primary reserve carbohydrates, instead of starches, which may contribute to strong capability of environmental adaptation for these plants [15–18]. The tubers of *D. pinnata*, chicory, and Jerusalem artichoke are currently the main sources of inulin for industrial extraction [19].

Recently, chromosome-scale reference genomes have been published for several economically important species in the Heliantheae alliance, including *Helianthus annuus* [20], *Ambrosia artemisiifolia* [21], *Stevia rebaudiana* [22], *Mikania micrantha* [23], and *Smallanthus sonchifolius* [15]. However, until now, for the tribe Coreopsideae, only the organelle genomes of *D. pinnata, C. bipinnatus*, and *B. alba*, as well as a highly fragmented contig-level assembly of the hexaploid *Bidens hawaiensis*, were available [24–29]. In this work, we present the chromosome-scale haplotype-resolved genomes of *D. pinnata, C. bipinnatus*, and *B. alba*, to explore the evolution of polyploidization and mechanism under inulin metabolism.

## Results

### Chromosome-scale haplotype-resolved assemblies of *D. pinnata, C. bipinnatus*, and *B. alba*

According to previous studies, *D. pinnata* has a 2n = 4x = 64 karyotype [30–32], and *C. bipinnatus* has a 2n = 2x = 24 karyotype [30, 33]. However, in the genus *Bidens*, many plants have a similar morphology, and there are tetraploid and hexaploid populations [26, 34]. Previous reports showed that *B. alba* has a 2n = 4x = 48 karyotype [26, 35], and our karyotype analysis of *B. alba* by fluorescence in situ hybridization (FISH) delivered the same result (Supplementary Fig. S1).

**Figure 1:**
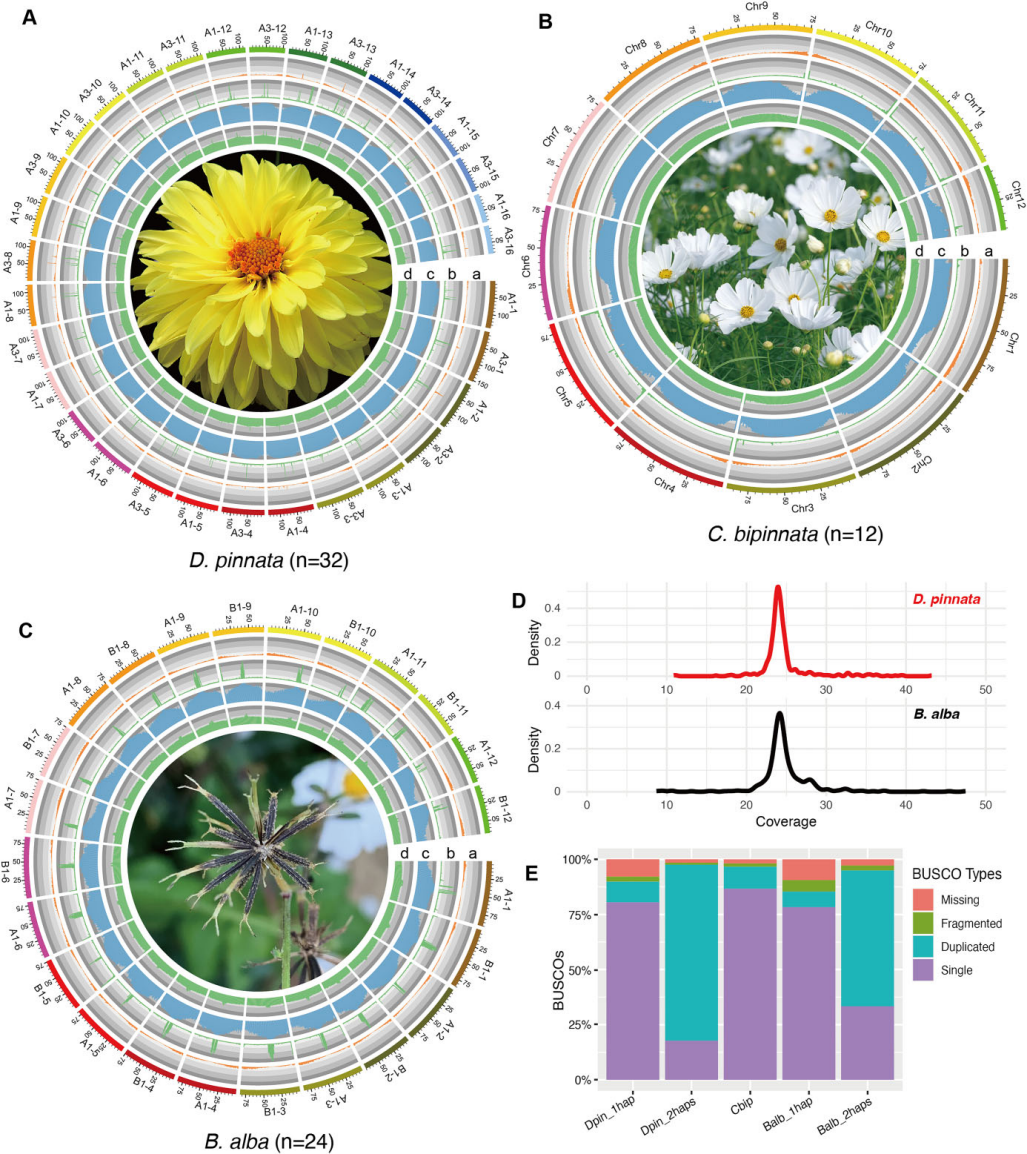
Genomic features of *D. pinnata, C. bipinnata*, and *B. alba*. (A–C) Circos plots of 3 plants in tribe Coreopsideae, including *D. pinnata* (A), *C. bipinnata* (B), and *B. alba* (C). Each circos plot has 4 tracks: a, gene density; b, tandem repeat density; c, transposable element (TE) density; d, GC percentage. Feature density and GC percentage were calculated with a 1-Mb window size. A picture with representative traits is in the center of each circos plot. Note that the reference genomes for each species were shown. *C. bipinnata* is diploid, so the haploid genome was used as reference genome. In contrast, *D. pinnata* and *B. alba* are tetraploids; their haploid genomes containing 2 subgenomes were used as their reference genomes. (D) The distribution of HiFi reads coverage on the contigs (>1 Mb) of tetraploid *D. pinnata* and *B. alba*. Each plot has a major peak, suggesting that almost no homologous contigs were collapsed. (E) The BUSCO completeness of gene sets for 1 haplotype of *D. pinnata* (Dpin_1hap), 2 haplotypes of *D. pinnata* (Dpin_2haps: A1 and A3), *C. bipinnata* (Cbip), 1 haplotype of *B. alba* (Balb_1hap), and 2 haplotypes of *B. alba* (Balb_2haps: A1 and B1).

To build high-quality reference genomes, we sequenced *D. pinnata, C. bipinnatus*, and *B. alba* with Pacific Biosciences (PacBio) High-Fidelity (HiFi) and long-range Illumina Hi-C technologies (Supplementary Table S1). Using hifiasm [36] with input of 192.5 Gb (48×), 78.0 Gb (72×), and 93.2 Gb (48×) HiFi data as well as 368.0 Gb (92×), 113.7 Gb (105×), and 163.1 Gb (85×) Hi-C data, we obtained contig assembly sizes 7.86 Gb, 1.02 Gb, and 3.75 Gb with contig N50 sizes 29.28 Mb, 52.82 Mb, and 15.05 Mb for *D. pinnata, C. bipinnatus*, and *B. alba*, respectively (Table 1). Because of the low heterozygosity rate, the assembly size of *C. bipinnatus* (1.02 Gb) is similar to the estimated haploid genome size (1.08 Gb) by *k*-mer analysis (Supplementary Fig. S2). However, due to the high heterozygosity rates and tetraploid karyotype characters, the assembly sizes of *D. pinnata* (7.86 Gb) and *B. alba* (3.75 Gb) are about 2 times the estimated haploid genome sizes (3.98 Gb of *D. pinnata* and 1.93 Gb of *B. alba*) by *k*-mer analysis (Supplementary Fig. S2), implying that we acquired both allelic genomes of the 2 tetraploid plants. Additionally, we downloaded the reference organelle genomes of closely related plants, aligned them with the contig assemblies, and filtered 4.07 Mb, 0.54 Mb, and 8.74 Mb organelle sequences for *D. pinnata, C. bipinnatus*, and *B. alba*, respectively (Supplementary Tables S2–S4). Finally, we used YaHS [37] and Juicebox [38] to anchor the contigs onto chromosome-scale scaffolds (Supplementary Tables S5–S7), resulting in 95.5%, 93.7%, and 95.2% anchoring rates, with scaffold N50 sizes 118.5 Mb, 79.4 Mb, and 75.1 Mb for *D. pinnata, C. bipinnatus*, and *B. alba*, respectively (Fig. 1A–C, Table 1, and Supplementary Table S8). For the 2 tetraploid genomes *D. pinnata* and *B. alba*, strong Hi-C signals formed along with the diagonals within a chromosome and between chromosomes of the same homologous chromosomes group (Supplementary Fig. S3). Moreover, 90 (70.3%), 14 (58.3%), and 55 (57.3%) candidate telomeres were found on the chromosomes of *D. pinnata, C. bipinnatus*, and *B. alba*, respectively (Supplementary Tables S9–S11).

**Table 1:** Statistics of genome assembly and annotation

	*D. pinnata* (4×)	*D. pinnata* (2×)	*C. bipinnatus*	*B. alba* (4×)	*B. alba* (2×)
Genome assembly					
Estimate genome size (Gb)	7.96	3.98	1.08	3.86	1.93
Total assembly size (bp)	7,857,309,314	3,928,656,657	1,015,576,390	3,749,125,044	1,874,562,522
Number of chromosomes	64	32	12	48	24
GC percentage	37.8	37.8	36.8	36.6	36.6
Contig N50 (bp)	29,279,538	29,279,538	52,817,601	15,049,174	15,049,174
Scaffold N50 (bp)	118,510,872	118,510,872	79,416,621	75,126,418	75,126,418
% of sequences anchored to chromosomes	95.5	95.5	93.7	95.2	95.2
% of telomeres assembled	70.3	70.3	58.3	57.3	57.3
BUSCO completeness assembly, %	99.1	98.8	97.4	99.6	98.4
Genome annotation					
Length of tandem repeats (bp)	657,362,351	328,681,176	69,395,159	440,213,780	220,106,890
Length of TE sequences (bp)	6,887,204,637	3,443,602,318	816,414,084	2,909,120,353	1,454,560,176
Number of tRNA genes	4,717	2,358	1,474	4,998	2,499
Number of rRNA genes	51,314	25,657	10,828	21,488	10,744
Number of protein-coding gene models	181,915	90,958	46,076	165,431	82,716
Total CDS size (bp)	224,643,068	112,321,534	58,778,603	181,196,996	90,598,498
BUSCO completeness of gene set, %	98.9	97.6	96.7	98.4	95.0

*Note*: Two haplotypes of *D. pinnata* (A1 and A3) and *B. alba* (A1 and B1) were assessed by BUSCO.

After assembly, we mapped HiFi data to the genomes of 2 tetraploid plants (*D. pinnata* and *B. alba*) and checked the reads coverage distribution. The results showed that most contigs of both plants have approximately 24× mean reads coverage (Fig. 1D), which is half of the estimated reads coverage for the haploid genome, indicating that we successfully assembled haplotype-resolved genomes. Besides, the BUSCO complete rates of the genomes are 99.1%, 97.4%, and 99.6% for *D. pinnata, C. bipinnatus*, and *B. alba*, respectively, which are higher than or comparable to those of related published species (Supplementary Table S12).

Integrating evidence from full-length transcriptome (isoform sequencing [Iso-seq]), short-reads transcriptome (RNA sequencing [RNA-seq]), and homologous proteins, Augustus [39] predicted 181,915, 46,076, and 165,431 protein-coding genes for *D. pinnata, C. bipinnatus*, and *B. alba*, respectively (Supplementary Fig. S4, Supplementary Tables S13–S17). The BUSCO complete rates of the gene sets were 98.9%, 96.7%, and 98.4% for *D. pinnata, C. bipinnatus*, and *B. alba*, respectively (Supplementary Table S18). For the 2 tetraploid plants, we also compared the BUSCO completeness of genes from 1 haplotype (1 subgenome) and 2 haplotypes. The results showed that the complete and duplicated rates of 2 haplotypes were both higher than that of 1 haplotype (Fig. 1E). For gene functional annotation, 173,686 (95.48%) of *D. pinnata*, 44,074 (95.66%) of *C. bipinnatus*, and 151,025 (91.29%) of *B. alba* genes were annotated by at least one of NR, KEGG, Uniprot (Swiss-Prot), and InterPro databases (Supplementary Table S19). Based on the gene sets, we predicted transcription factors (TFs) by PlantTFDB [40], and the most abundant TF families were bHLH, ERF, MYB, and C2H2 (Supplementary Table S20). Moreover, we also predicted transfer RNAs (tRNAs), ribosomal RNAs (rRNAs), and noncoding RNAs (ncRNAs) (Supplementary Tables S21 and S22).

### Recent expansion of *Gypsy* long terminal repeats enlarges the genome of *D. pinnata*

**Figure 2:**
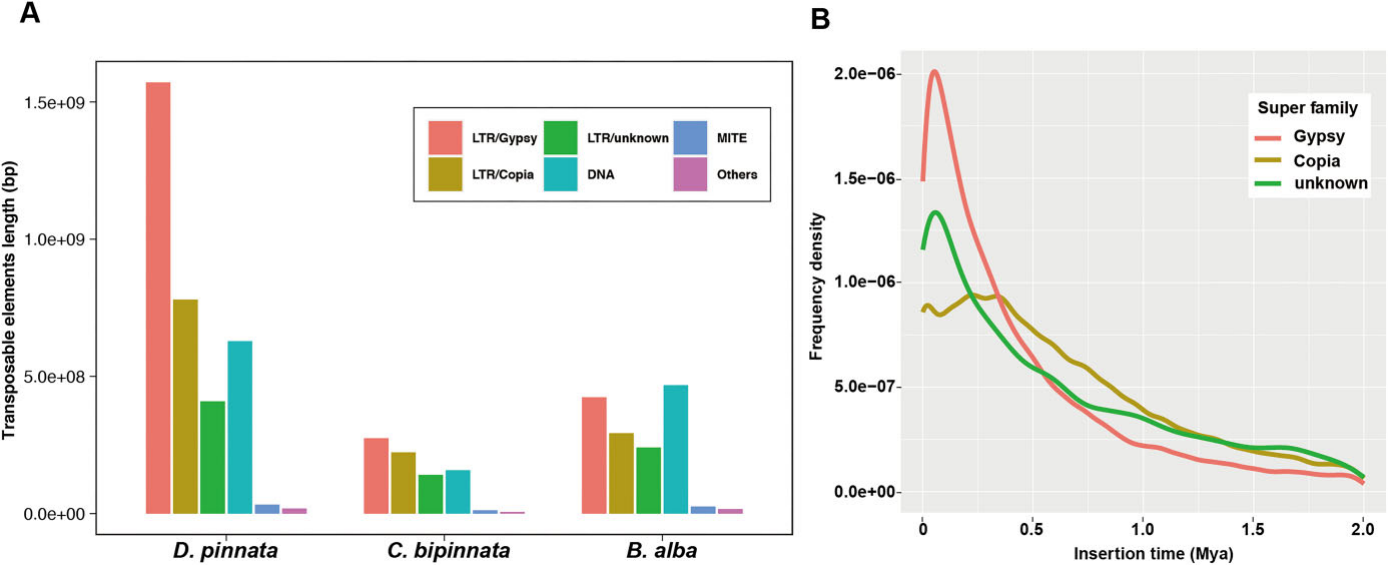
Distributions and variations of transposable elements. (A) Histogram of different types of transposable elements (TEs) for *D. pinnata, C. bipinnata*, and *B. alba*. (B) The estimated insertion time of long terminal repeats (LTRs) in *D. pinnata*. The intact LTRs were annotated by EDTA, and pairwise distances from the LTRs were estimated by APE with the K80 model. The sequence divergence (*d*) of long terminal repeats of each LTR and the mutation rate of sunflower (*r* = 1×10^−8^) were used to estimate the times with the equation *T = d/2r*.

In comparison to *C. bipinnatus* and *B. alba, D. pinnata* has a much bigger genome size. Apart from the effect of whole-genome duplications, the genome sizes of 1 haplotype were about 1.99 Gb, 1.08 Gb, and 0.97 Gb for *D. pinnata, C. bipinnatus*, and *B. alba*, respectively. Notably, 1.72 Gb (86%) of *D. pinnata*, 0.82 Gb (76%) of *C. bipinnatus*, and 0.73 Gb (75%) of *B. alba* were TEs (Table 1), implying that TE expansion is the major force that enlarges the genome of *D. pinnata*. Furthermore, we compared the abundances of different TE classes and found that long terminal repeats (LTRs) were the most abundant TEs for all 3 plants (Supplementary Table S23). Among the LTRs, the *Gypsy* TEs were most abundant (Fig. 2A). In addition to the quantities, we also estimated the insertion time of different subclasses of LTRs. These LTR TEs were mainly expanded within the past 1 million years (Fig. 2B and Supplementary Fig. S5), which is similar to that of sunflower [20]. Considering that *Gypsy* LTRs were the dominant LTRs and the recent insertion peak of *Gypsy* LTRs was much higher than that of the *Copia* and other LTRs, we speculate that the recent expansion of *Gypsy* LTRs contributed mostly to the larger genome size of *D. pinnata*.

### Multiple chromosome rearrangements experienced in tribe Coreopsideae

**Figure 3:**
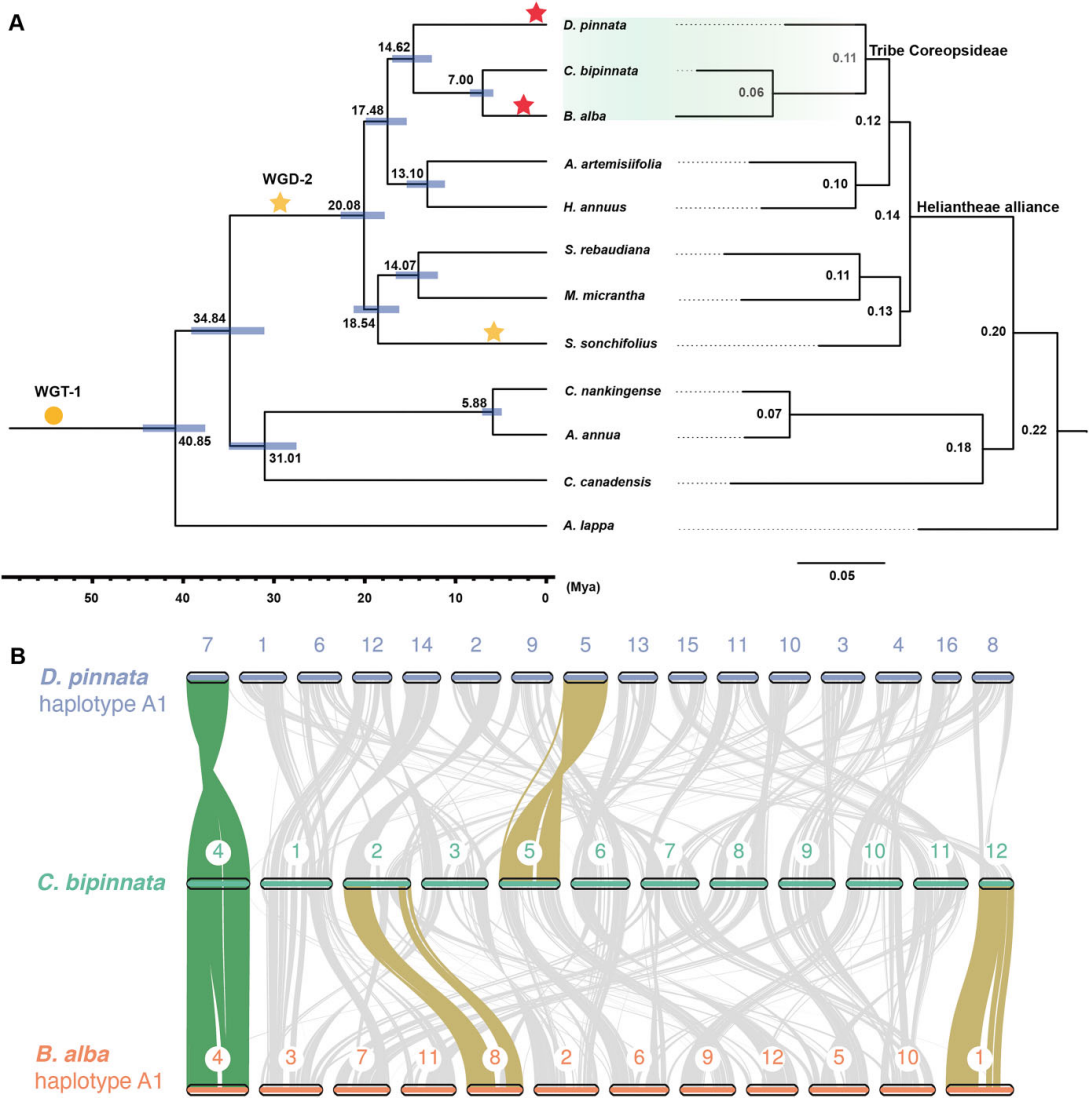
Evolution history and chromosome rearrangements in tribe Coreopsideae. (A) Phylogenetic trees showing divergence times (left) and substitution rates (right) for the 3 representative plants in tribe Coreopsideae and closely related species. The blue horizontal bars indicate 95% confidence intervals of the inner nodes. Circle stands for whole-genome triplication (WGT), and stars represent whole-genome duplications (WGDs). Shapes with a yellow background are whole-genome polyploidization events reported previously, while shapes with a red background are those reported in this study. The clade containing the 3 plants in the tribe Coreopsideae is highlighted with a green background. (B) Macro-synteny plot of 1 haplotype of *D. pinnata* and *C. bipinnatus*, and 1 haplotype of *B. alba*. The chromosomes showing 1:1:1 relationships among all 3 species are highlighted in green, while the chromosomes showing 1:1 relationships among only 2 species are highlighted in yellow. The others showing multiple chromosome rearrangements are colored in gray.

To resolve the phylogenetic relationships of plants in the tribe Coreopsideae, we performed evolutionary analyses using the gene sets of 12 Asteraceae plants, including *D. pinnata, C. bipinnata, B. alba, A. artemisiifolia* [21], *H. annuus* [20], *S. rebaudiana* [22], *M. micrantha* [23], *S. sonchifolius* [15], *Chrysanthemum nankingense* [41], *Artemisia annua* [42], *Conyza canadensis* [43], and *Arctium lappa* [15], with *Vitis vinifera* as the outgroup (Supplementary Tables S17 and S24). In total, OrthoFinder assigned 681,319 genes (93.8% of the total) to 40,991 orthogroups [44], and there were 9,121 orthogroups with all species present (Supplementary Table S25). Using 354 orthogroups with a minimum of 46.2% of species having single-copy genes, OrthoFinder called STAG to build a species tree and STRIDE to root this tree (Supplementary Fig. S6). On the other hand, we built a phylogenetic tree using RAxML-NG with the data of 274 single-copy genes for each species (Fig. 3A). The 2 species trees had the same topology and very similar sequence evolution rates (branch lengths), demonstrating that the species tree is credible.

The relationships of the 3 genera *Dahlia, Cosmos*, and *Bidens* in the tribe Coreopsideae have been in controversy. Previous phylotranscriptomic analyses supported that the genera *Dahlia* and *Cosmos* are sisters, and the genus *Bidens* is a sister of the last common ancestor of the genera *Dahlia* and *Cosmos* [45]. However, other studies by internal transcribed spacer (ITS) data [46] and plastid DNA sequences [10] supported that the genus *Cosmos* is closer to the genus *Bidens*, and the genus *Dahlia* is sister with the last common ancestor of the genera *Cosmos* and *Bidens*. Our study with the genome-wide data showed that *Cosmos* and *Bidens* are closer than *Dahlia* (Fig. 3A), which is consistent with the studies from ITS and plastid data. Using the molecular clock dating method, we estimated the divergence times among these plants (Fig. 3A). In the Heliantheae alliance, the tribe Coreopsideae and the tribe Heliantheae separated 17.48 million years ago (Mya). *D. pinnata* diverged from the last common ancestor of *C. bipinnata* and *B. alba* 14.62 Mya, while *C. bipinnata* and *B. alba* diverged from each other 7.00 Mya. Notably, *B. alba* mutated faster than *C. bipinnatus, D. pinnata*, and other related plants in Asteraceae. As a notorious invasive weed, the fast mutation rate may contribute to the strong invasive capabilities of *B. alba*.

With the chromosome-scale haplotype-resolved assemblies of *D. pinnata, C. bipinnata*, and *B. alba*, we studied chromosome conservation and rearrangement among these plants (Fig. 3B). From the macro-synteny plot, we showed that only 1 chromosome group, including the Chr 7 of *D. pinnata*, the Chr 4 of *C. bipinnata*, and the Chr 4 of *B. alba*, preserved chromosome-scale collinearity among the 3 species. Meanwhile, there were 3 chromosome pairs that preserved chromosome-scale collinearity between 2 species, including Chr 5 of *D. pinnata* and Chr 5 of *C. bipinnata*, Chr 2 of *C. bipinnata* and Chr 8 of *B. alba*, and Chr 12 of *C. bipinnata* and Chr 1 of *B. alba*. Further, the other chromosomes of the 3 plants experienced chromosome fusion and fission events after the last common ancestor of the tribe Coreopsideae.

### Genomic evidence supports *D. pinnata* to be autotetraploid

**Figure 4:**
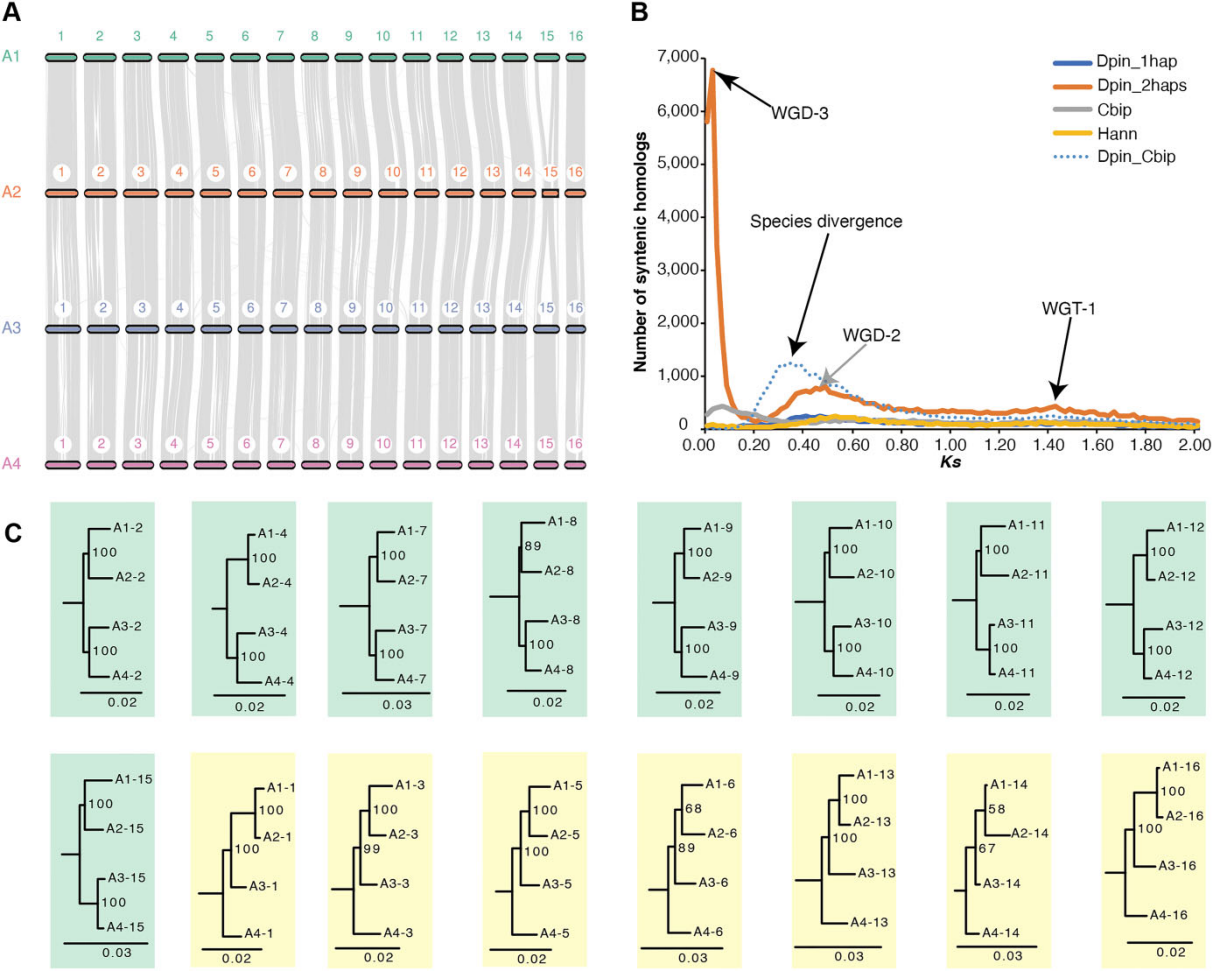
Evolutionary analyses of the tetraploid *D. pinnata* genome. (A) Macro-synteny of 4 haplotypes in *D. pinnata*. Each haplotype contains 16 chromosomes. (B) Synonymous substitution rate per site (*Ks*) distribution for 1 haplotype of *D. pinnata* (Dpin_1hap), 2 haplotypes of *D. pinnata* (Dpin_2haps: A1 and A3), *C. bipinnata* (Cbip), *H. annuus* (Hann), and between 1 haplotype of *D. pinnata* and *C. bipinnata* (Dpin_Cbip). Speciation and whole-genome polyploidization events are pointed out by arrows. (C) Phylogenetic trees for each of the 16 homologous chromosome groups. In total, 9 chromosome groups with ((A1, A2), (A3, A4)) topology are highlighted with a green background, while 7 chromosome groups with (((A1, A2), A3), A4) topology are highlighted with a yellow background.

The ploidy of *D. pinnata* has been in controversy. Some scholars proposed *D. pinnata* is an allooctoploid species without evidence [47] and based on chromosome pairing patterns [48]. Using simple-sequence repeat and amplified fragment length polymorphism data, Stephan and colleagues indicated that *D. pinnata* has an auto-octoploid genome [31]. However, another study suggested *D. pinnata* to be a tetraploid species by comparing chromosome numbers of the close relatives [49]. Based on the chromosome-scale haplotype-resolved assembly, we observed strong head-to-tail collinearity among the 4 haplotypes in each of the 16 homologous chromosome groups, without any translocation between any 2 chromosome groups (Fig. 4A and Supplementary Fig. S7). These results implied that *D. pinnata* has a tetraploid genome architecture, which is derived from a recent whole-genome duplication.

To determine the time of the recent whole-genome duplication in *D. pinnata*, we analyzed the distribution of the synonymous substitution rate (*Ks*) of homologous gene pairs for 1 haplotype (subgenome) and 2 haplotypes of *D. pinnata, C. bipinnata*, and *H. annuus*. In comparison, we also analyzed the distribution of *Ks* between 1 haplotype of *D. pinnata* and *C. bipinnata* (Fig. 4B). For *D. pinnata*, the *Ks* peaks at about 1.4 and 0.5 correspond to WGT-1 and WGD-2, respectively, which are shared in the lineage of the Heliantheae alliance [15]. Besides, *D. pinnata* has a specific *Ks* peak at ∼0.03, which is responsible for the recent whole-genome duplication (WGD-3). Given that the *Ks* peak corresponding to the divergence between *D. pinnata* and *C. bipinnatus* is ∼0.35, and this divergence occurred ∼14.62 Mya (Fig. 3A), we estimated that the WGD-3 event of *D. pinnata* occurred ∼1.25 Mya. Thus, the time of the WGD-3 event was very close to the current, which explains the strong macro-synteny among chromosome haplotypes.

To further study the polyploid history of *D. pinnata*, we constructed the phylogenetic tree of each homologous chromosome group, taking each chromosome as a virtual species by the single-copy gene concatenation method. In total, there were 9 homologous groups with ((A1, A2), (A3, A4)) topology and 7 homologous groups with (((A1, A2), A3), A4) topology (Fig. 4C). Considering that the WGD-3 of *D. pinnata* occurred just ∼1.25 Mya, and *D. pinnata* has the lowest mutation rate among the 3 plants of the tribe Coreopsideae (Fig. 3A), the sequence difference between the duplicated chromosome pairs derived from WGD-3 is still not large enough to distinguish from the sequence difference from the 2 allelic chromosomes that have high heterozygosity. Additionally, we attempted to phase subgenomes of *D. pinnata* based on subgenome-specific *k*-mers, resulting in very few subgenome-specific *k*-mers, and thus we failed to phase the subgenomes due to the very recent LTR insertion (Supplementary Fig. S8). Therefore, it is difficult to get the ((A1, A2), (A3, A4)) topology for all the homologous chromosome groups and impossible to clearly distinguish the subgenomes with allelic genomes from current data. Taking together all the evidence, we concluded that the ploidy of *D. pinnata* is more likely to be an autotetraploid, which originated from WGD-3 of a single ancestor species.

### Genomic evidence reveals *B. alba* as an allotetraploid

**Figure 5:**
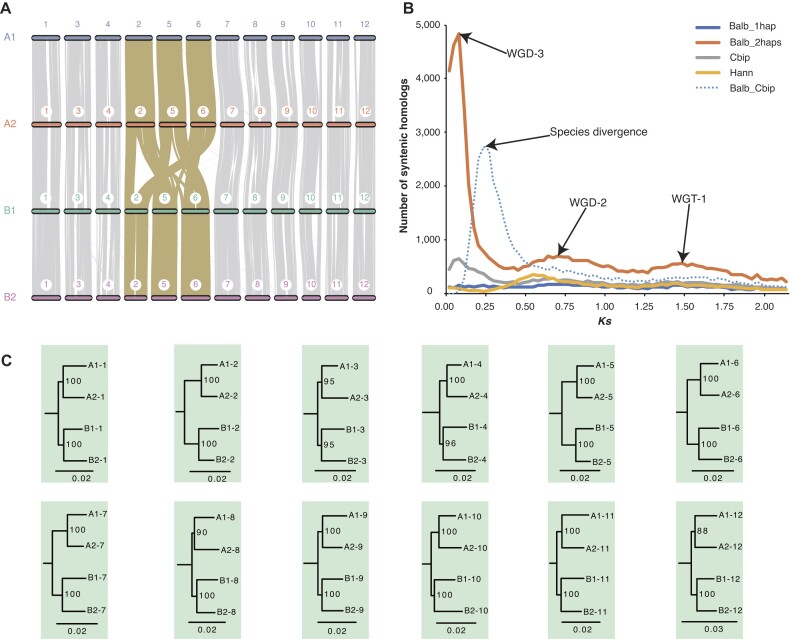
Evolutionary analyses of the tetraploid *B. alba* genome. (A) Macro-synteny of 4 haplotypes in *B. alba*. Each haplotype contains 12 chromosomes. (B) Synonymous substitution rate per site (*Ks*) distribution for 1 haplotype of *B. alba* (Balb_1hap), 2 haplotypes of *B. alba* (Balb_2haps: A1 and B1), *C. bipinnata* (Cbip), *H. annuus* (Hann), and between 1 haplotype of *B. alba* and *C. bipinnata* (Balb_Cbip). Speciation and whole-genome polyploidization events are pointed out by arrows. (C) Phylogenetic trees for each of the 12 homologous chromosome groups. All 12 chromosome groups held ((A1, A2), (B1, B2)) topology and are highlighted with a green background.

To study the evolution history of chromosomes in *B. alba*, we showed the macro-synteny for the 12 groups of homologous chromosomes (Fig. 5A and Supplementary Fig. S9). Among the 12 groups, 9 groups (1, 3, 4, 7, 8, 9, 10, 11, and 12) demonstrated holistic chromosome collinearity for the 4 haplotypes, which was similar to that of *D. pinnata*. In the remaining 3 groups, holistic macro-synteny exists only in 2 pairs of haplotypes but not among all 4 haplotypes, and large fragment translocations occurred among these 3 homologous chromosome groups. These results suggested that *B. alba* has a tetraploid genome architecture, which is derived from a whole-genome duplication, possibly by the hybridization of 2 ancestor species.

To estimate the time of hybridization event of *B. alba*, we analyzed the distribution of synonymous substitution rate (*Ks*) of homologous gene pairs for 1 haplotype and 2 haplotypes of *B. alba, C. bipinnata*, and *H. annuus* and between 1 haplotype of *B. alba* and *C. bipinnata* (Fig. 5B). Since the mutation rate of *B. alba* is faster than that of the other plants analyzed (Fig. 3A), the *Ks* peaks for WGD-2 and WGT-1 were both right-shifted compared with that of *H. annuus*. In addition, *B. alba* has a specific *Ks* peak at ∼0.08, which is caused by the recent whole-genome duplication (WGD-3), that is, the hybridization event. Considering that the *Ks* peak corresponding to the divergence between *B. alba* and *C. bipinnatus* is ∼0.23, and the divergence of these 2 species occurred at ∼7.00 Mya (Fig. 3A), the time of the WGD-3 of *B. alba* was estimated to be ∼2.43 Mya. Note that the real time for the hybridization event may be a little later than 2.43 Mya, because the divergence time estimation used both the accumulated mutations since the hybridization event and the original sequence divergence of the 2 ancestor species.

Using the single-copy gene concatenation method, we constructed the phylogenetic trees for the homologous chromosome groups, taking each chromosome as a virtual species. For all the 12 homologous groups, the trees showed the same topology ((A1, A2),(B1, B2)) (Fig. 5C), indicating that the 4 chromosome haplotypes could be separated into 2 pairs, with each representing a subgenome. On the other hand, we also attempted to phase subgenomes of *B. alba* based on subgenome-specific *k*-mers, resulting in that there were very few subgenome-specific *k*-mers and thus we failed to phase the subgenomes, due to the very recent LTR insertion (Supplementary Fig. S10). Taking together both the macro-synteny and the phylogenetic topology evidence, we concluded that the ploidy of *B. alba* is more likely to be an allotetraploid, which originated from WGD-3 by the hybridization of 2 ancestor species.

### Pseudogenization in 1 of the 2 copies of 1-FEH1 and 1-FEH2 genes in *D. pinnata*

The tubers of *D. pinnata* encompass large quantities of inulin and have been used as one of the main sources of industry inulin production [12, 19]. There are 4 key genes involved in the metabolism of inulin. The enzyme sucrose/sucrose 1-fructosyl transferase (1-SST) catalyzes 2 sucrose molecules to produce 1-kestose and glucose. Then, the enzyme fructan/fructan 1-fructosyl transferase (1-FFT) further elongates linear inulin-type fructans with β(2,1) fructosyl units, taking 1-kestose as the preferential donor. Breakdown of inulin is performed by the action of fructan 1-exohydrolase (1-FEH1 and 1-FEH2), which hydrolyzes terminal fructoses [50]. In addition to these functional genes, previous studies also reported 3 transcription factors in chicory. MYB3 and MYB5 can enhance promoter activities of *1-FEH* genes in response to abiotic stress and hormonal cues, while MYB17 activates promoters of both synthesis (*1-SST* and *1-FFT*) and degradation (*1-FEH*) genes under stress [51, 52]. However, the key genes of *D. pinnata* for inulin biosynthesis and breakdown still lack investigation.

**Figure 6:**
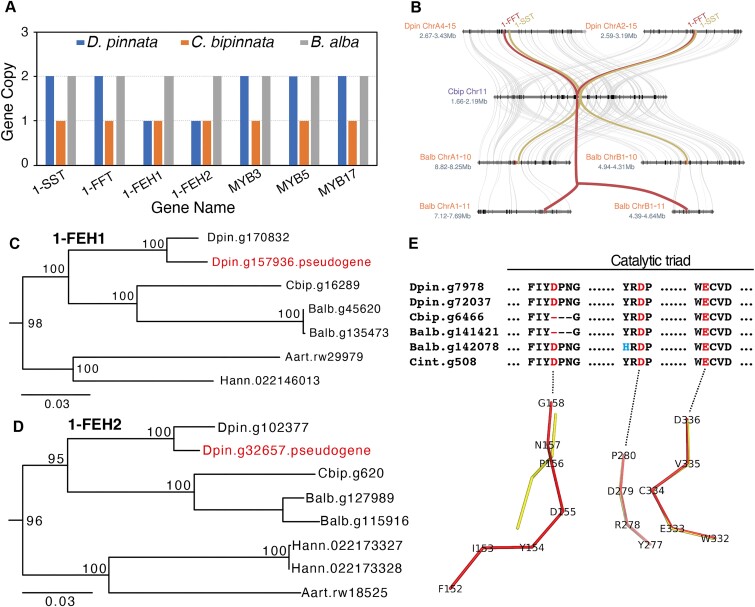
Genes for inulin metabolism and regulation. (A) Copy number for the fructan-active enzyme (FAZY) genes 1-SST, 1-FFT, 1-FEH1, and 1-FEH2 and transcription factor genes MYB17, MYB3, and MYB5 in *D. pinnata, C. bipinnatus*, and *B. alba*. (B) Micro-synteny of 1-FFT and 1-SST genes in *D. pinnata, C. bipinnatus*, and *B. alba*. The tandem duplicated pseudogenes of 1-FFT of *B. alba* are in red. (C) Phylogenetic tree of 1-FEH1 genes in *D. pinnata* (Dpin), *C. bipinnatus* (Cbip), *B. alba* (Balb), *A. artemisiifolia* (Aart), and *H. annuus* (Hann). (D) Phylogenetic tree of 1-FEH2 genes. (E) Multiple sequence alignment of catalytic triads for the 1-FFT proteins in *D. pinnata, C. bipinnatus, B. alba*, and *Cichorium intybus* (Up). Three conserved residues involved in the reaction are highlighted in red, and the 1 residue mutated only in 1 of 2 1-FFT genes from *B. alba* is highlighted in cyan. The ribbon views of the catalytic triads for the protein structures of 1-FFTs of *D. pinnata* (Dpin.g7978, red) and *C. bipinnatus* (Cbip.g6466, yellow) (bottom). The structures were aligned together, and the loss of 3 residues in *C. bipinnatus* severely impacted its protein structures.

Based on homology alignment to known genes, we identified the key genes of functional enzymes and transcription factors for *D. pinnata, C. bipinnatus*, and *B. alba* (Fig. 6A, Supplementary Table S26). By phylogenetic analysis, we inferred the evolutionary history of the inulin genes in related Asteraceae plants, such as *Cichorium intybus* and *A. lappa* (Supplementary Fig. S11). In addition, we used the publicly available RNA-seq dataset of *D. pinnata* and full-length transcriptome of *C. bipinnatus* and *B. alba* to validate these genes (Supplementary Figs S12 and S13) and found that the 2 synthesis genes (*1-SST* and *1-FFT*) expressed more in *D. pinnata* than the other 2 plants, which is consistent with the fact that *D. pinnata* produces much more inulin than *C. bipinnatus* and *B. alba*. For the 2 biosynthesis genes *1-SST* and *1-FFT*, only 1 copy was found in *C. bipinnatus* and 2 copies were discovered in *D. pinnata* and *B. alba* due to the recent WGDs. Meanwhile, the *1-SST* and *1-FFT* genes formed a biosynthetic gene cluster (BGC) in *D. pinnata* and *C. bipinnatus* (Fig. 6B), which may contribute to inulin production in the 2 species [53]. However, the *1-SST* and *1-FFT* genes are located on different chromosomes (Chr 10 and Chr 11) in *B. alba*, and there are 5 pseudogenized *1-FFT* genes near the *1-SST* genes (Fig. 6B and Supplementary Fig. S14). Only some short fragmented micro-synteny was detected between Chr10 and Chr11, and the *1-SST* and *1-FFT* genes located in one of these syntenic fragments are likely to be the relics of WGD-2 (Supplementary Fig. S15). On the other hand, both *D. pinnata* and *B. alba* have 2 copies of breakdown genes *1-FEH1* and *1-FEH2*, which were derived from their recent species-specific whole-genome duplications. However, 1 copy of *1-FEH1* and 1 copy of *1-FEH2* have become pseudogenes in *D. pinnata* (Fig. 6C, D, Supplementary Table S27, Supplementary Figs. S16 and S17). The pseudogenization of *1-FEH1* and *1-FEH2* genes may decrease the breakdown of inulin, thus preserving more inulin in the tuber for *D. pinnata*.

### Missing 3 key residues “DPN” in 1-FFT proteins of *C. bipinnatus* and *B. alba*

As 1-FFT is critical for the inulin polymerization, we compared the difference of the 1-FFT protein for the 3 plants of the tribe Coreopsideae. First, we aligned the protein sequences of these *1-FFT* genes together (Supplementary Fig. S18). According to the resulting multiple sequence alignment and the knowledge from previous studies [54], we determined the catalytic triads for these proteins (Fig. 6E). The g6466 of *C. bipinnatus* and the g141421 of *B. alba* missed 3 key amino acids “DPN” in the first motif of the catalytic triad, which could impact the function of these proteins.

Furthermore, we predicted the 3-dimensional structures of the 1-FFT proteins by AlphaFold [55]. Then, through a superposition of these protein structures, we found that all of them have very similar structures, containing an N-terminal β-propeller domain, a C-terminal β-sheet domain, and a cleft between the 2 domains. For the catalytic triads, the loss of 3 residues greatly changed the conformation of 1-FFT (Fig. 6E). Importantly, the first residue “D” in the missing “DPN” has been reported acting as the nucleophile and being involved in the catalyzing process [56]. All plant fructan-metabolizing enzymes belong to the glycoside hydrolase 32 (GH32) family, and the residues at the catalytic triads are conserved among these enzymes [57]. Hence, the loss of the 3 residues in 1-FFTs of *C. bipinnatus* and *B. alba* may severely influence their inulin polymerization function.

## Discussion

In this study, we present the high-quality reference genomes of 3 important plants *D. pinnata, C. bipinnatus*, and *B. alba* in the tribe Coreopsideae of Asteraceae. Thanks to the long and highly accurate PacBio HiFi data and long-range Hi-C data, we achieved the chromosome-scale haplotype-resolved assemblies. To get the final telomere-to-telomere reference genomes for these plants, Oxford Nanopore Technologies ultra-long reads or other advanced technologies may be helpful. With the comprehensive annotation of TEs, we found that LTRs, especially *Gypsy*, were the most abundant LTRs in *D. pinnata*, which were significantly expanded within the past 1 million years and largely responsible for the larger genome size of *D. pinnata*. Because TEs can modify regulatory networks and create new genes [58], the recent expansion of the *Gypsy* TEs may also contribute to the wide diversity of cultivars of *D. pinnata*.

Phylogenomic analysis clarified that the genera *Bidens* and *Cosmos* are close sisters, which diverged from each other ∼7.00 Mya. The genus *Dahlia* separated from the last common ancestor of the genera *Bidens* and *Cosmos* 14.62 Mya. With haplotype-resolved chromosomes, we revealed that *D. pinnata* is an autotetraploid plant derived from a recent whole-genome duplication. A previous study using genomic in situ hybridization has shown that meiosis chromosome pairing occurs both between and within parental genomes [59], supporting our conclusion. We also showed that *B. alba* is an allotetraploid, which originated from the hybridization of 2 ancestor species followed by chromosome doubling. The hybridization event was relatively far from the current, so *B. alba* exhibited diploid inheritance features in the meiosis process [34, 60].

Compared with *C. bipinnatus* and *B. alba*, the tubers of *D. pinnata* are much larger and composed of a high proportion of inulin-type fructan. Within the genomes, we identified all copies of the key functional genes and transcription factors for inulin biosynthesis and degradation. We found 1 of the 2 copies of *1-FEH1* and *1-FEH2* genes in *D. pinnata* became pseudogenes, which may decrease the breakdown of inulin and thus increase the inulin accumulation in the tuber of *D. pinnata*. The pseudogenization of *1-FEH1* and *1-FEH2* genes may also influence the degree of inulin polymerization. On the other hand, the 2 copies of *1-FFT* genes in *D. pinnata* preserved canonical residues in the catalytic triads, while the 1-FFT of *C. bipinnatus* and 1 of the 2 copies of 1-FFT in *B. alba* lost 3 residues in the first motif of the catalytic triads, which may severely influence the inulin biosynthesis and probably explain why the efficiency of inulin production of *C. bipinnatus* and *B. alba* is much lower than that of *D. pinnata*. In addition to plants in Coreopsideae, further research could use all available Asteraceae plants and plants in a closely related outgroup, such as *Scaevola taccada* in Goodeniaceae [61], to explore why Asteraceae plants take inulin as the primary reserve carbohydrates.

*D. pinnata* and *C. bipinnatus* are popular flowers all over the world [3, 62, 63], and *B. alba* is a traditional medical plant, although it is also recognized as an invasive plant [60]. The genomic resources of *D. pinnata* and *C. bipinnatus* presented in this study will promote the studies on regulating mechanisms of flower shapes, colors, and senescence and benefit the flower industry by breeding more popular and long-vase life cultivars of flowers. In addition, the genomic resources of *B. alba* will help develop more efficient and green methods to prevent the invasion of this weed.

## Methods

### Plant materials, DNA, and RNA sequencing

The tubers of the *D. pinnata* cultivar “Kelvin floodlight” were purchased from a horticultural company located in Chuzhou, Anhui province, China. The seeds of *C. bipinnatus* were acquired from a seed company located in Shuyang, Jiangsu province. The seeds of *B. alba* were collected from the riverside in Shenzhen, Guangdong province. The tubers and seeds were grown on the farm of the Agricultural Genomics Institute at Shenzhen, Chinese Academy of Agricultural Sciences. For *B. alba*, fresh tips of plant roots were collected and analyzed using FISH for karyotype analysis.

Tender fresh leaves were collected from 1 plant per species. Fifty-day-old *D. pinnata*, 50-day-old *C. bipinnatus*, and 40-day-old *B. alba* were used for sequencing. For PacBio HiFi sequencing (Pacific Biosciences), the high-quality genomic DNA was extracted using the Qiagen DNeasy Plant Mini Kit (Qiagen). The quality of DNA was assessed using 0.75% agarose gel electrophoresis, Nanodrop, and the Qubit fluorimeter (Thermo Fisher). SMRTbellTM Express Template Prep Kit 2.0 was used to create a 20-kb DNA SMRTbell library. The library was sequenced using the PacBio Sequel II platform. For genomic DNBSEQ sequencing of *D. pinnata*, a library with a 500-bp insert length was prepared without using PCR by following standard protocols. Paired-end reads (PE150) were sequenced on DNBSEQ-T7 (RRID:SCR_017981). For Hi-C sequencing, the fresh leaves were shredded and cross-linked with 2% formaldehyde. The DNA was then digested using the MboI enzyme, followed by biotin labeling the ends of fragments. Fragmented DNA was ligated and sheared. The biotin-labeled fragments were enriched with streptavidin beads and used to build a sequencing library. This library was sequenced on an Illumina HiSeq 2500 (RRID:SCR_016383) by PE150 bp (Illumina). Moreover, for full-length transcriptome sequencing (PacBio Iso-seq), high-quality RNA was extracted by the Qiagen kit from root, stem, leaf, and flower tissues per species. The standard protocol was used to construct a sequencing library with an insertion size of 0.5–6 kb. This library was sequenced on the PacBio Sequel II platform (RRID:SCR_017990).

### Genome assembly

To estimate genome size, we used Kmerfreq [64] to count *k*-mer frequency based on DNBSEQ reads (for *D. pinnata*) and HiFi reads (for *C. bipinnatus* and *B. alba*). Genome size estimation was accomplished using the concept of GCE (RRID:SCR_017332) [65].

For contigs assembly, we used hifiasm (RRID:SCR_021069) v0.18.5 to assemble contigs by integrating HiFi and Hi-C sequencing reads per species [36]. Because *D. pinnata* and *B. alba* are tetraploid plants, and hifiasm is designed to assemble a diploid genome, the resulting haplotype 1 and haplotype 2 were not properly separated. Thus, we merged 2 haplotypes, and the merged assembly contained both subgenomes and allelic genomes. For *C. bipinnatus*, a diploid plant, 1 haplotype can be taken as the reference assembly. Due to the heterozygous character of *C. bipinnatus*, we used purge_dups (RRID:SCR_021173) v1.2.6 with parameter settings “-a 0.70” and “-f 0.65” to remove haplotypic duplications [66].

To filter organelle genomes, the chloroplast and mitochondrion genomes of Coreopsideae plants were downloaded from the NCBI database. We used minimap2 (RRID:SCR_018550) v2.24 with parameters “-cx asm20” to align the contigs of *D. pinnata, C. bipinnatus*, and *B. alba* onto the reference organelle genomes of Coreopsideae plants [67]. Contigs composed of more than 95% organelle sequences and less than 5% divergence were filtered to get the nucleus genomes. The completeness of the contig assemblies was evaluated by BUSCO (RRID:SCR_015008) v5.4.4 with the embryophyta lineage database of OrthoDB (RRID:SCR_011980) v10 [68].

To ensure the contigs of 2 tetraploid genomes were not merged contigs (i.e., similar sequences from subgenomes or allelic genomes were merged into 1 contig in the assembly), we checked the average coverage of contigs based on HiFi data. First, we used minimap2 with parameters “-ax map-hifi -I 16G” to map HiFi reads onto the contigs. Then, we used samtools (RRID:SCR_002105) v1.9 with parameters “-F 0×104” to filter unmapped records and nonprimary alignments; we used bedtools (RRID:SCR_006646) v2.30.0 with parameters “genomecov -ibam -bg” to calculate the coverage of contigs. Taking the resulting bedGraph files as input, an in-house Perl script was used to get the average coverage of each contig.

As suggested by YaHS (RRID:SCR_022965), the state-of-the-art Hi-C scaffolding tool, we used the Arima Genomics’ mapping pipeline [69] to process Hi-C reads for each species. The pipeline mapped reads in single-end read mode, filtered chimeric joined reads, paired reads, and removed PCR duplications. After that, we used YaHS v1.2a.1 with parameters “–no-contig-ec -e GATC” to construct chromosome-scale scaffolds based on the above clean Hi-C mapping results [37]. For tetraploid plants *D. pinnata* and *B. alba*, the contig assembly contained 4 haplotypes, and YaHS could group contigs from 4 homologous chromosomes together. Finally, based on the Hi-C signal features and the telomere information, we manually curated the scaffolds on Juicebox (RRID:SCR_021172) [38]. The chromosomes of tetraploid plants from the same homologous chromosome group would form a strong diagonal Hi-C interaction not only within a chromosome but also between chromosomes. This feature is very helpful for manual curation.

### Repeats analysis

For tandem repeats, we utilized Tandem Repeats Finder (RRID:SCR_022193) v4.09 to identify tandem repeats [70]. We also identified candidate telomeres on the scaffolds based on the TRF results and telomere sequence of “CCCTAAA” and “TTTAGGG.” For TEs, 3 steps were used to find them: (i) structure-intact TEs (including LTR-RT, DNA transposon, Helitron) were predicted by Extensive *de novo* TE Annotator (EDTA) v1.9.9, and simultaneously the intact TE library was generated [71]; (ii) incomplete TEs were recognized by homology searching against the above intact TE library, Repbase database (RRID:SCR_021169) v26.05 (plant lineage) with RepeatMasker (RRID:SCR_012954), and protein-coding TE database with RepeatProteinMask v4.1.2; and (iii) a *de novo* TE library was built based on the masked genome with identified TEs (all above intact and incomplete TEs) using RepeatModeler (RRID:SCR_015027) v2.0.2, and transposable elements representation learner was used to classify the TE sequences in the library [72]. The TE sequences that could be classified as any of the TE classes were used by RepeatMasker to identify the remaining TEs in the genome. Finally, we merged all TE annotations, removed redundancy, and got the final TE annotation.

For estimation of LTR insertion times, we extracted sequences of LTRs based on intact TEs annotated by EDTA and used MUSCLE to align long terminal repeats of each LTR. The pairwise distances from the LTRs were estimated by the APE package in R with the K80 model. The sequence divergence (*d*) of long terminal repeats of each LTR and the mutation rate of sunflower (*r* = 1 × 10^−8^) were used to calculate the times with the equation *T = d/2r* [20].

### Gene prediction

To predict protein-coding genes, we used Augustus with hints on gene structures, such as intron, exon, part of exon, part of CDS, and gene start, derived from PacBio Iso-seq, RNA-seq, and protein homology [73]. For Iso-seq, we used lima with parameters “–isoseq –peek-guess” to clip the primer sequence. Then, isoseq3 (RRID:SCR_022749) v3.4.0 was used to refine and cluster CCS reads to get high-quality transcripts. We used GMAP (RRID:SCR_008992) v2020-10-27 to map the high-quality full-length transcripts to assemblies, and the result files were filtered and transferred into hints file by blat2hints.pl in AUGUSTUS (RRID:SCR_008417) [39]. For homology searches, we used miniprot v0.10 with parameters “-ut50 –gtf -I” to map proteins of *H. annuus, S. sonchifolius*, and *S. rebaudiana* to the genomes [74]. After that, we used aln2hints.pl in GALBA v2022-11-07 with parameters “–prg=miniprot” to filter the resulting alignment files and transfer them into hints files [75]. For Illumina RNA-seq analysis, datasets of *D. pinnata* and *C. bipinnatus* were downloaded from NCBI, and STAR (RRID:SCR_004463) v2.7.10 was used to map reads to the genomes. The result bam files were filtered by filterBam in Augustus with parameters “–uniq –paired –pairwiseAlignments.” After sorting by samtools, bam2hints in Augustus was used to generate hints files with parameters “–intronsonly.” After comparison of the training parameters derived from BUSCO assessment with eudicots_odb10 and embryophyta_odb10 lineage databases, we chose the parameter files generated with eudicots_odb10 as the species-specific gene prediction models, because we can get more complete gene sets [68]. Based on these gene prediction parameters, integrating hints from Iso-seq, homology search, RNA-seq, and TE soft-masked genomes, Augustus v3.5.0 was used with parameters “–hintsfile=hintsfile.gff –gff3=on –softmasking=on –codingseq=on –noInFrameStop=true” to predict transcripts of genes.

### Functional annotation

To assign gene functions for the gene sets, we used InterProScan (RRID:SCR_005829) v5.52–86 to annotate genes and got Gene Ontology (GO) terms [76]. We also aligned the protein sequences of genes with KEGG, NR, and Swiss-Prot databases by Diamond, an alternative replacement of BLAST, using 1e−5 as a cutoff and got the best hit. We used PlantTFDB (RRID:SCR_003362) v5.0 to annotate transcription factors [40]. Moreover, tRNAscan-SE (RRID:SCR_008637) v2.0 was used to find tRNAs with default parameters [77], and cmscan from infernal (RRID:SCR_011809) v1.1.4 [78] was used to find other ncRNAs based on Rfam (RRID:SCR_007891) v14 [79]. RNAmmer (RRID:SCR_017075) v1.2 [80] with parameters “-S euk -m lsu,ssu,tsu” was used to annotate 8S, 18S, and 28S rRNA.

### Evolution analysis

For orthogroup construction, we used datasets of 13 species, including *D. pinnata, C. bipinnata, B. alba, A. artemisiifolia* [21], *H. annuus* [20], *S. rebaudiana* [22], *M. micrantha* [23], *S. sonchifolius* [15], *C. nankingense* [41], *A. annua* [42], *C. canadensis* [43], *A. lappa* [15], and *Vitis vinifera* (outgroup). We used OrthoFinder (RRID:SCR_017118) v2.5.5 with parameters “-M msa -A mafft -T fasttree -1 -y -S diamond_ultra_sens” to build orthogroups [44]. For *D. pinnata* and *B. alba*, genes on 2 haplotypes (A1 and A3 for *D. pinnata*; A1 and B1 for *B. alba*) were employed. OrthoFinder called Species Tree from All Genes to build an unrooted species tree [81] and used Species Tree Root Inference from Gene Duplication Events to root this tree [82]. In addition to this species tree, we also constructed a new species tree based on the traditional single-copy genes concatenation method. First, we identified single-copy genes across species according to the results of phylogenetic hierarchical orthogroups from OrthoFinder. Because *D. pinnata, B. alba*, and *S. sonchifolius* are tetraploids, we randomly chose 1 gene from duplicated genes. Second, we used MUSCLE (RRID:SCR_011812) v3.8.31 to do multiple sequence alignment [83] and employed RAxML-NG to construct a species tree with parameters “–all –model GTR+G –tree pars 10 –bs-trees 100 –outgroup Vitis_vinifera” using *V. vinifera* as the outgroup [84].

To build a time tree, we used MEGA-CC with calibration time of 36.6–45.1 Mya between *H. annuus* and *A. lappa* from TimeTree (RRID:SCR_021162), 8 gamma distribution and general time-reversible model according to the multiple sequence alignment (MSA), and the species tree [85]. To detect syntenic gene blocks, we employed MCScanX (RRID:SCR_022067) to find collinear genes [86]. Based on the collinear genes, KaKs_Calculator (RRID:SCR_022068) v2.0 with parameters “-m GMYN” was used to estimate the synonymous substitution rate (*Ks*) for ortholog genes [87]. Circos was used to plot the circos plot [88]. For *D. pinnata* and *B. alba*, we also employed JCVI [89] to detect collinearity between different haplotypes. The phylogenetic relationships of chromosomes within each homologous chromosome group were inferred based on the single-copy gene concatenation method. We also employed SubPhaser to phase subgenomes by based on subgenome-specific *k*-mers [90].

### Genome mining for inulin metabolism and regulation genes

To identify the inulin metabolism genes, we collected the sequences of published genes in Asteraceae species involved in the synthesis and degradation of inulin. In total, three 1-SST (APM87555.1, AFB83198.1, CAA08812.1), four 1-FFT (APM87557.1, QJQ28876.1, AAD00558.1, CAA04120.2), two 1-FEH1 (AJW31155.1, CAC19366.1), and five 1-FEH2 (APM87560.1, BAL73222.1, AAP85536.1, AIP90173.1, AJW31156.1) genes were collected and checked in multiple sequence alignments. Then, we collected the published transcription factor genes (CiMYB3: ARO49672.1, CiMYB5: ARO49674.1, CiMYB17: ARO49669.1) from chicory that have been reported to play key roles in inulin metabolism regulation [51, 52]. These genes were aligned to the reference gene sets of *D. pinnata, C. bipinnatus*, and *B. alba* by BLAST (RRID:SCR_004870) v2.3.0 with parameters “blastp -task blastp -evalue 1e-5.” The genes with sequence similarity over 75% and length coverage over 75% were taken as potential inulin metabolism genes in each species. For confirmation, the orthogroups that contained these genes were obtained and checked. Finally, possible pseudo-genes or missed genes were identified through Exonerate (RRID:SCR_016088) v2.4.0 by aligning the collected public genes to the reference genomes [91]. Then, we used MUSCLE to do MSA with the candidate genes and pyBoxshade [92] to show the MSAs. The gene tree of each gene family was built by RAxML-NG with parameters “–all –model GTR+G –tree pars 20 –bs-trees 1000” based on MSAs of CDS, and the tree figures were plotted by FigTree (RRID:SCR_008515) v1.4.4.

To evaluate the gene expression in *D. pinnata*, we used publicly available RNA-seq datasets and employed STAR v2.7.1a to map them to the reference genes. In the PRJNA811758, there are 3 tissues (leaf, stem, and root) with each 3 biological repeats in the normal growing state. The reads count of each gene was acquired from the mapping results, and our in-house Perl program was used to calculate transcripts per millions (TPMs). For gene expression of *C. bipinnatus* and *B. alba*, we used minimap2 to align the full-length transcriptome data to the gene sets and used in-house Perl program to calculate TPMs. For the gene expression heatmap, we used the pheatmap and RColorBrewer packages of R v.4.2.0 to draw figures based on the TPM. Additionally, the 3-dimensional structure for 1-FFT genes was predicted by Alphafold v2.1.0 [55] and demonstrated by open-source PyMOL (RRID:SCR_000305) v2.5.0.

## Additional Files

**Supplementary Table S1**. Sequencing statistics of *D. pinnata, B. alba*, and *C. bipinnatus*.

**Supplementary Table S2**. Statistics of reference organelle genomes used for filtering sequence from organelle.

**Supplementary Table S3**. Statistics of filtered organelle genome sequence.

**Supplementary Table S4**. Statistics of contig assemblies for *D. pinnata, C. bipinnatus*, and *B. alba*.

**Supplementary Table S5**. Statistics of Hi-C reads mapping for *D. pinnata*.

**Supplementary Table S6**. Statistics of Hi-C reads mapping for *C. bipinnatus*.

**Supplementary Table S7**. Statistics of Hi-C reads mapping for *B. alba*.

**Supplementary Table S8**. Statistics of scaffold assemblies for *D. pinnata, C. bipinnatus*, and *B. alba*.

**Supplementary Table S9**. Positions of telomeres on the chromosomes of *D. pinnata*.

**Supplementary Table S10**. Positions of telomeres on the chromosomes of *C. bipinnatus*.

**Supplementary Table S11**. Positions of telomeres on the chromosomes of *B. alba*.

**Supplementary Table S12**. BUSCO assessment of the genomes of *D. pinnata, C. bipinnatus, B. alba*, and other related species.

**Supplementary Table S13**. Public RNA-seq dataset statistics.

**Supplementary Table S14**. Statistics of gene prediction evidence from full-length cDNA mapping.

**Supplementary Table S15**. Statistics of gene prediction evidence from homology alignment.

**Supplementary Table S16**. Statistics of gene prediction evidence from RNA-seq mapping.

**Supplementary Table S17**. Gene statistics of *D. pinnata, C. bipinnatus, B. alba*, and closely related plant species.

**Supplementary Table S18**. BUSCO assessment of the gene sets of *D. pinnata, C. bipinnatus, B. alba*, and other related species.

**Supplementary Table S19**. Statistics of functional annotation for the gene sets based on different databases.

**Supplementary Table S20**. Statistics of annotated transcription factors.

**Supplementary Table S21**. The summary of annotated tRNAs.

**Supplementary Table S22**. The summary of annotated rRNAs and other ncRNAs.

**Supplementary Table S23**. Statistics of annotated transposable elements.

**Supplementary Table S24**. Statistics of closely related genomes.

**Supplementary Table S25**. Statistics of orthogroups in different plants defined by OrthoFinder.

**Supplementary Table S26**. Gene ID of inulin metabolism genes and transcription factors.

**Supplementary Table S27**. The Pfam domain annotation of 1-FEH1 and 1-FEH2.

**Supplementary Fig. S1**. Karyotype of *B. alba* by fluorescence in situ hybridization (FISH) technology.

**Supplementary Fig. S2**. Distribution of *k*-mer (K = 19) frequency in sequencing reads of the 3 Coreopsideae plants.

**Supplementary Fig. S3**. Hi-C heatmap of chromosomes for *D. pinnata* (A), *C. bipinnatus* (B), and *B. alba* (C).

**Supplementary Fig. S4**. Comparison of gene characteristics among closely related Asteraceae genomes.

**Supplementary Fig. S5**. The estimated insertion time of different types of long terminal repeats (LTRs) in *C. bipinnatus* (A) and *B. alba* (B).

**Supplementary Fig. S6**. Species tree built by OrthoFinder.

**Supplementary Fig. S7**. Dot plot of chromosomes alignment for *D. pinnata*.

**Supplementary Fig. S8**. Subgenome (SG) phasing of the *D. pinnata* genome by SubPhaser.

**Supplementary Fig. S9**. Dot plot of chromosomes alignment for *B. alba*.

**Supplementary Fig. S10**. Subgenome (SG) phasing of the *B. alba* genome by SubPhaser.

**Supplementary Fig. S11**. Gene tree constructed of inulin genes and transcription factor genes.

**Supplementary Fig. S12**. Heatmap of inulin metabolism genes and transcription factor genes in *D. pinnata*.

**Supplementary Fig. S13**. Heatmap of inulin metabolism genes and transcription factor genes in *D. pinnata, C. bipinnatus*, and *B. alba*.

**Supplementary Fig. S14**. Multiple sequence alignment of 1-FFT genes (*g142078.t1* and *g141421.t1*) and pseudogenes (*g31433.t1, g31432.t1, g87332.t1, g87331.t1*, and *g87333.t1*) of *B. alba*.

**Supplementary Fig. S15**. Dot plot of synteny gene blocks between A1_10 and A1_11, B1_11 in *B. alba*.

**Supplementary Fig. S16**. Multiple sequence alignment of 1-FEH1 for closely related species.

**Supplementary Fig. S17**. Multiple sequence alignment of 1-FEH2 for closely related species.

**Supplementary Fig. S18**. Multiple sequence alignment of 1-FFT genes of *D. pinnata, C. bipinnatus*, and *B. alba*.

## Abbreviations

1-FFT: fructan/fructan 1-fructosyl transferase; 1-SST: sucrose/sucrose 1-fructosyl transferase; BGC: biosynthetic gene cluster; BLAST: Basic Local Alignment Search Tool; BUSCO: Benchmarking Universal Single-Copy Orthologs; FISH: fluorescence in situ hybridization; GO: Gene Ontology; Iso-seq: isoform sequencing; ITS: internal transcribed spacer; KEGG: Kyoto Encyclopedia of Genes and Genomes; LTR: long terminal repeat; MSA: multiple sequence alignment; Mya: million years ago; NCBI: National Center for Biotechnology Information; ncRNA: noncoding RNA; RNA-seq: RNA sequencing; rRNA: ribosomal RNA; TF: transcription factor; TPM: transcripts per million; tRNA: transfer RNA; WGD: whole-genome duplication.

## Supplementary Material

giae032_Supplemental_File

giae032_GIGA-D-23-00392_Original_Submission

giae032_GIGA-D-23-00392_Revision_1

giae032_Response_to_Reviewer_Comments_Original_Submission

giae032_Reviewer_1_Report_Original_Submission Daniel Jones -- 2/5/2024

giae032_Reviewer_2_Report_Original_Submission Xiaozeng Yang -- 2/10/2024

giae032_Reviewer_2_Report_Revision_1 Xiaozeng Yang -- 3/23/2024

## Data Availability

All the whole-genome sequencing data that support this project have been deposited at NCBI with BioProject ID PRJNA1055312 and China National Genomics Data Center with the Project ID PRJCA017572. The genome assemblies, gene annotations, and other resources are available at Zenodo [93] and AGIS website [94]. Customized codes used in this project can be found on GitHub [95]. All additional supporting data are available in the *GigaScience* repository, GigaDB [96].
