# Supplementary material for: The genomes of Dahlia pinnata, Cosmos bipinnatus, and Bidens alba in tribe Coreopsideae provide insights into polyploid evolution and inulin biosynthesis
Source: Gigascience. 2024 Jun 13;13:giae032. doi: 10.1093/gigascience/giae032 (PMC11170221; doi:10.1093/gigascience/giae032)
Supplement: giae032_Supplemental_File [file giae032_supplemental_file.docx]

**Supplementary Figures**

B

A


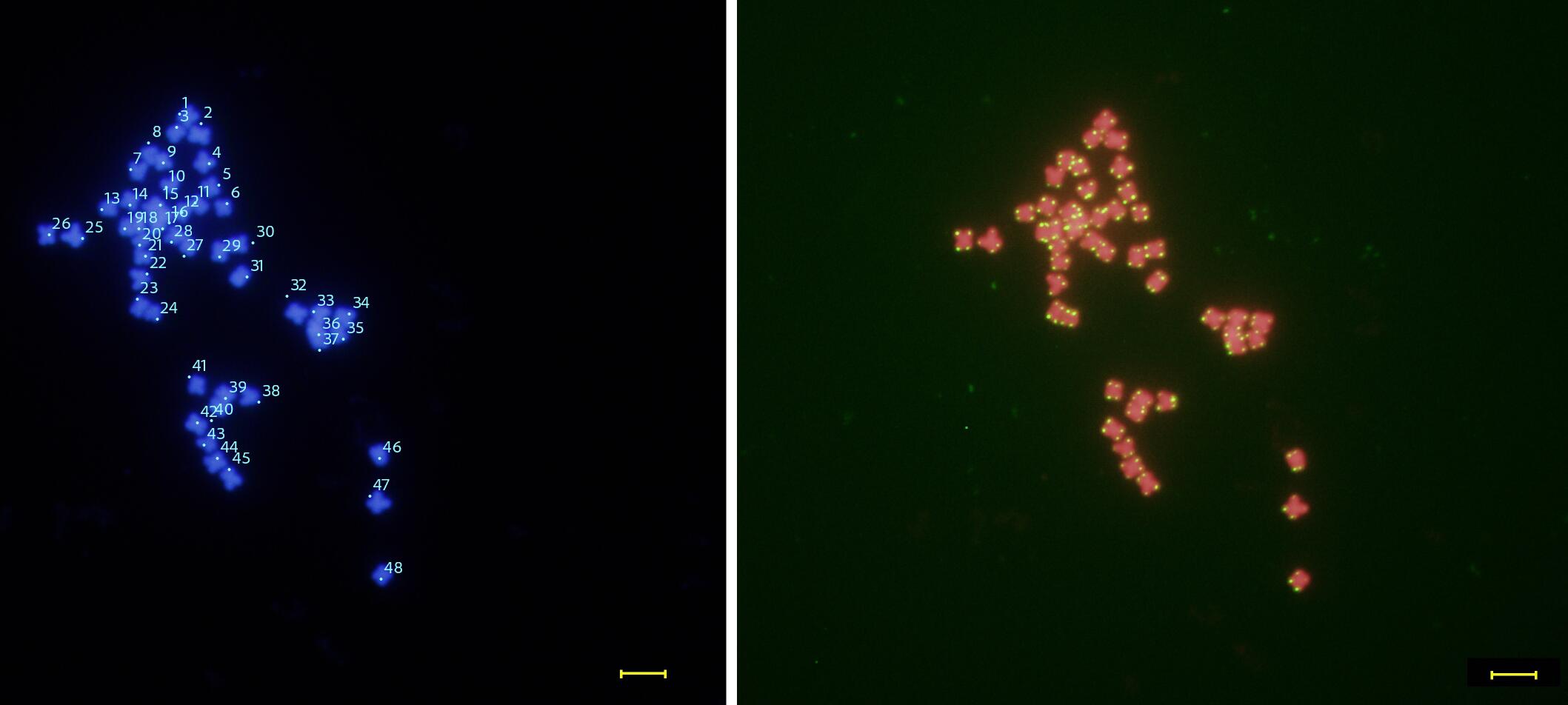


Figure S1. Karyotype of *B. alba* by fluorescence in situ hybridization (FISH) technology. The chromosomes were dyed with DAPI (A) and Texas Red (B), and then karyotype photos were obtained by high-resolution fluorescence microscope and CCD camera. From the figure, we can ensure that there are 48 chromosomes in *B. alba*.


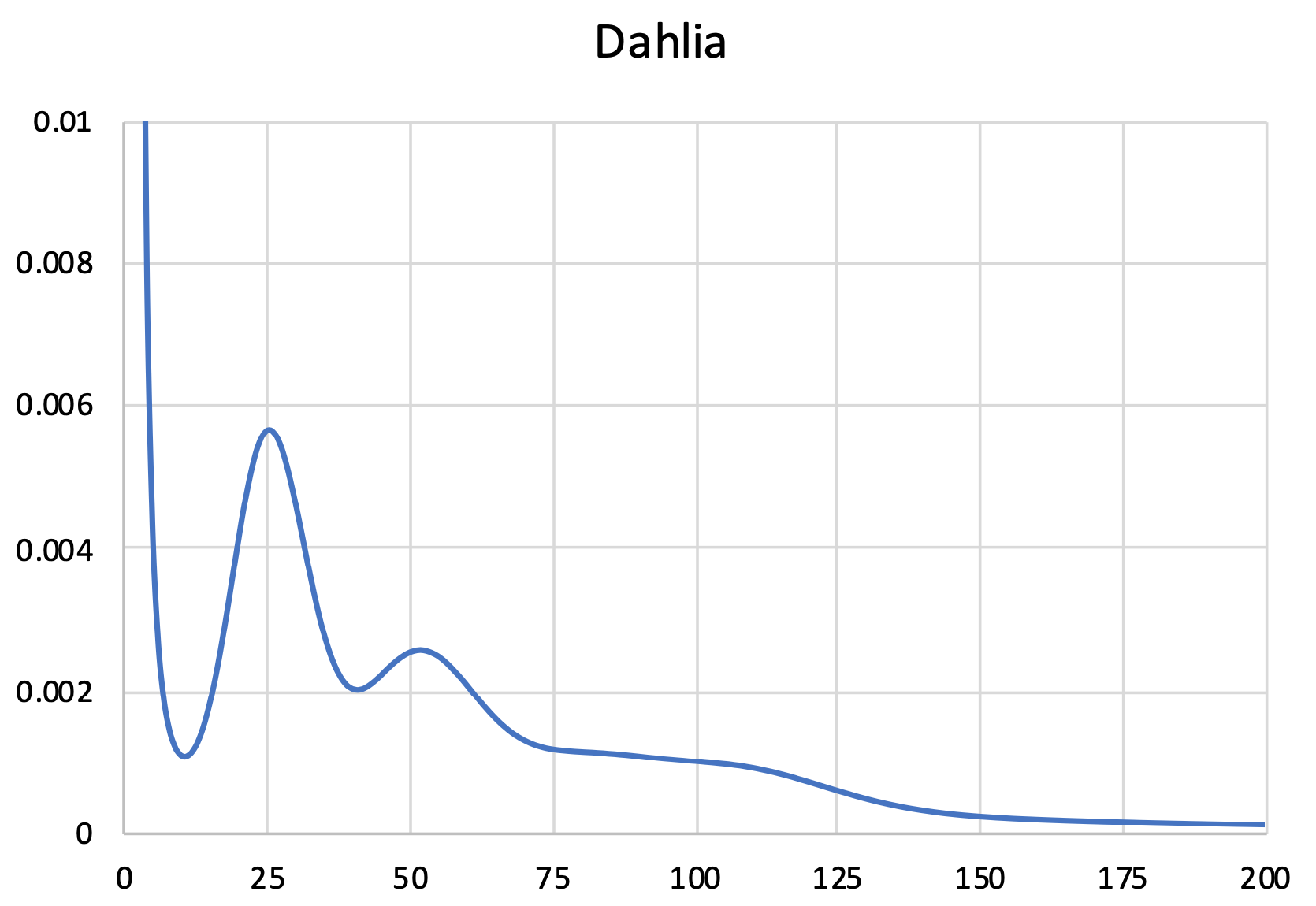

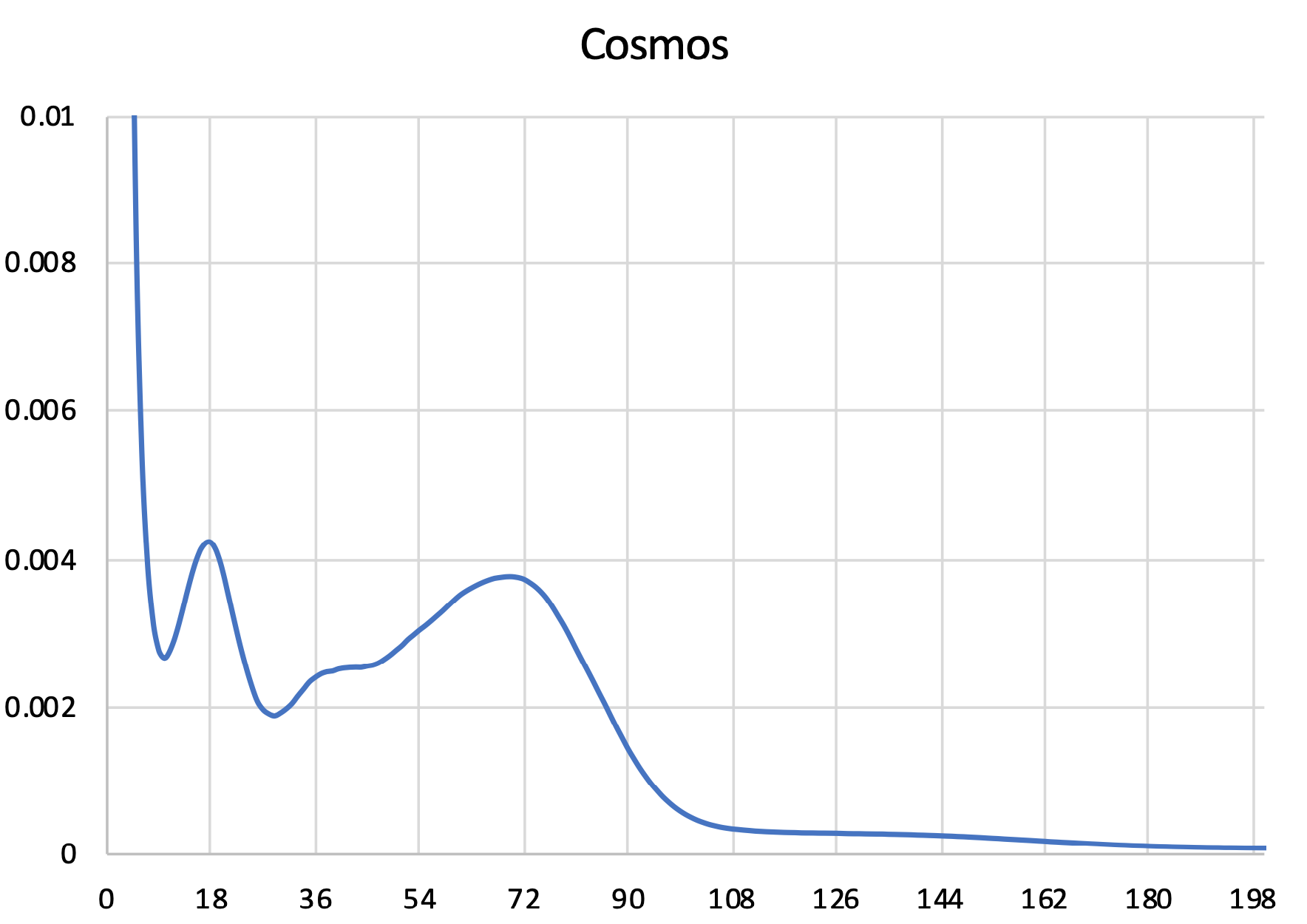


C

B

A


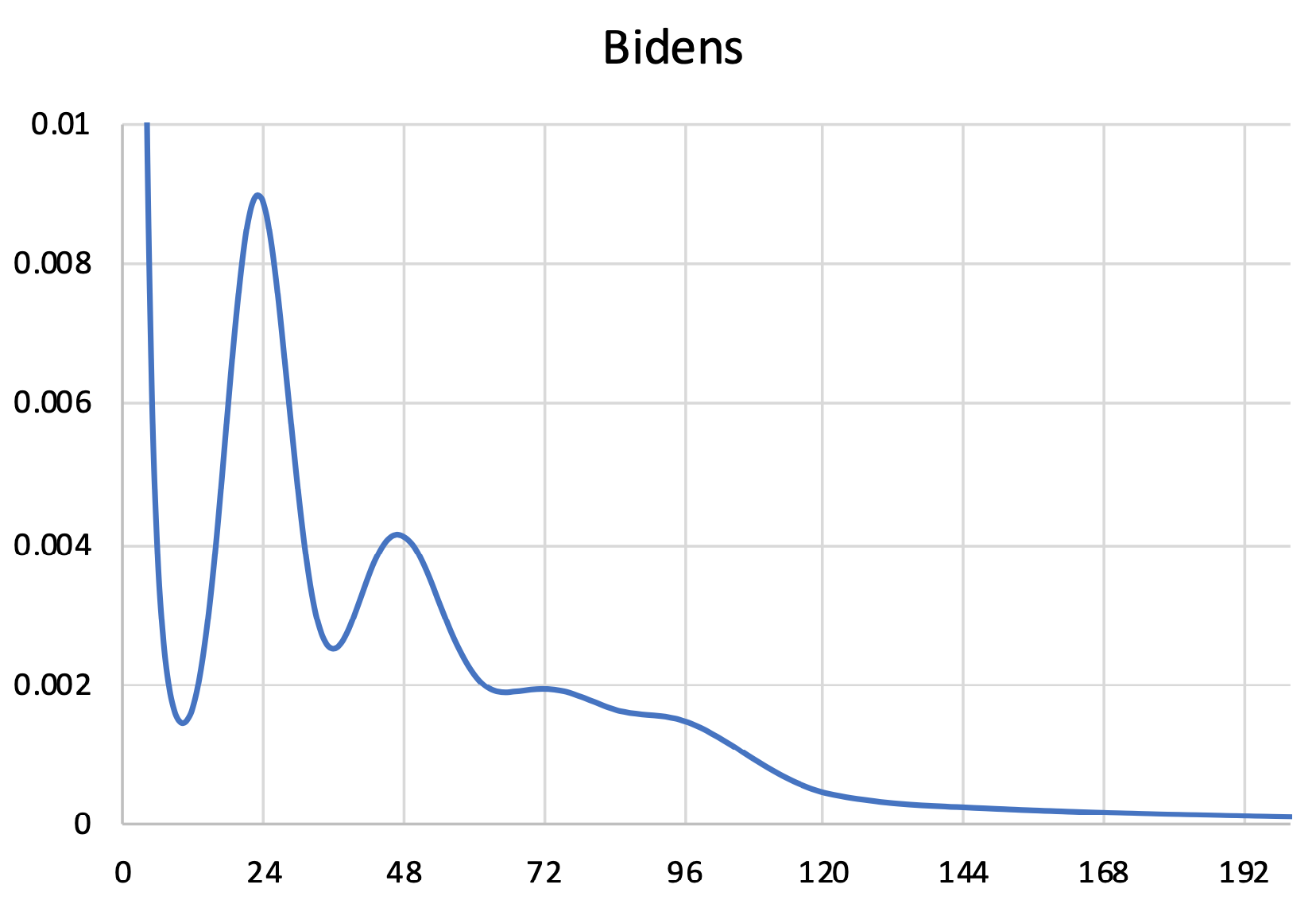


Figure S2. Distribution of K-mer (K = 19) frequency in sequencing reads of the three Coreopsideae plants. (A) Distribution of K-mer frequency for *D. pinnata* from DNBSEQ sequencing. (B) Distribution of K-mer frequency for *C. bipinnatus* from PacBio HiFi sequencing. (C) Distribution of K-mer frequency for *B. alba* from PacBio HiFi sequencing. The K-mer frequency peak with arrow is the main peak. Based on the K-mer distributions, we know that *D. pinnata*, *C. bipinnatus* and *B. alba* have high heterozygosity rates; peak values are 52, 70 and 47, and the estimated genome sizes (1C) are 3.98, 1.08 and 1.93 Gb, respectively.


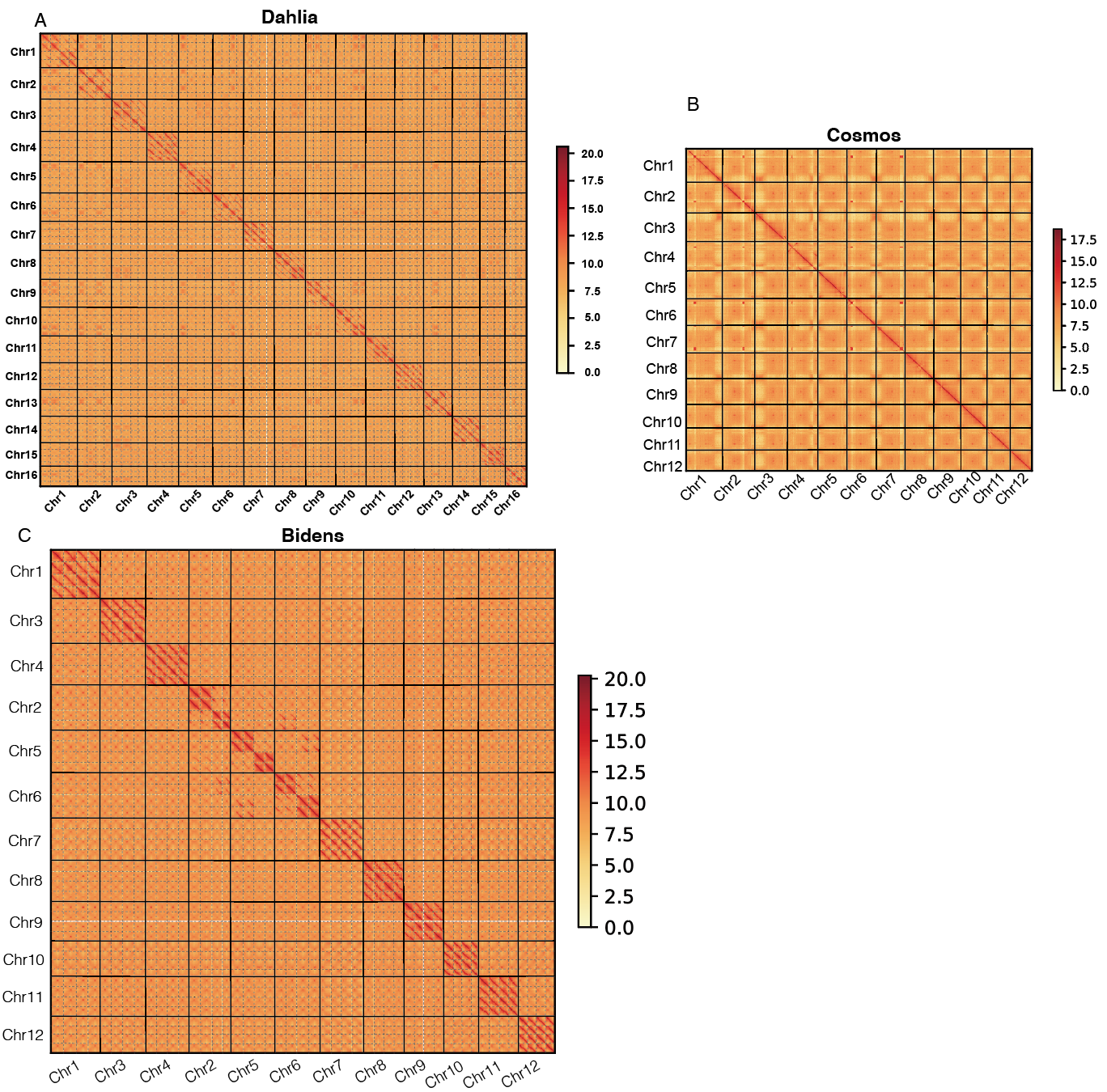


Figure S3. Hi-C heatmap of chromosomes for *D. pinnata* (A), *C. bipinnatus* (B) and *B. alba* (C). The colour represents Log2(links number), and the resolution (bin size) is 5-Mb, 1-Mb, and 5-Mb for *D. pinnata*, *C. bipinnatus* and *B. alba*, respectively. Links number is the number of Hi-C links falling into the two analyzed genomic bins. For *D. pinnata*, the auto-tetraploid chromosomes are shown in the order of A1, A2, A3 and A4 for each homologous chromosome group. For *B. alba*, the allo-tetraploid chromosomes are shown in the order of A1, A2, B1 and B2 for each homologous chromosome group. Note that the reference genomes for each species were shown. *C. bipinnata* is diploid, so the haploid genome was used as reference genome. In contrast, *D. pinnata* and *B. alba* are tetraploids, the heatmap showed Hi-C signals among all chromosomes.


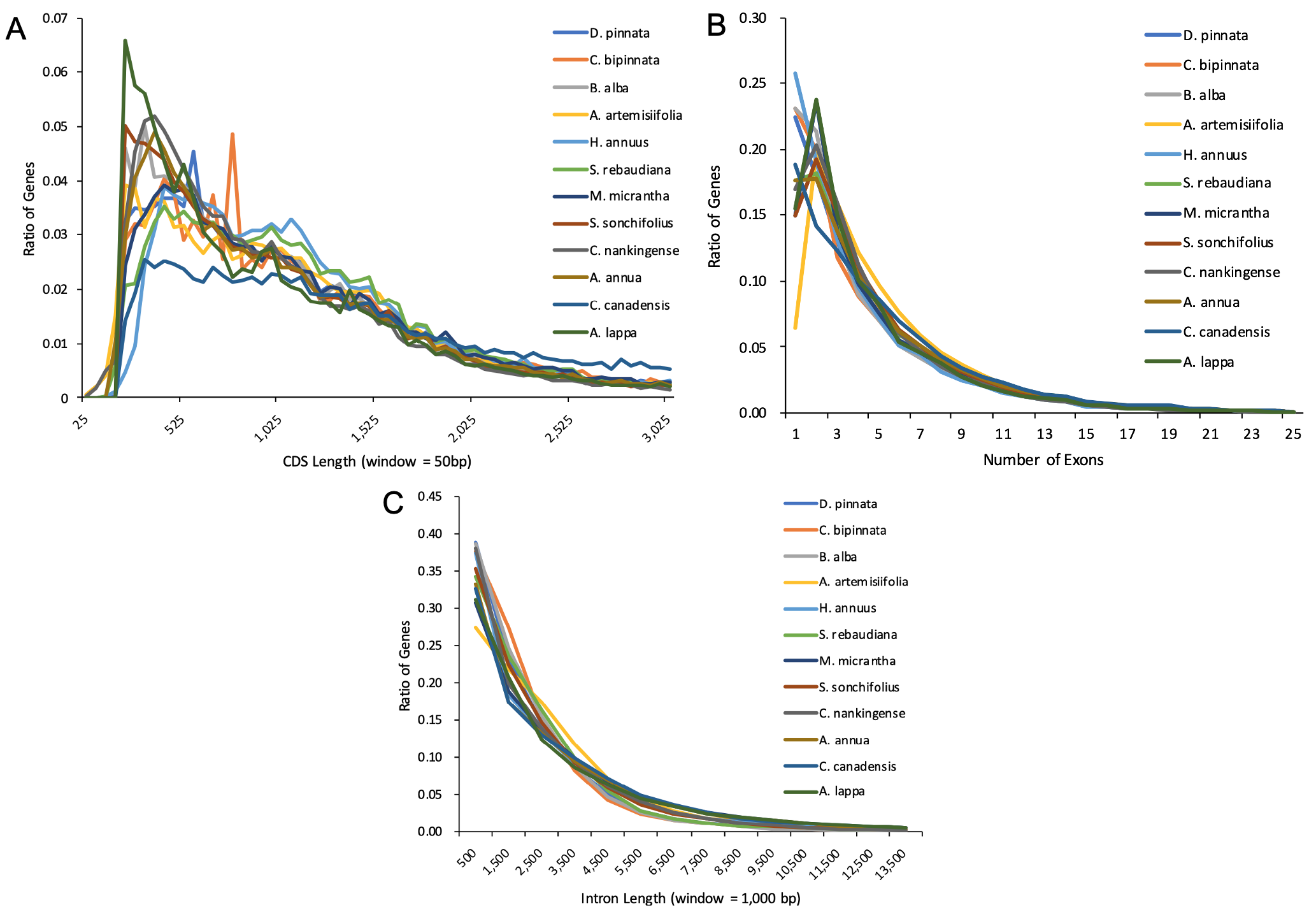


Figure S4. Comparison of gene characteristics among closely related Asteraceae genomes. CDS stands for coding sequences. Distribution of CDS length (A), number of exons (B) and intron length (C) among the gene sets of these plants.


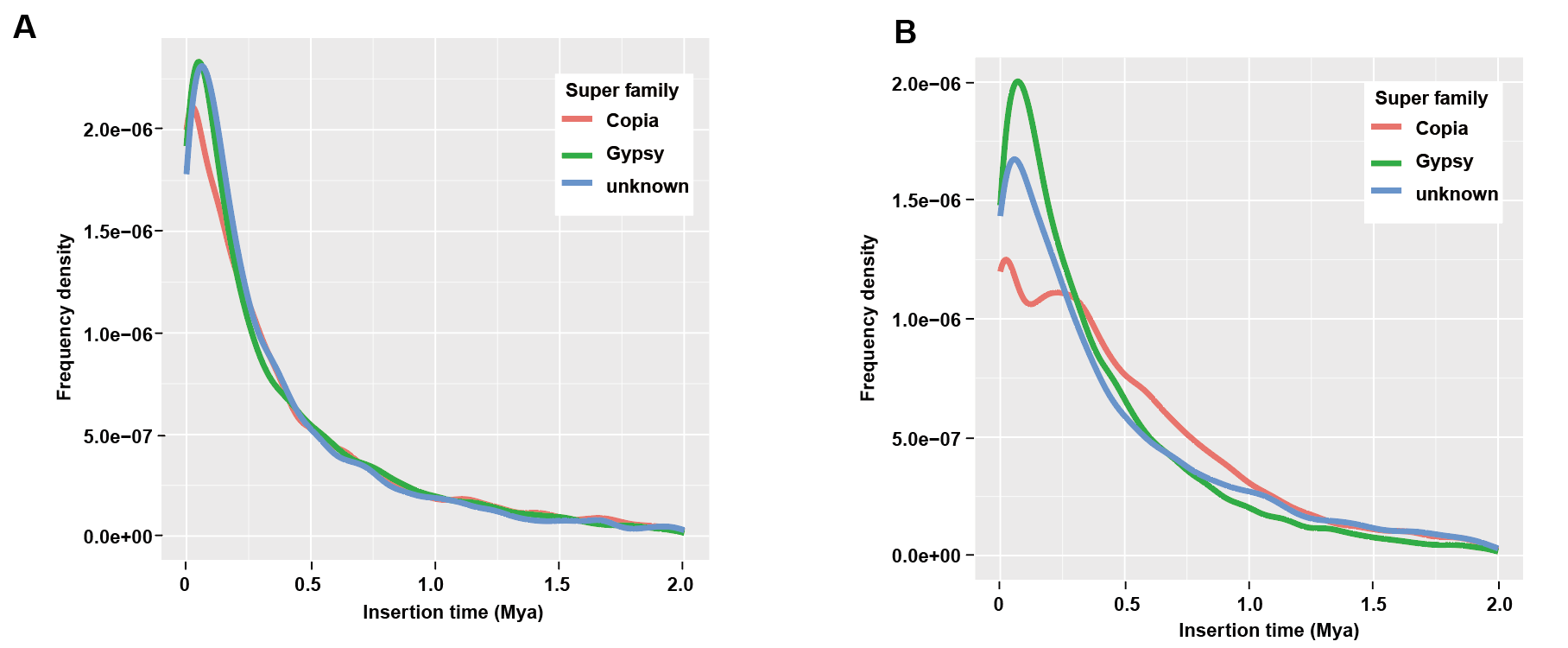


Figure S5. The estimated insertion time of different types of long terminal repeats (LTR) in *C. bipinnatus* (A) and *B. alba* (B). The sequence divergence of long terminal repeats of each LTR and the mutation rate of sunflower were used to estimate the age of LTR.


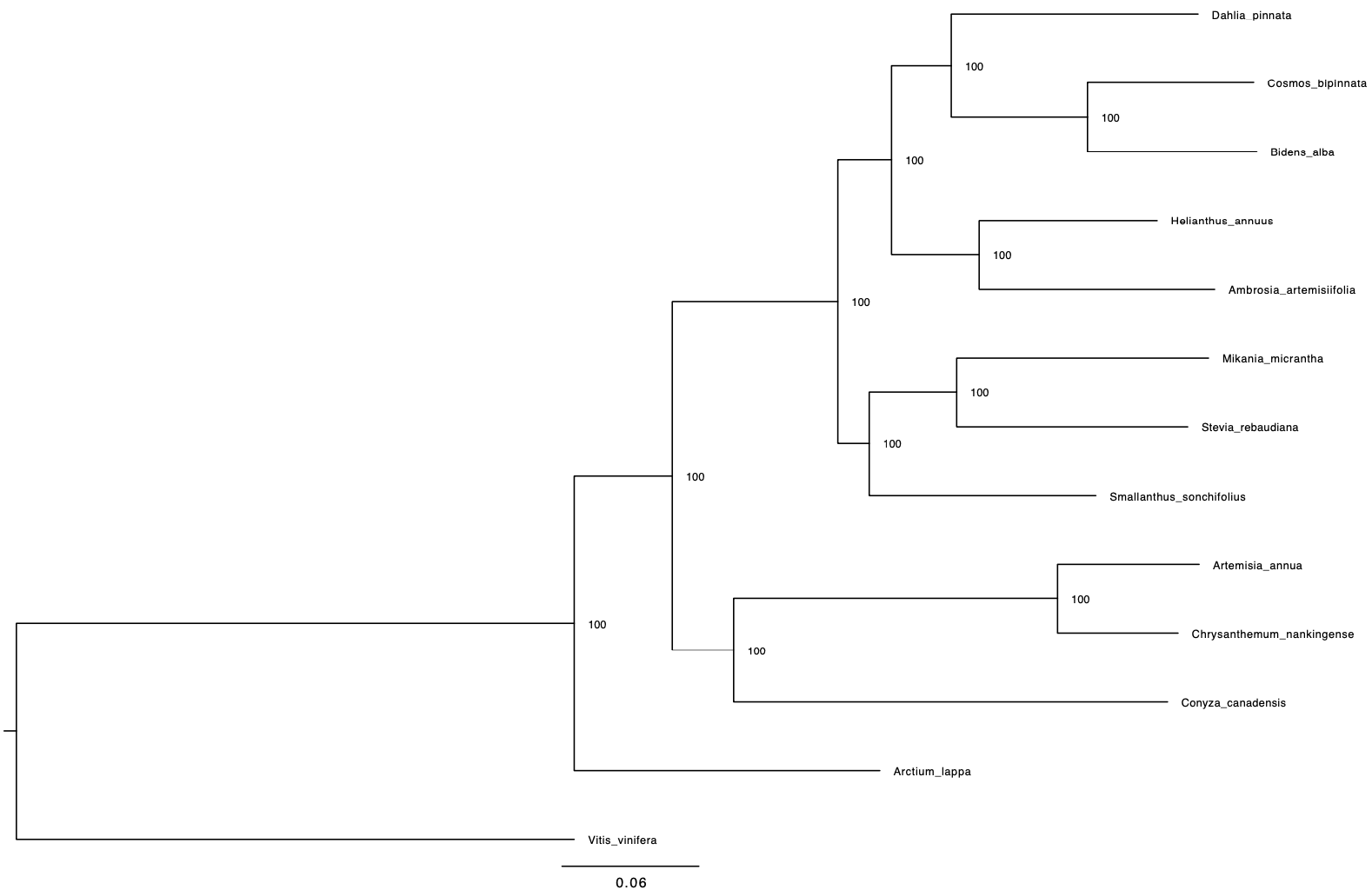


Figure S6. Species tree built by OrthoFinder. This tree have same topology and very similar branch length as tree of Figure 3A. Numbers on the nodes are bootstrap support values. For tetraploid species *D. pinnata* and *B. alba*, genes of two haplotypes (A1 and A3 for *D. pinnata*; A1 and B1 for *B. alba*) were used. *Vitis vinifera* was used as outgroup species.


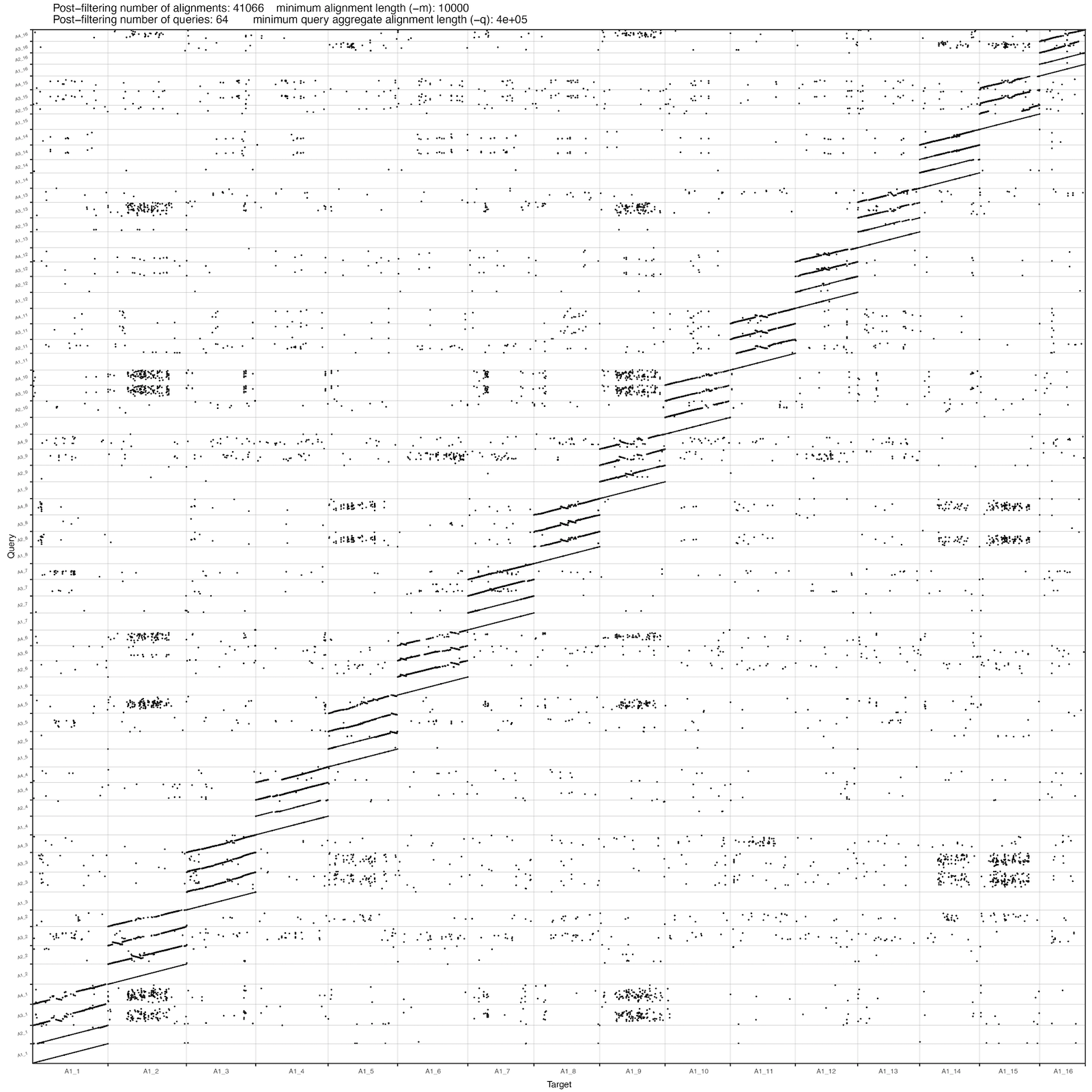


Figure S7. Dot plot of chromosomes alignment for *D. pinnata*. We used minimap2 v2.24 to align all homologous chromosomes with chromosomes from haplotype A1 with parameters “-I 16G -cx asm5” and dotPlotly (https://github.com/tpoorten/dotPlotly) with parameters “-l -x” to draw the figure.


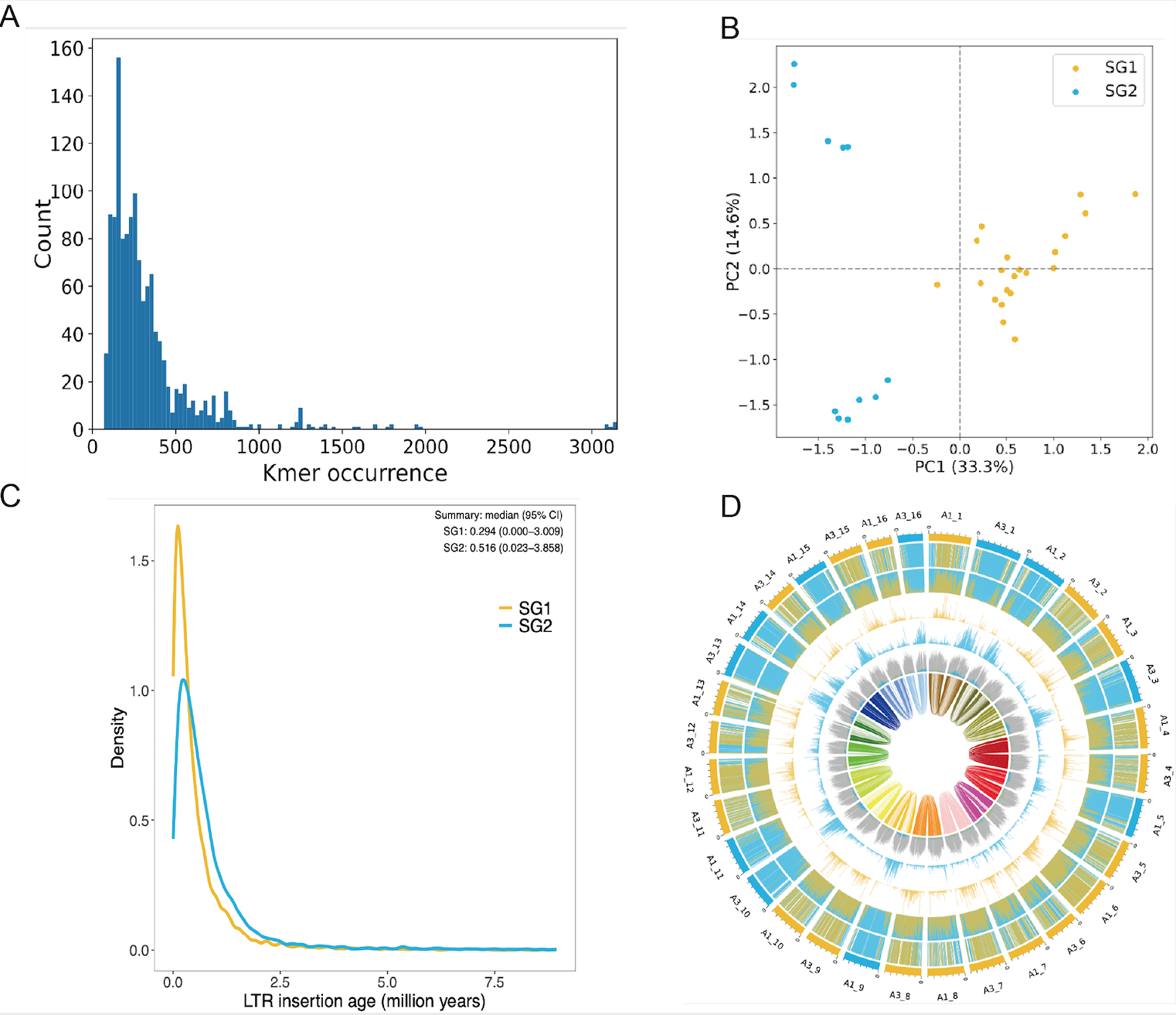


Figure S8. Subgenome (SG) phasing of the *D. pinnata* genome by SubPhaser. (A) Number of differential 15-mers among the homoeologous chromosomes. (B) Principal component analysis (PCA) of differential 15-mers validates that the genome cannot be phased into two subgenomes. (C) Insertion time of subgenome-specific LTR-RTs. The 95% confidence interval (CI) is marked in the upper right corner to predict the insertion time boundaries of LTR-RTs on the subgenome. (D) Chromosomal characteristics. From outer to inner circles (1–7): (1) subgenome assignments based on k-means algorithm; (2) significant enrichment of subgenome-specific k-mers – the same color as the subgenome indicates significant enrichment for those subgenome-specific k-mers; white areas are not significantly enriched; (3) normalized proportion (relative) of subgenome-specific k-mers; (4–5) count (absolute) of each subgenome- specific k-mer set; (6) density of long terminal repeat retrotransposons (LTR-RTs) – if the color is consistent with the subgenome, it indicates that LTR-RTs are significantly enriched to those subgenome-specific k-mers; gray indicates nonspecific LTR-RTs; (7) homoeologous blocks. All statistics (2–6) are computed in sliding windows of 1 Mb. We used haplotypes A1 and A3 for this analysis.


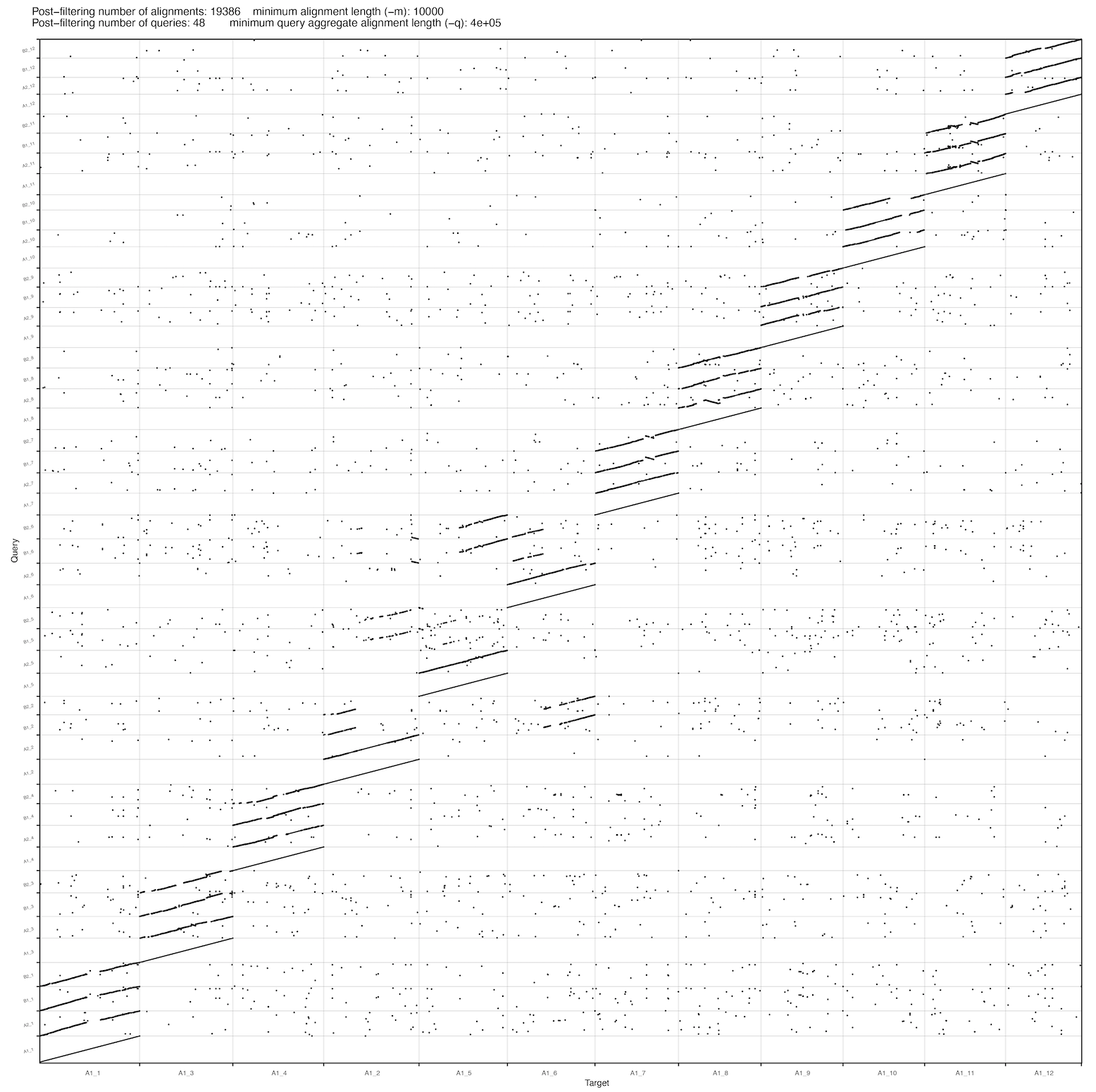


Figure S9. Dot plot of chromosomes alignment for *B. alba*. We used minimap2 v2.24 to align all homologous chromosomes with chromosomes from haplotype A1 with parameters “-I 16G -cx asm5”and dotPlotly (https://github.com/tpoorten/dotPlotly) with parameters “-l -x” to draw the figure. Homologous chromosomes groups Chr2, Chr5 and Chr6 have some crossovers between each other.


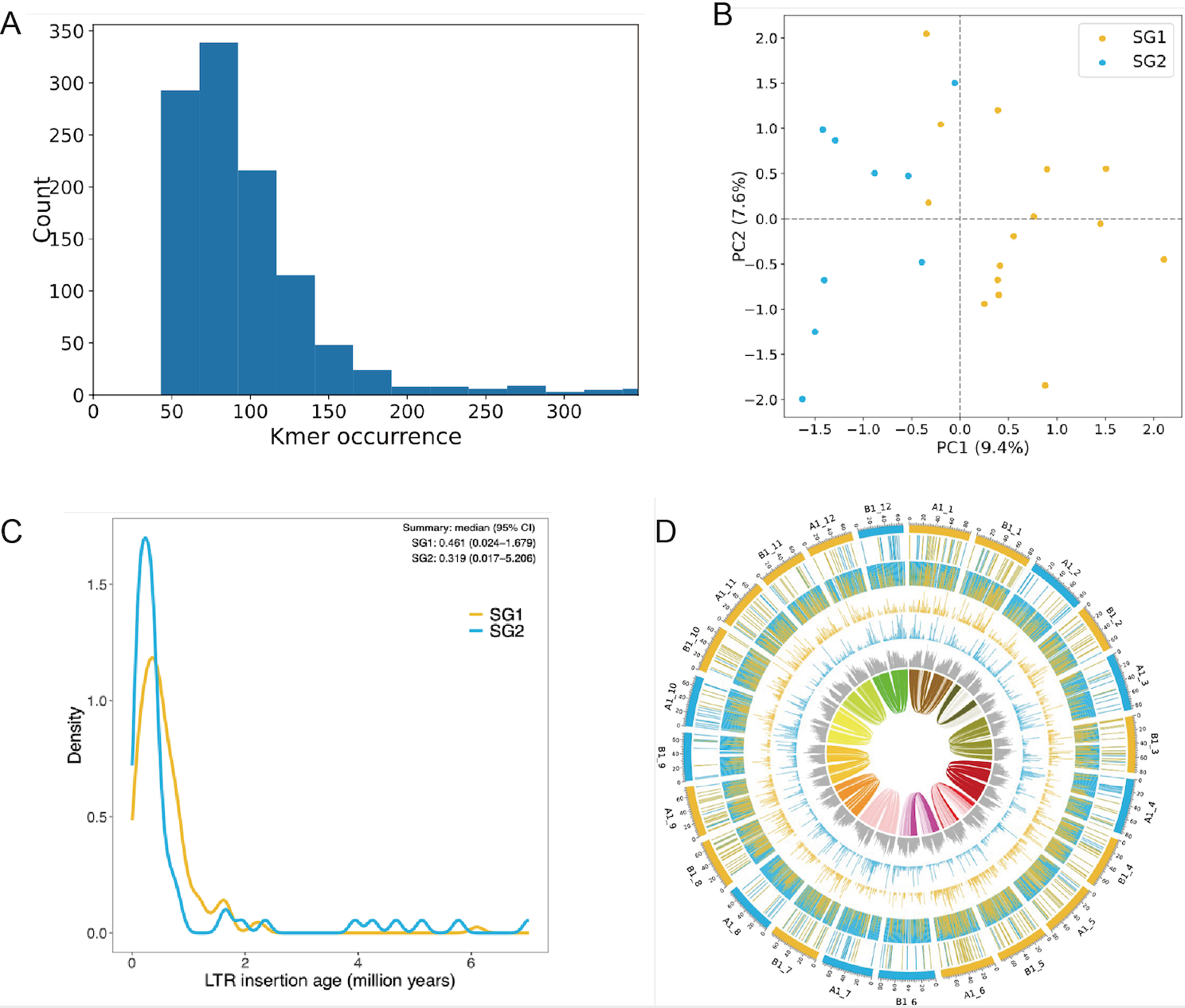


Figure S10. Subgenome (SG) phasing of the *B. alba* genome by SubPhaser. (A) Number of differential 15-mers among the homoeologous chromosomes. (B) Principal component analysis (PCA) of differential 15-mers validates that the genome cannot be phased into two subgenomes. (C) Insertion time of subgenome-specific LTR-RTs. The 95% confidence interval (CI) is marked in the upper right corner to predict the insertion time boundaries of LTR-RTs on the subgenome. (D) Chromosomal characteristics. From outer to inner circles (1–7): (1) subgenome assignments based on k-means algorithm; (2) significant enrichment of subgenome-specific k-mers – the same color as the subgenome indicates significant enrichment for those subgenome-specific k-mers; white areas are not significantly enriched; (3) normalized proportion (relative) of subgenome-specific k-mers; (4–5) count (absolute) of each subgenome- specific k-mer set; (6) density of long terminal repeat retrotransposons (LTR-RTs) – if the color is consistent with the subgenome, it indicates that LTR-RTs are significantly enriched to those subgenome-specific k-mers; gray indicates nonspecific LTR-RTs; (7) homoeologous blocks. All statistics (2–6) are computed in sliding windows of 1 Mb. We used haplotypes A1 and B1 for this analysis.


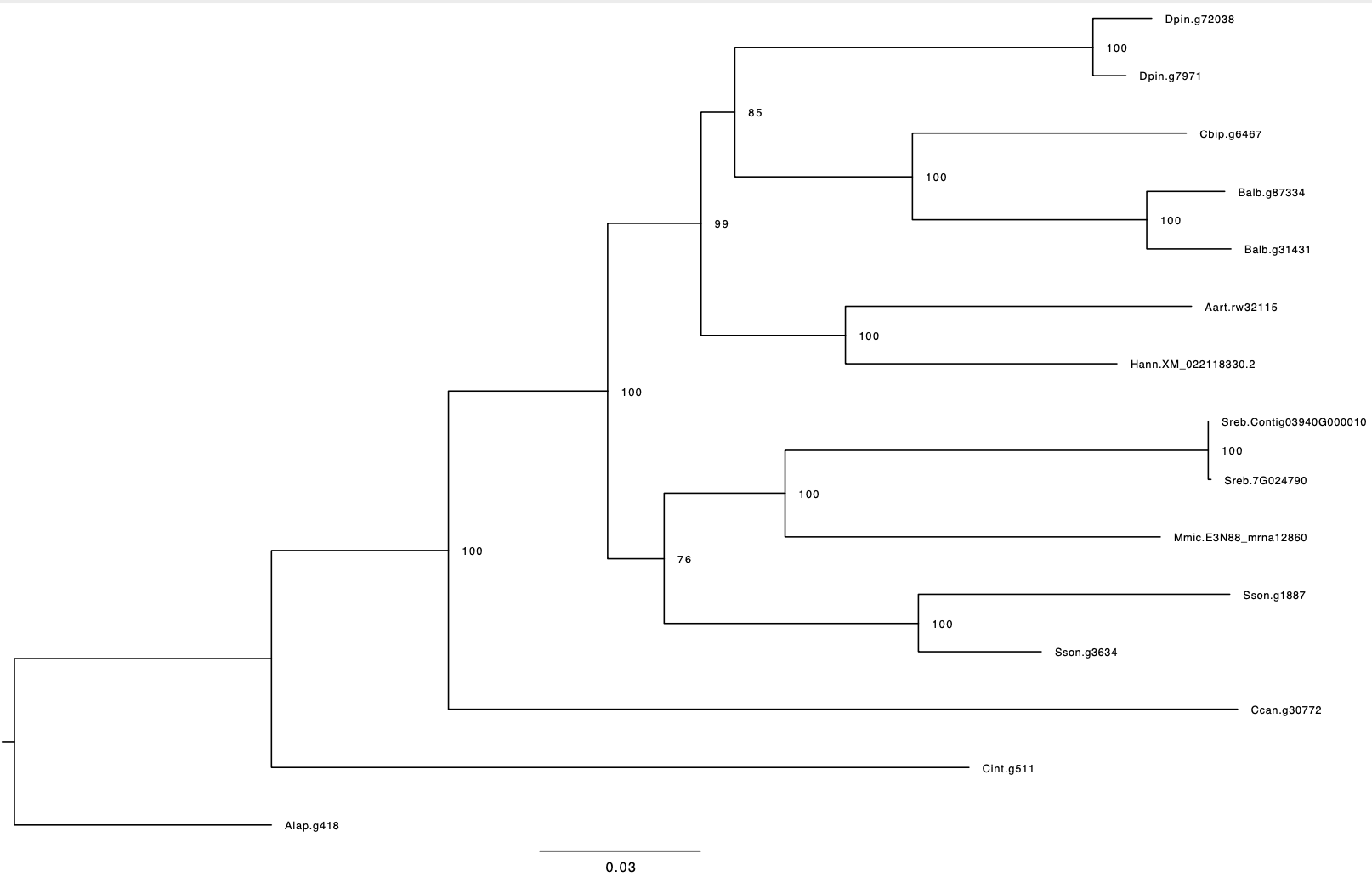


*1-SST*


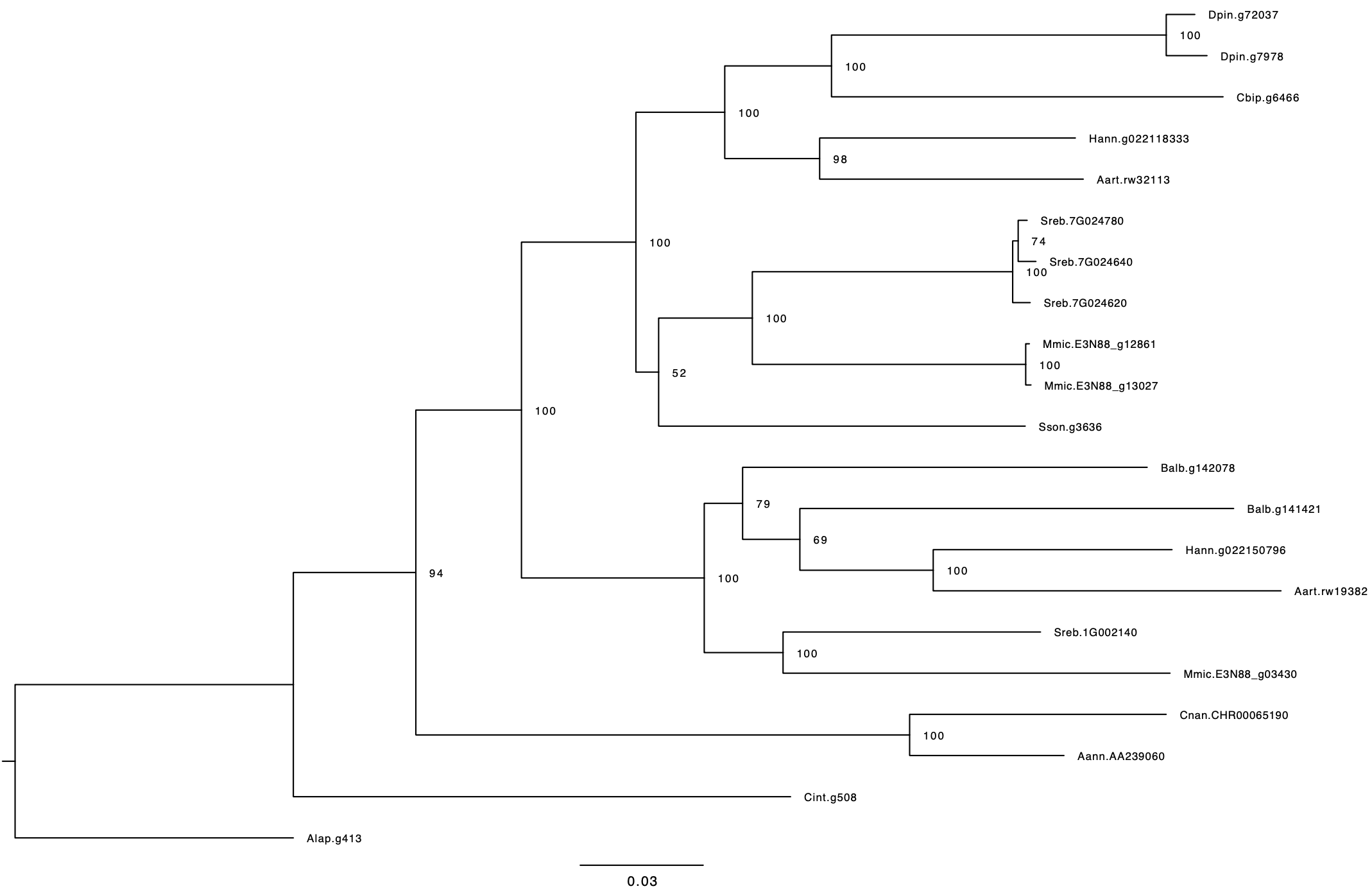


*1-FFT*


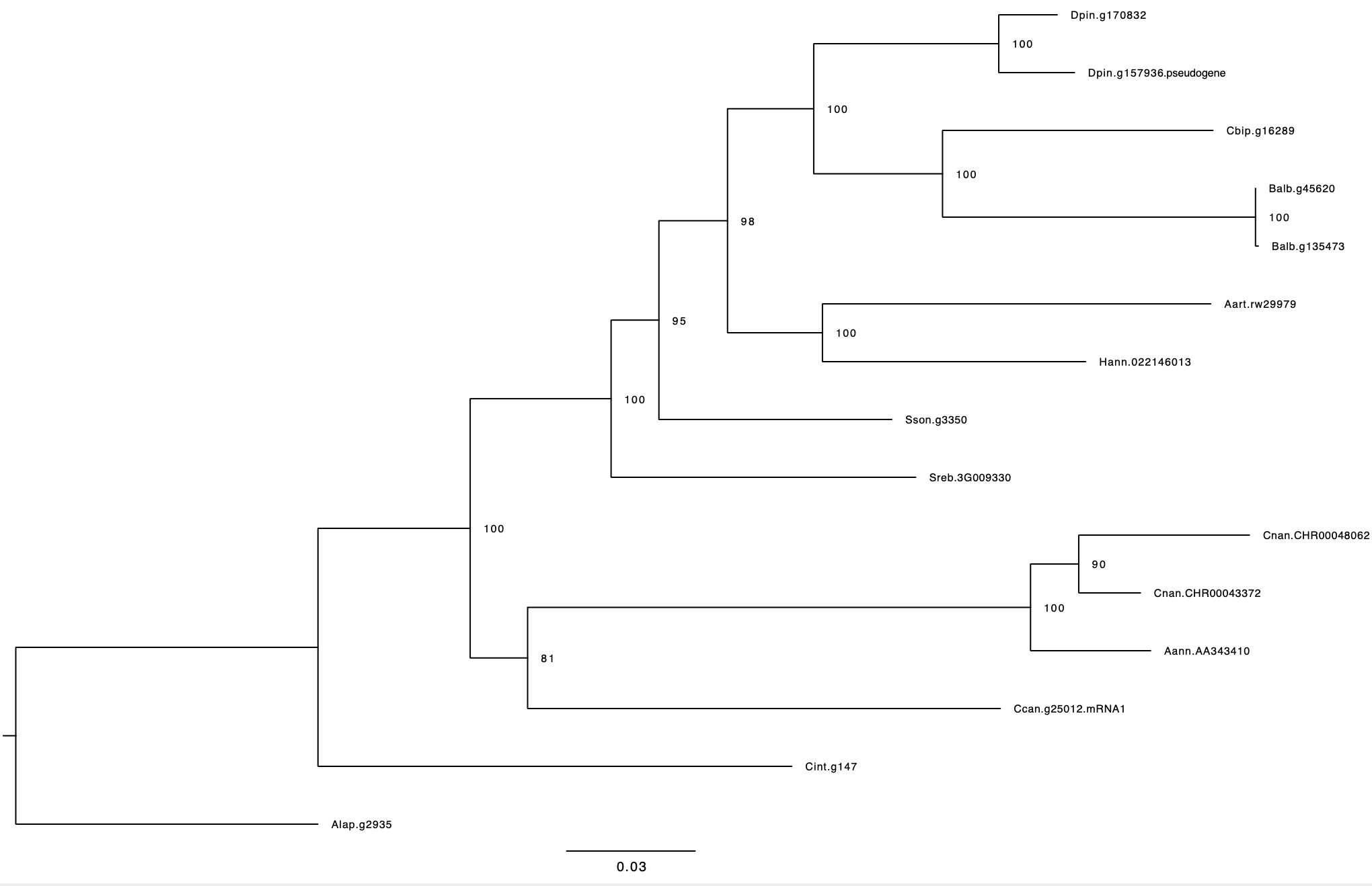


*1-FEH1*


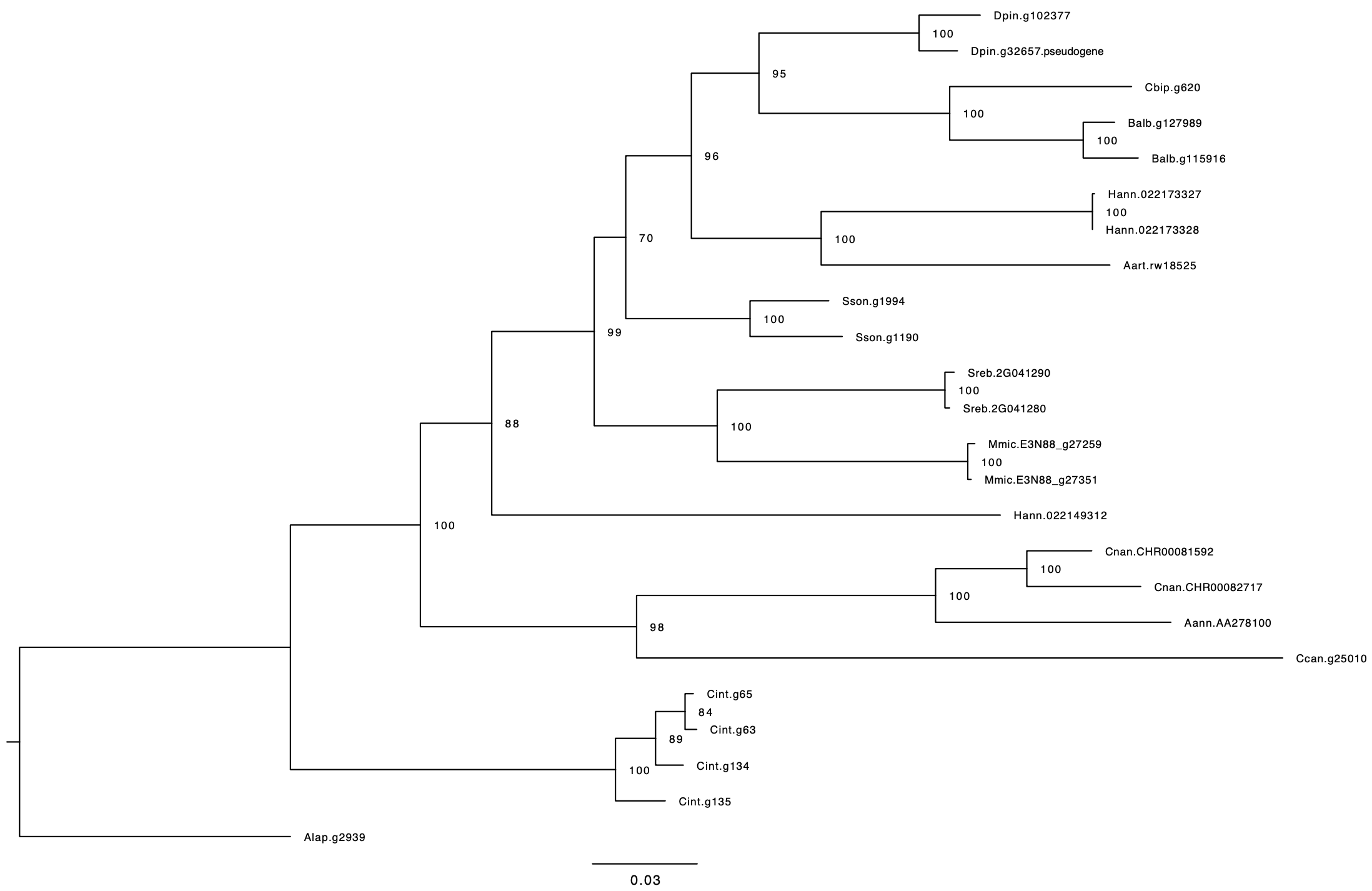


*1-FEH2*


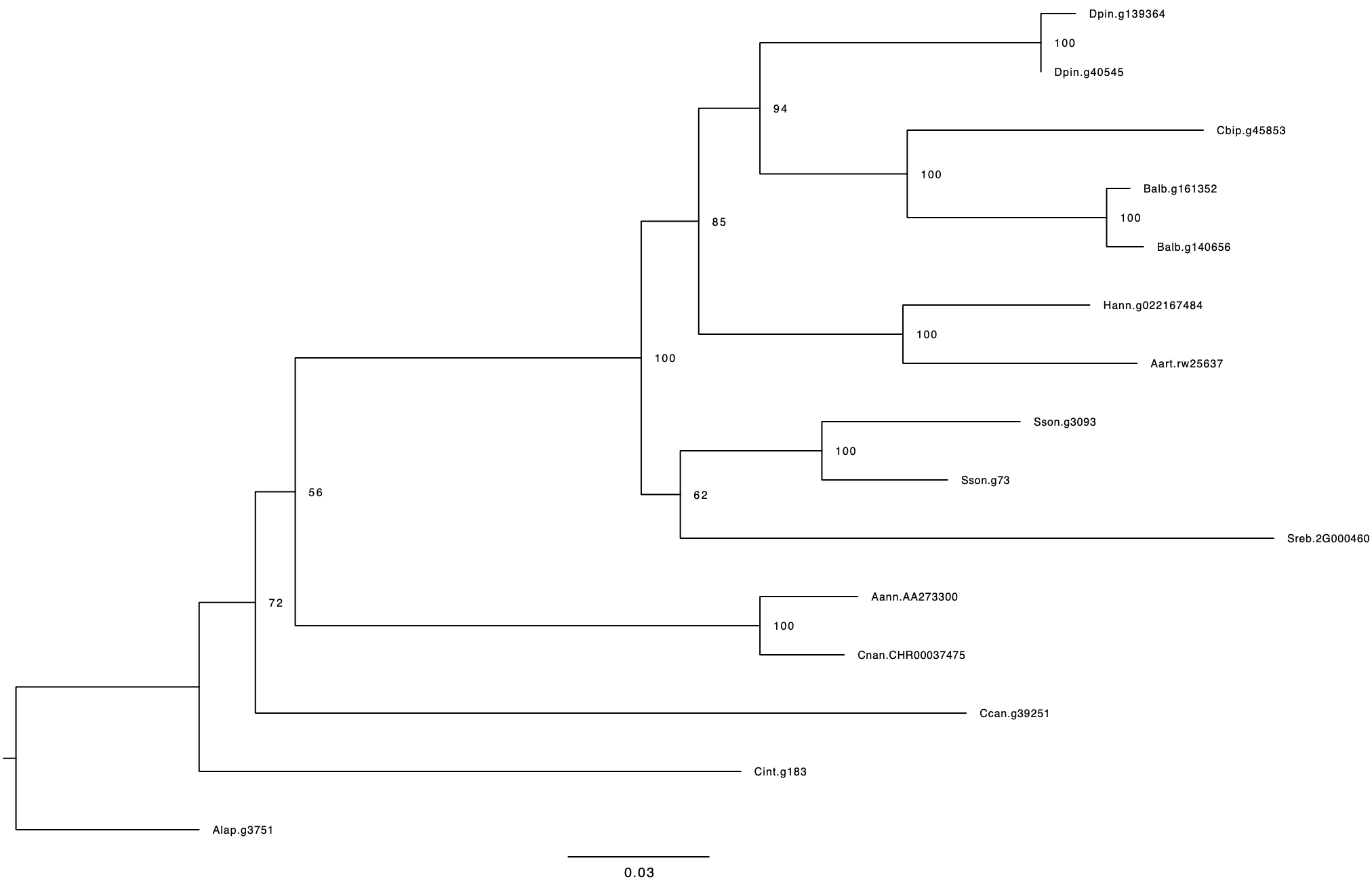


*MYB3*


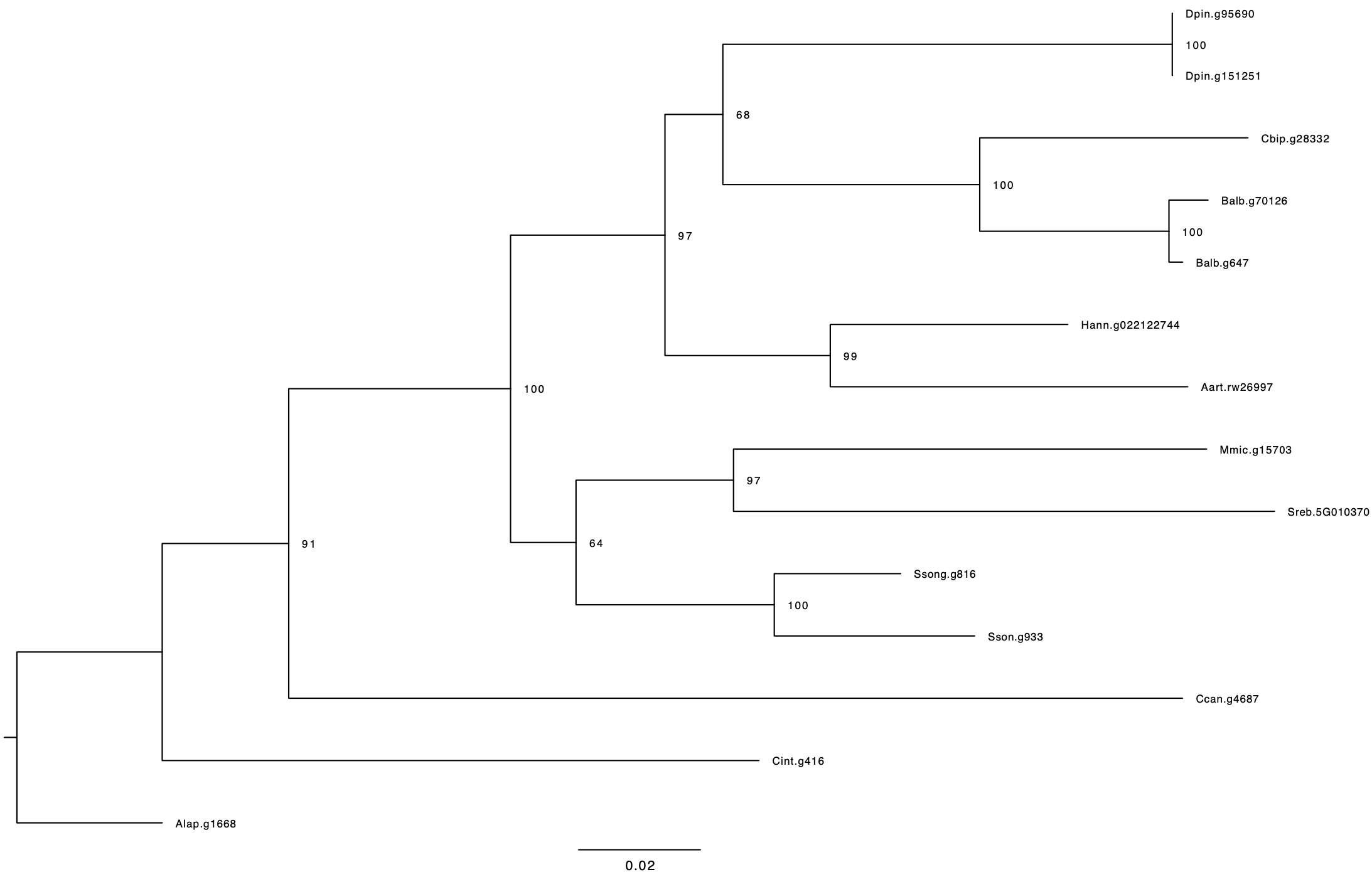


*MYB5*


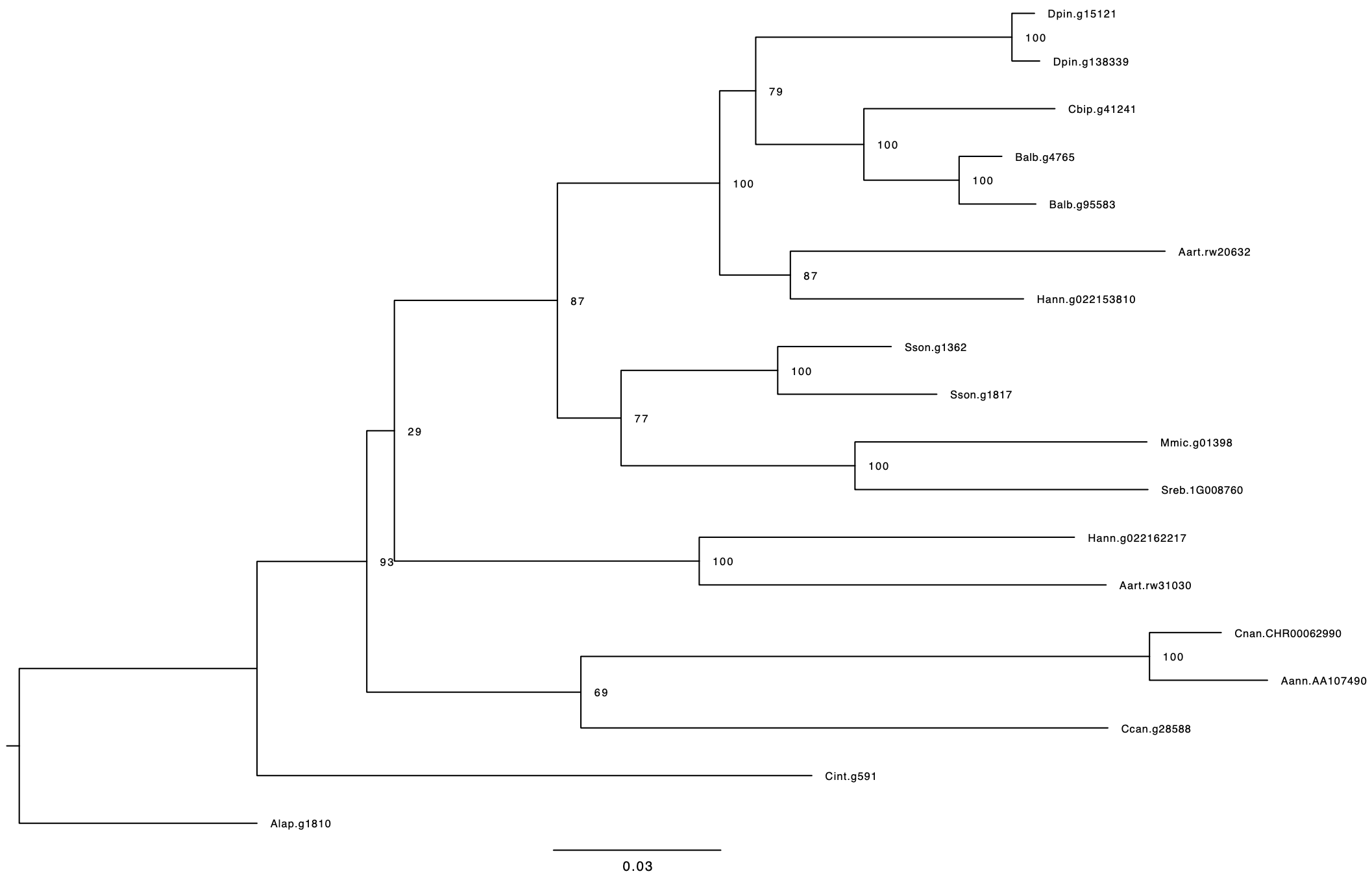


*MYB17*

Figure S11. Gene tree constructed of inulin genes and transcription factor genes. The first letter of the gene id represents for genus name, and the following 3 letters represent for species name. For example, Cint stands for *Cichorium intybus*. The members for each gene families were clustered by OrthoFinder, and the tree figures were plotted by RAxML-NG.


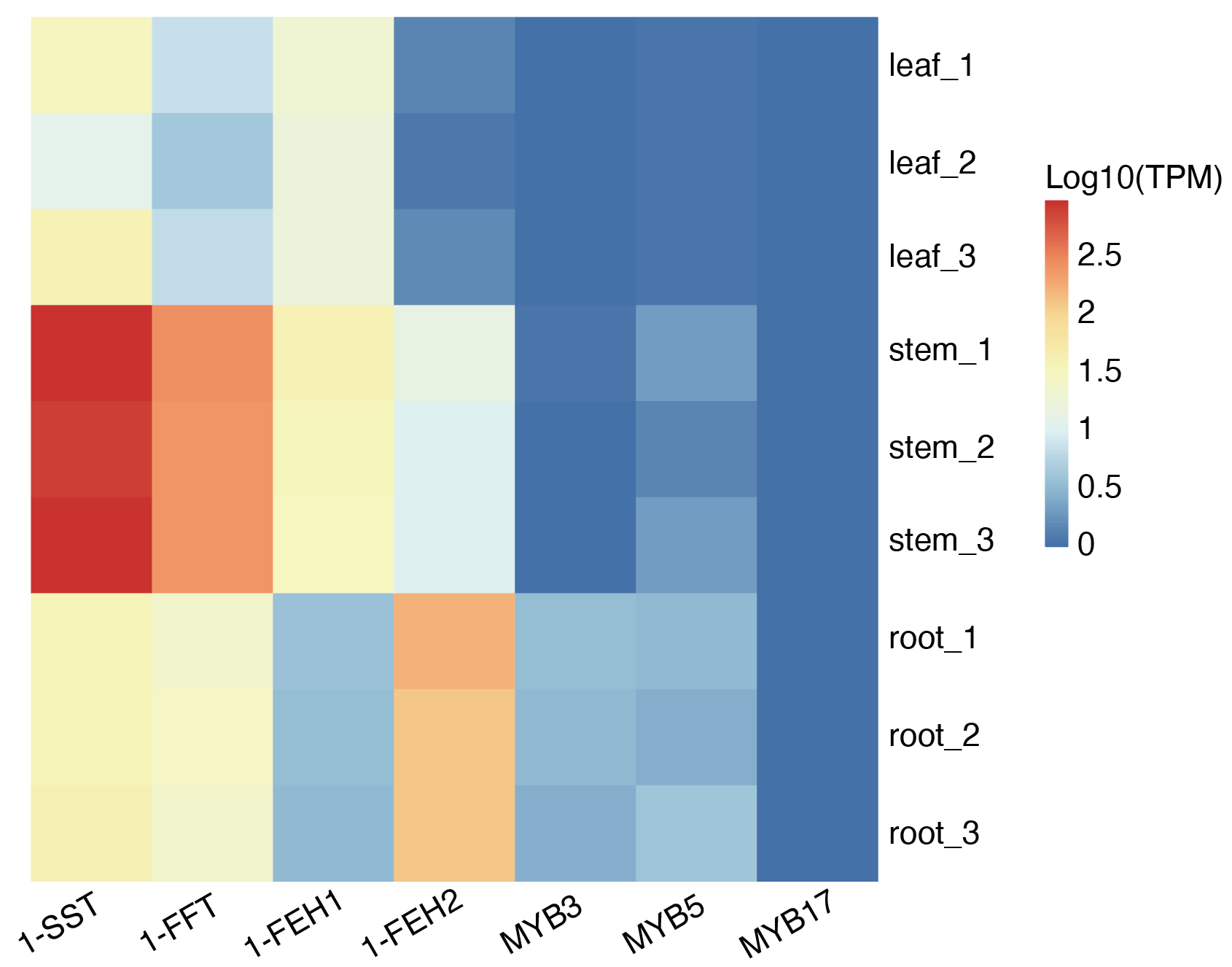


Figure S12. Heatmap of inulin metabolism genes and transcription factor genes in *D. pinnata*. For genes with multiple copies, the sum of expression was demostrated. RNA-seq dataset used for this analysis was downloaded from NCBI (BioProject: PRJNA811758).


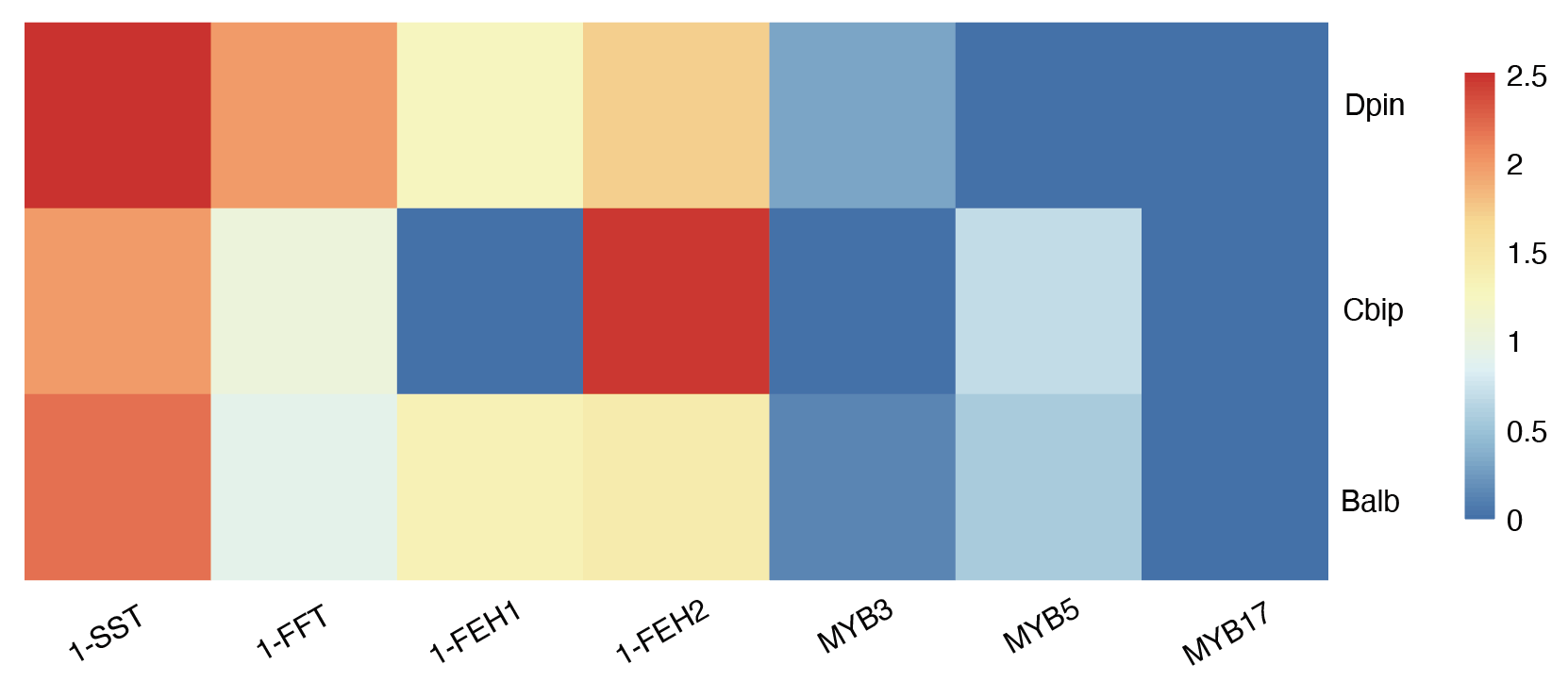


Figure S13. Heatmap of inulin metabolism genes and transcription factor genes in *D. pinnata*, *C. bipinnatus* and *B. alba*. For genes with multiple copies, the sum of expression was demostrated. RNA-seq dataset used for this analysis was downloaded from NCBI (BioProject: PRJNA811758). The full length transcriptomes sequenced by this study were used for gene expression calculation in *C. bipinnatus* and *B. alba*.


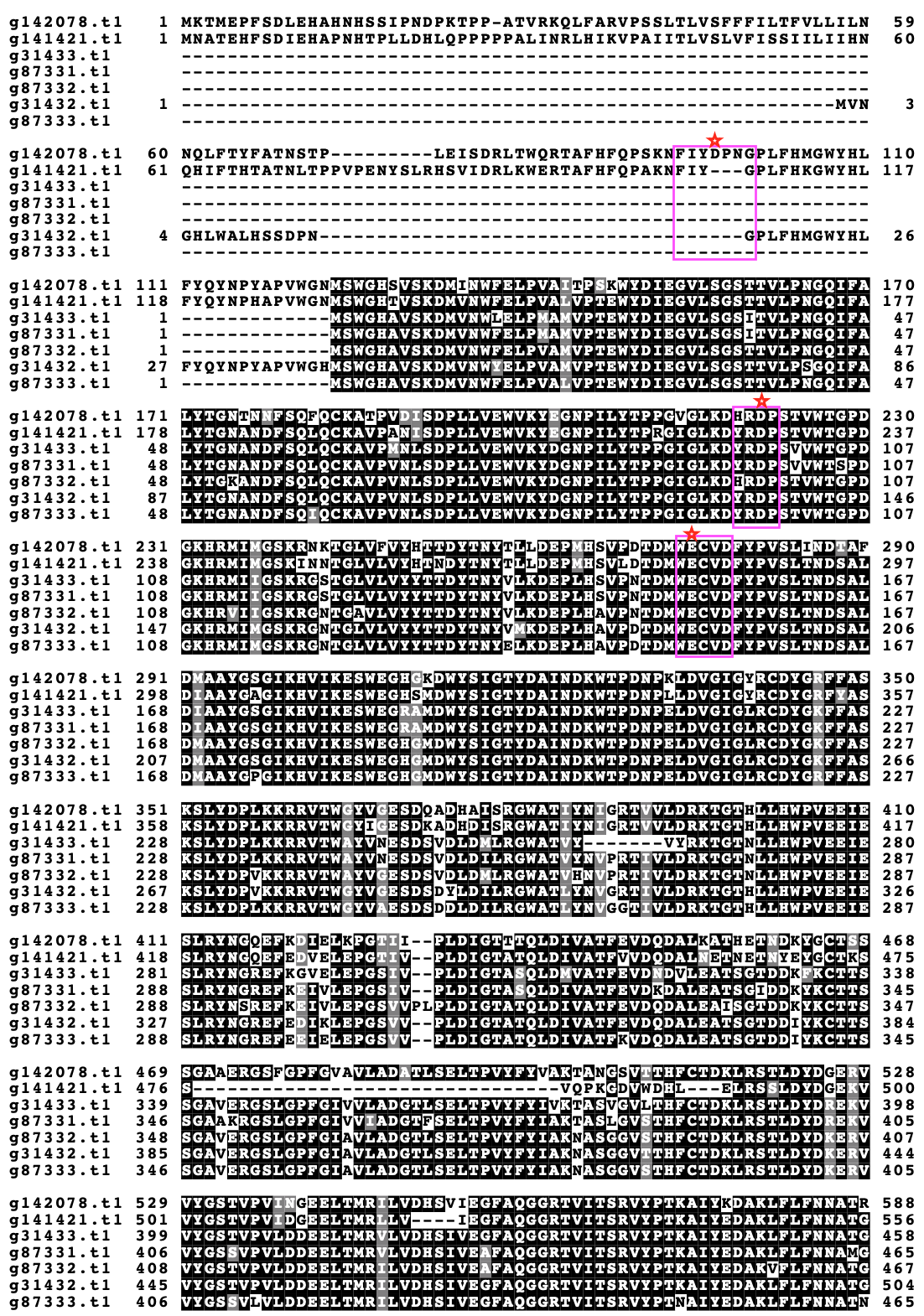


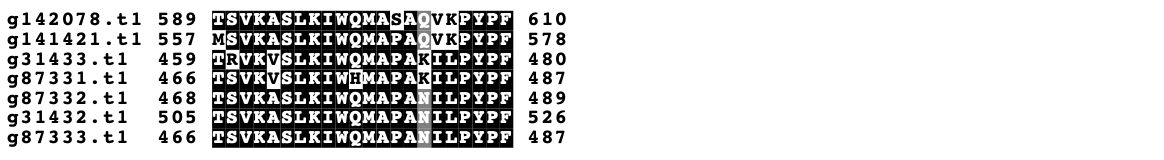


Figure S14. Multiple sequence alignment of 1-FFT genes (*g142078.t1* and *g141421.t1*) and pseudogenes (*g31433.t1*, *g31432.t1*, *g87332.t1*, *g87331.t1* and *g87333.t1*) of *B. alba*. The catalytic triads were marked with pink rectangles and the active sites were marked with red stars. Pseudogenes all lost the first of the catalytic triads.


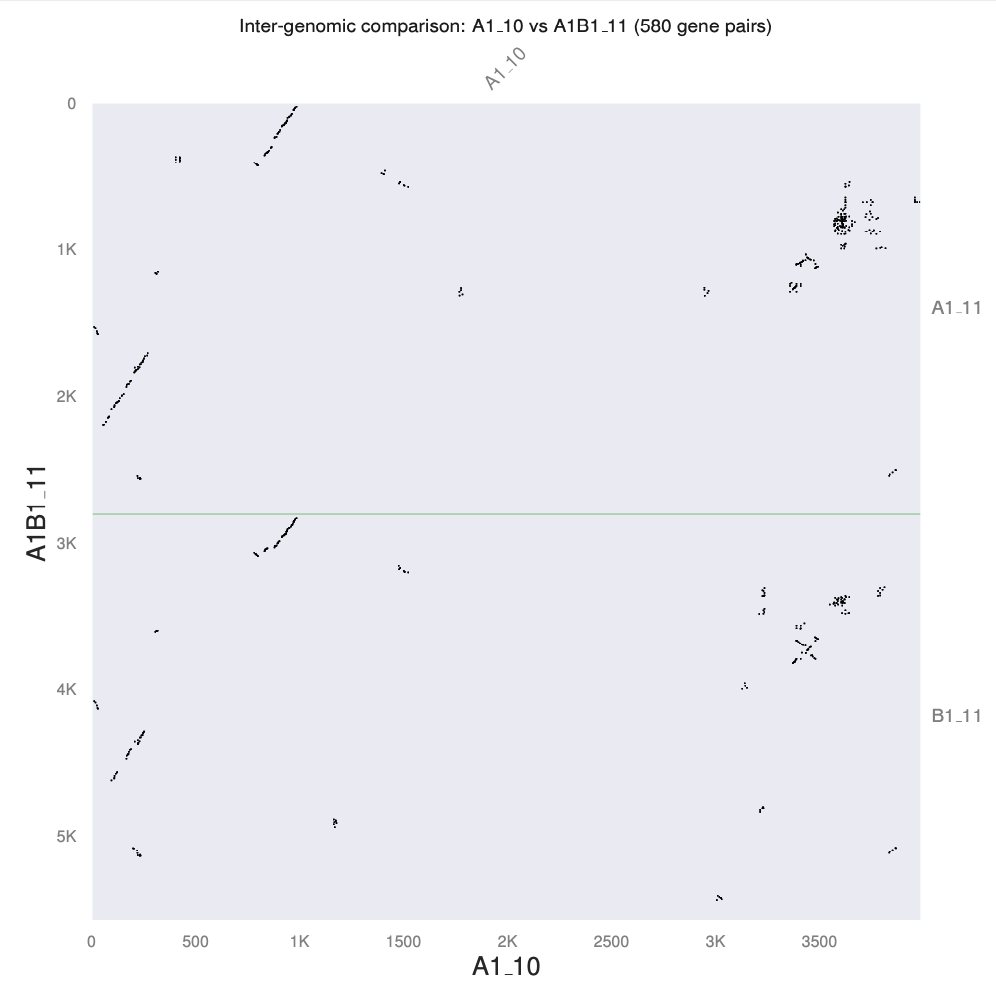


Figure S15. Dot plot of synteny gene blocks between A1_10 and A1_11, B1_11 in *B. alba*. This plot was drawn by jcvi. Clear synteny blocks marked with red circles contained 1-SST and 1-FFT genes. The synteny blocks from different chromosomes indicated that these blocks were originated from ancient whole genome duplications.


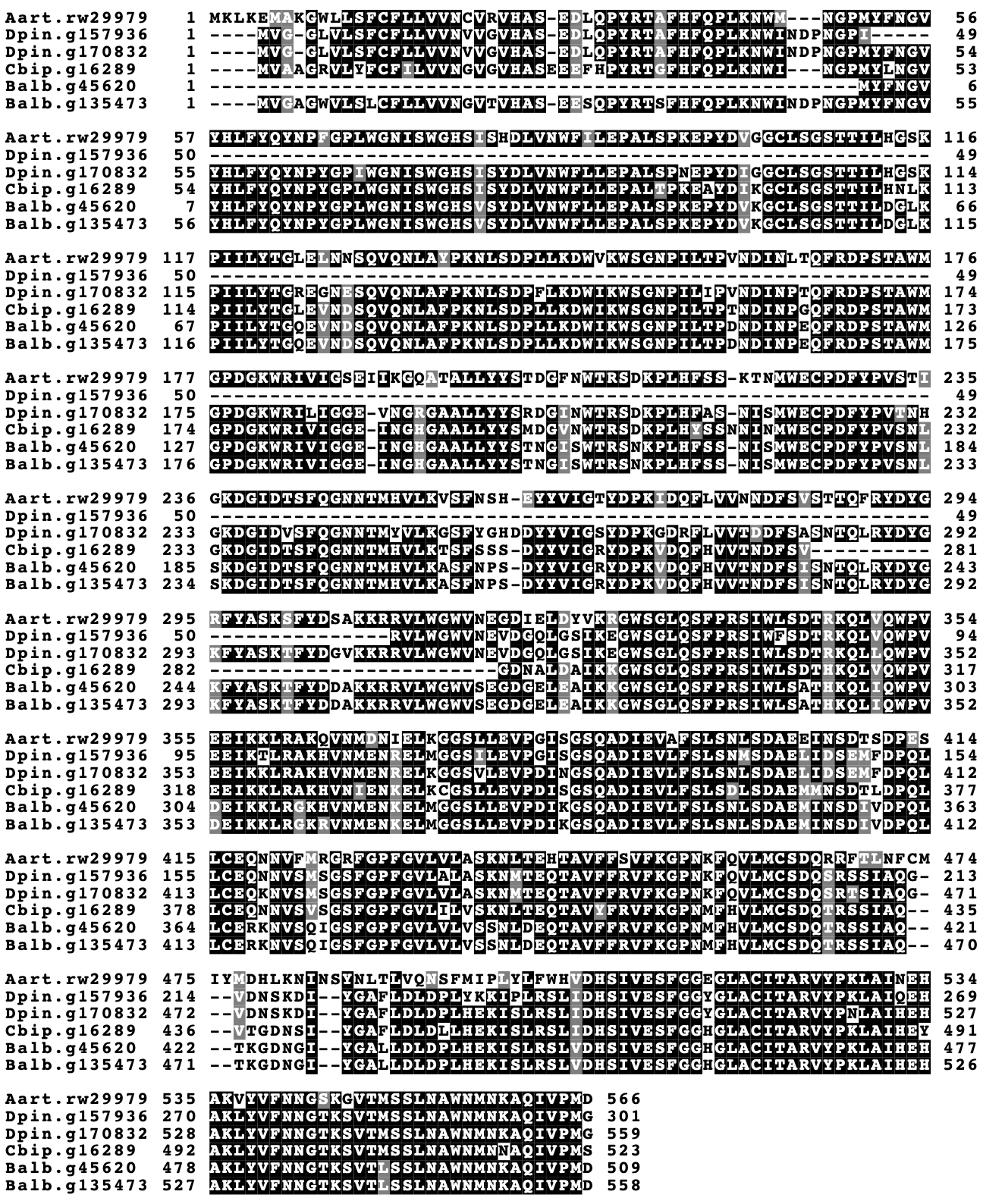


Figure S16. Multiple sequence alignment of 1-FEH1 for closely related species. For sequences compared here, Dpin.g157936 was much shorter than others, and thus, became a pseudogene. This figure corresponding to Figure 6C. For the first four characters, Aart, Dpin, Cbip and Balb stands for *A. artemisiifolia*, *D. pinnata*, *C. bipinnatus* and *B. alba*, respectively.


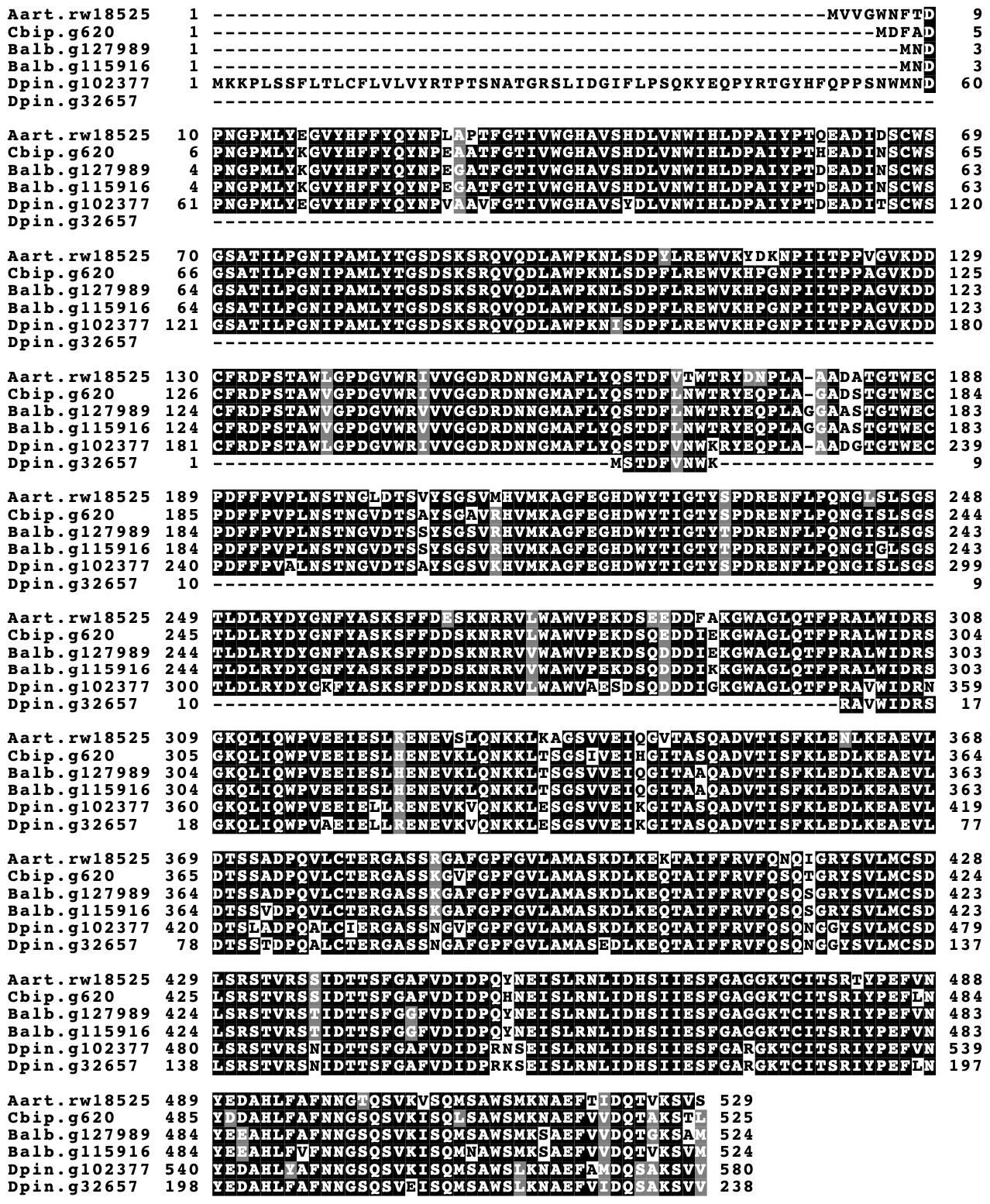


Figure S17. Multiple sequence alignment of 1-FEH2 for closely related species. For sequences compared here, Dpin.g32657 was much shorter than others, and thus, became a pseudogene. This figure corresponding to Figure 6D. For the first four characters, Aart, Dpin, Cbip and Balb stands for *A. artemisiifolia*, *D. pinnata*, *C. bipinnatus* and *B. alba*, respectively.


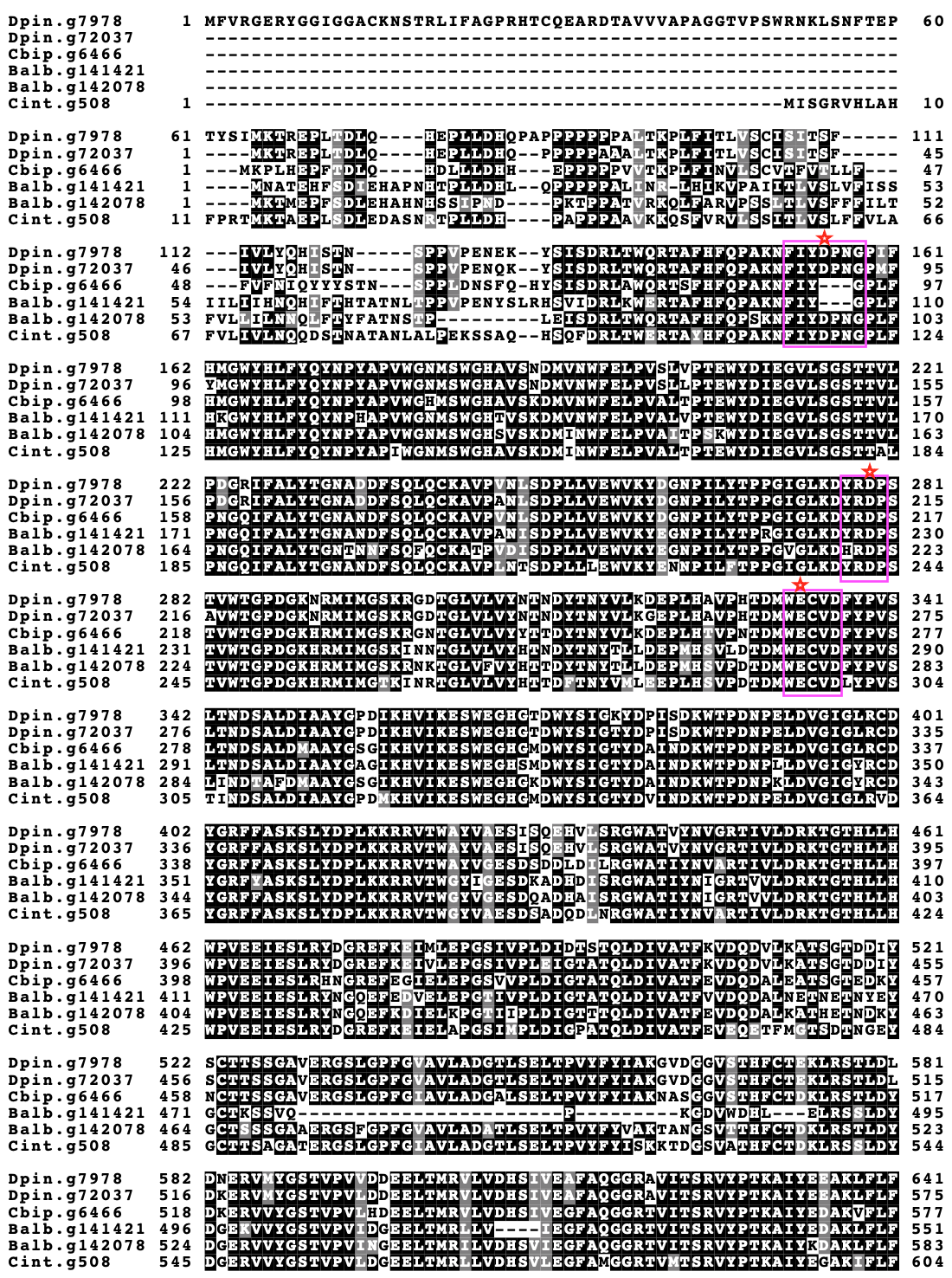


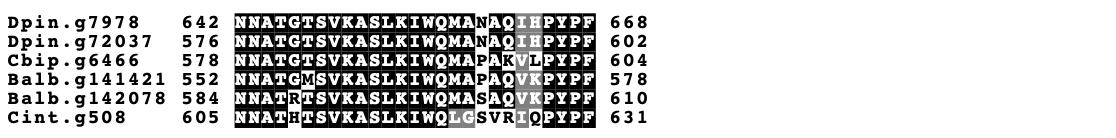


Figure S18. Multiple sequence alignment of 1-FFT genes of *D. pinnata*, *C. bipinnatus*, *B. alba* and *Cichorium intybus*. The catalytic triads were marked with pink rectangles and the active sites were marked with red stars. pyBoxshade (https://github.com/mdbaron42/pyBoxshade) was used to show this alignment.

**Supplementary Tables**

Table S1. Sequencing statistics of *D. pinnata*, *B. alba* and *C. bipinnatus*.

| **Species** | **Source** | **Libraries** | **Sequence Type** | **Insert Size** | **N50 Read Length** | **Total Raw Data (Gb)** | **NCBI Accession** | **NGDC Accession** |
| --- | --- | --- | --- | --- | --- | --- | --- | --- |
| *D. pinnata* | Genomic DNA | DNBSEQ | PE | 500 bp | 150 bp | 240.1 | SRR27335570-SRR27335573 | CRA011358 |
|  |  | Illumina Hi-C | PE | 500 bp | 150 bp | 368.0 | SRR27309800- SRR27309803 | CRA011357 |
|  |  | PacBio-HiFi | SE | 20 kb | 19.1 kb | 192.5 | SRR27324941-SRR27324946 | CRA011354 |
|  | Transcriptome | PacBio Iso | SE | 0.5 ~ 6 kb | 2.4 kb | 0.09 | SRR27322315 | CRA011360 |
| *B. alba* | Genomic DNA | Illumina Hi-C | PE | 500 bp | 150 bp | 163.1 | SRR27325088 | CRA011457 |
|  |  | PacBio-HiFi | SE | 20 kb | 18.0 kb | 93.2 | SRR27325027-SRR27325030 | CRA011468 |
|  | Transcriptome | PacBio Iso | SE | 0.5 ~ 6 kb | 2.9 kb | 0.4 | SRR27322717 | CRA011423 |
| *C. bipinnatus* | Genomic DNA | Illumina Hi-C | PE | 500 bp | 150 bp | 113.7 | SRR27331023-SRR27331024 | CRA011502 |
|  |  | PacBio-HiFi | SE | 20 kb | 24.1 kb | 78.0 | SRR27331500-SRR27331501 | CRA011472 |
|  | Transcriptome | PacBio Iso | SE | 0.5 ~ 6 kb | 1.8 kb | 0.04 | SRR27325017 | CRA011477 |

Note: SE stands for single end; PE stands for pair ends.

Table S2. Statistics of reference organelle genomes used for filtering sequence from organelle.

| **Organelle genomes** | **Length (bp)** | **NCBI accession** |
| --- | --- | --- |
| *Bidens pilosa* chloroplast | 151,496 | MW551953.1 |
| *Bidens pilosa* chloroplast | 154,478 | MZ127828.1 |
| *Bidens alba var. radiata* chloroplast | 151,635 | MW551955.1 |
| *Bidens bipinnata* mitochondrion | 198,476 | MW838189.1 |
| *Bidens alba var. radiata* mitochondrion | 213,288 | MW838194.1 |
| *Bidens parviflora* mitochondrion | 195,825 | MW838187.1 |
| *Cosmos bipinnatus* chloroplast | 150,356 | MN518845.1 |
| *Dahlia pinnata* chloroplast | 152,111 | MW900255.1 |
| *Dahlia pinnata* chloroplast | 152,113 | MW900253.1 |
| *Dahlia pinnata* chloroplast | 152,169 | MW900254.1 |
| *Dahlia pinnata* chloroplast | 152,107 | OP006582.1 |

Table S3. Statistics of filtered organelle genome sequence.

|  | ***D. pinnata*** | ***C. bipinnatus*** | ***B. alba*** |
| --- | --- | --- | --- |
| Contig numbers | 105 | 9 | 222 |
| Total length (bp) | 4,065,944 | 537,430 | 8,742,688 |
| Longest contig (bp) | 65,422 | 80,311 | 174,549 |
| N50 length (bp) | 40,674 | 62,900 | 40,422 |
| Shortest contig (bp) | 17,000 | 38,408 | 12,806 |

Table S4. Statistics of contig assemblies for *D. pinnata*, *C. bipinnatus* and *B. alba*.

|  | ***D. pinnata*** | | ***C. bipinnatus*** | | ***B. alba*** | |
| --- | --- | --- | --- | --- | --- | --- |
| Total | 4,558 | 7,856,742,314 | 487 | 1,015,496,390 | 1,630 | 3,749,031,044 |
| Maximum | 1 | 133,153,355 | 1 | 73,793,381 | 1 | 78,279,585 |
| N10 | 7 | 84,953,643 | 1 | 73,180,627 | 6 | 54,431,575 |
| N20 | 20 | 51,480,525 | 2 | 67,392,268 | 13 | 40,927,695 |
| N30 | 36 | 42,617,399 | 4 | 64,430,975 | 25 | 27,245,258 |
| N40 | 56 | 36,288,185 | 6 | 56,461,933 | 42 | 20,497,737 |
| N50 | 80 | 29,279,538 | 7 | 52,817,601 | 63 | 15,049,174 |
| N60 | 110 | 22,962,136 | 10 | 36,001,967 | 93 | 10,664,802 |
| N70 | 150 | 16,820,250 | 14 | 16,855,226 | 135 | 7,278,202 |
| N80 | 209 | 10,524,969 | 24 | 7,723,573 | 201 | 4,423,644 |
| N90 | 320 | 4,350,380 | 45 | 2,619,544 | 314 | 2,189,951 |
| Minimum | 1 | 11,942 | 1 | 21,926 | 1 | 11,650 |

Table S5. Statistics of Hi-C reads mapping for *D. pinnata*.

|  | R1 | R2 |
| --- | --- | --- |
| total reads | 1,226,633,551 | 1,226,633,551 |
| mapped reads | 1,218,170,010 | 1,215,774,582 |
| % of mapped reads | 99.31% | 99.11% |
| total reads after filtering | 943,760,017 | 1,024,706,741 |
| mapped reads after filtering | 805,194,099 | 945,022,598 |
| % of mapped reads after filtering | 85.32% | 92.22% |
| mapped reads after paired | 2,034,697,808 | |
| % of mapped reads after paired | 82.94% | |
| All | 1,017,348,904 | |
| % of reads in All | 41.47% | |
| All intra | 204,515,058 | |
| All intra 1kb | 197,396,053 | |
| All intra 10kb | 173,115,228 | |
| All intra 15kb | 165,730,855 | |
| All intra 20kb | 160,084,233 | |
| All inter | 812,833,846 | |

Note: Because *D. pinnata* is a tetraploid plant and reads mapping quality will be low for very similar sequences, we set MAPQ_FILTER=0 in the Arima mapping pipeline.

Table S6. Statistics of Hi-C reads mapping for *C. bipinnatus*.

|  | R1 | R2 |
| --- | --- | --- |
| total reads | 378,855,163 | 378,855,163 |
| mapped reads | 375,427,567 | 375,589,738 |
| % of mapped reads | 99.10% | 99.14% |
| total reads after filtering | 323,158,206 | 323,079,581 |
| mapped reads after filtering | 273,582,914 | 272,084,981 |
| % of mapped reads after filtering | 84.66% | 84.22% |
| mapped reads after paired | 164,577,704 | |
| % of mapped reads after paired | 21.72% | |
| All | 80,729,042 | |
| % of reads in All | 10.65% | |
| All intra | 46,919,943 | |
| All intra 1kb | 38,346,253 | |
| All intra 10kb | 31,485,729 | |
| All intra 15kb | 29,886,424 | |
| All intra 20kb | 28,710,249 | |
| All inter | 33,809,099 | |

Table S7. Statistics of Hi-C reads mapping for *B. alba*.

|  | R1 | R2 |
| --- | --- | --- |
| total reads | 543,520,152 | 543,520,152 |
| mapped reads | 541,497,785 | 541,464,679 |
| % of mapped reads | 99.63% | 99.62% |
| total reads after filtering | 462,886,877 | 462,808,618 |
| mapped reads after filtering | 406,034,446 | 405,218,359 |
| % of mapped reads after filtering | 87.72% | 87.56% |
| mapped reads after paired | 866,035,776 | |
| % of mapped reads after paired | 79.67% | |
| All | 427,929,229 | |
| % of reads in All | 39.37% | |
| All intra | 93,824,622 | |
| All intra 1kb | 86,846,210 | |
| All intra 10kb | 74,038,614 | |
| All intra 15kb | 70,667,932 | |
| All intra 20kb | 68,157,066 | |
| All inter | 334,104,607 | |

Note: Because *B. alba* is a tetraploid plant and reads mapping quality will be low for very similar sequences, we set MAPQ_FILTER=0 in the Arima mapping pipeline.

Table S8. Statistics of scaffold assemblies for *D. pinnata*, *C. bipinnatus* and *B. alba*.

|  | ***D. pinnata*** | | ***C. bipinnatus*** | | ***B. alba*** | |
| --- | --- | --- | --- | --- | --- | --- |
| Total | 3,987 | 7,857,313,314 | 407 | 1,015,576,390 | 1,226 | 3,749,125,044 |
| Maximum | 1 | 153,464,679 | 1 | 96,698,554 | 1 | 90,500,070 |
| N10 | 5 | 139,686,935 | 1 | 90,922,703 | 4 | 85,186,813 |
| N20 | 11 | 132,112,987 | 2 | 89,922,060 | 8 | 83,572,548 |
| N30 | 17 | 126,612,967 | 3 | 84,663,035 | 13 | 79,775,746 |
| N40 | 23 | 121,155,482 | 4 | 82,164,069 | 18 | 75,648,341 |
| N50 | 30 | 118,510,872 | 5 | 79,416,621 | 23 | 75,126,418 |
| N60 | 36 | 115,168,395 | 7 | 77,652,268 | 28 | 72,831,775 |
| N70 | 43 | 112,151,027 | 8 | 76,134,569 | 33 | 70,080,681 |
| N80 | 50 | 108,759,612 | 9 | 76,003,301 | 38 | 67,405,598 |
| N90 | 58 | 97,038,195 | 11 | 46,114,586 | 44 | 64,594,447 |
| Minimum | 1 | 11,942 | 1 | 21,926 | 1 | 11,650 |

Table S9. Positions of telomeres on the chromosomes of *D. pinnata*.

| **Chr ID** | **Start position** | **End position** | **Chr ID** | **Start position** | **End position** | **Chr ID** | **Start position** | **End position** |
| --- | --- | --- | --- | --- | --- | --- | --- | --- |
| A1_1 | 1 | 12,693 | A2_6 | 1 | 21,755 | A3_12 | 1 | 6,955 |
| A1_1 | 139,683,647 | 139,686,935 | A3_6 | 1 | 13,725 | A4_12 | 3,028 | 12,051 |
| A2_1 | 133,539,140 | 133,542,428 | A4_6 | 1 | 16,156 | A1_13 | 7 | 3,579 |
| A2_1 | 1 | 4,377 | A1_7 | 1 | 10,577 | A1_13 | 115,565,603 | 115,571,113 |
| A3_1 | 1 | 17,077 | A2_7 | 1 | 10,577 | A2_13 | 1 | 8,450 |
| A4_1 | 859 | 8,395 | A3_7 | 120,015,310 | 120,030,727 | A2_13 | 102,441,727 | 102,447,237 |
| A1_2 | 1 | 16,657 | A3_7 | 1 | 41,084 | A3_13 | 1 | 8,450 |
| A1_2 | 146,290,819 | 146,291,878 | A1_8 | 1 | 17,427 | A4_13 | 1 | 18,465 |
| A2_2 | 1 | 17,900 | A1_8 | 122,767,494 | 122,796,747 | A4_13 | 101,899,690 | 101,906,133 |
| A2_2 | 134,030,115 | 134,031,174 | A2_8 | 110,121,004 | 110,135,662 | A1_14 | 1 | 7,550 |
| A3_2 | 1 | 15,903 | A2_8 | 1 | 38,704 | A1_14 | 111,668,055 | 111,681,930 |
| A3_2 | 139,017,237 | 139,026,315 | A4_8 | 1 | 18,762 | A2_14 | 97,024,336 | 97,038,189 |
| A4_2 | 4 | 21,326 | A2_9 | 119,493,660 | 119,503,231 | A2_14 | 1 | 11,987 |
| A1_3 | 1 | 17,439 | A3_9 | 118,523,137 | 118,532,708 | A3_14 | 1 | 7,550 |
| A2_3 | 144,772,015 | 144,780,291 | A4_9 | 1 | 15,753 | A3_14 | 105,290,489 | 105,303,090 |
| A3_3 | 1 | 15,578 | A4_9 | 109,253,711 | 109,260,087 | A4_14 | 112,954,183 | 112,968,104 |
| A4_3 | 128,282,961 | 128,292,758 | A1_10 | 1 | 9,549 | A1_15 | 1 | 17,841 |
| A4_3 | 11 | 15,008 | A2_10 | 1 | 6,539 | A1_15 | 110,150,466 | 110,164,700 |
| A1_4 | 135,070,333 | 135,086,988 | A3_10 | 1 | 4,877 | A2_15 | 1 | 17,840 |
| A1_4 | 4 | 11,196 | A4_10 | 1 | 4,877 | A2_15 | 60,987,292 | 61,001,526 |
| A2_4 | 1 | 10,713 | A1_11 | 2 | 710 | A3_15 | 1 | 25,109 |
| A2_4 | 118,201,443 | 118,225,800 | A1_11 | 121,154,745 | 121,155,482 | A3_15 | 108,221,432 | 108,234,062 |
| A3_4 | 126,604,162 | 126,612,967 | A2_11 | 100,807,578 | 100,817,779 | A4_15 | 1 | 19,617 |
| A4_4 | 113,478,304 | 113,496,553 | A3_11 | 114,352,953 | 114,357,926 | A4_15 | 99,776,464 | 99,789,094 |
| A4_4 | 1 | 12,599 | A3_11 | 2 | 710 | A1_16 | 1 | 41,665 |
| A1_5 | 129,519,955 | 129,532,503 | A4_11 | 1 | 19,920 | A1_16 | 85,090,113 | 85,101,359 |
| A2_5 | 1 | 37,169 | A4_11 | 111,491,310 | 111,508,266 | A2_16 | 83,174,013 | 83,177,593 |
| A2_5 | 127,039,792 | 127,041,058 | A1_12 | 1 | 6,955 | A2_16 | 1 | 41,665 |
| A3_5 | 1 | 5,922 | A2_12 | 1 | 13,161 | A3_16 | 1 | 14,125 |
| A1_6 | 1 | 16,157 | A3_12 | 105,758,339 | 105,769,758 | A4_16 | 1 | 12,227 |

Table S10. Positions of telomeres on the chromosomes of *C. bipinnatus*.

| **Chr ID** | **Start position** | **End position** |
| --- | --- | --- |
| Chr_1 | 1 | 12,933 |
| Chr_1 | 96,690,480 | 96,698,552 |
| Chr_2 | 1 | 14,562 |
| Chr_3 | 1 | 5,153 |
| Chr_4 | 1 | 17,425 |
| Chr_5 | 82,147,204 | 82,164,069 |
| Chr_6 | 1 | 22,817 |
| Chr_7 | 1 | 6,596 |
| Chr_8 | 77,639,119 | 77,652,263 |
| Chr_9 | 76,125,018 | 76,134,569 |
| Chr_10 | 1 | 23,092 |
| Chr_10 | 75,990,242 | 76,003,301 |
| Chr_11 | 73,169,530 | 73,178,904 |
| Chr_12 | 46,110,038 | 46,114,472 |

Table S11. Positions of telomeres on the chromosomes of *B. alba*.

| **Chr ID** | **Start position** | **End position** | **Chr ID** | **Start position** | **End position** |
| --- | --- | --- | --- | --- | --- |
| A1_1 | 90,490,097 | 90,500,070 | B1_6 | 1 | 23,364 |
| A2_1 | 1 | 12,462 | B1_6 | 84,693,968 | 84,704,860 |
| A2_1 | 87,621,353 | 87,631,165 | A1_7 | 75,928,698 | 75,944,478 |
| B1_1 | 85,201,661 | 85,212,742 | A2_7 | 71,315,345 | 71,322,868 |
| B2_1 | 1 | 19,365 | B1_7 | 75,633,217 | 75,648,328 |
| B2_1 | 83,559,641 | 83,570,715 | B2_7 | 75,138,584 | 75,152,532 |
| A1_2 | 86,762,003 | 86,779,898 | A1_8 | 75,116,541 | 75,126,414 |
| A2_2 | 1 | 24,079 | A2_8 | 1 | 14,076 |
| A2_2 | 85,165,321 | 85,186,813 | A2_8 | 67,404,646 | 67,405,598 |
| B1_2 | 70,061,595 | 70,080,681 | B1_8 | 1 | 14,076 |
| B2_2 | 1 | 24,079 | B2_8 | 1 | 22,735 |
| B2_2 | 64,575,361 | 64,594,447 | A1_9 | 74,621,517 | 74,644,457 |
| B1_3 | 691 | 21,185 | A2_9 | 1 | 30,063 |
| B1_3 | 82,922,156 | 82,934,635 | B1_9 | 1 | 30,063 |
| B2_3 | 1 | 8,094 | B1_9 | 71,266,711 | 71,287,851 |
| B2_3 | 77,346,387 | 77,364,298 | B2_9 | 66,450,283 | 66,473,223 |
| A1_4 | 1 | 14,713 | A1_10 | 1 | 22,070 |
| A2_4 | 76,774,530 | 76,780,559 | A2_10 | 1 | 20,038 |
| B1_4 | 1 | 23,171 | A1_11 | 1 | 18,161 |
| A1_5 | 1 | 11,519 | A2_11 | 72,117,727 | 72,142,266 |
| A1_5 | 80,400,394 | 80,409,357 | B1_11 | 1 | 18,161 |
| A2_5 | 1 | 19,542 | B1_11 | 69,340,099 | 69,355,279 |
| A2_5 | 79,765,300 | 79,775,741 | A1_12 | 68,996,087 | 69,012,368 |
| B1_5 | 1 | 7,873 | A2_12 | 1 | 27,584 |
| B1_5 | 75,896,901 | 75,919,905 | A2_12 | 58,845,521 | 58,860,587 |
| B2_5 | 73,126,507 | 73,148,867 | B1_12 | 67,119,866 | 67,132,519 |
| A1_6 | 1 | 12,113 | B2_12 | 65,641,675 | 65,651,113 |
| A2_6 | 1 | 12,113 |  |  |  |

Table S12. BUSCO assessment of the genomes of *D. pinnata*, *C. bipinnatus*, *B. alba* and other related species. Single-copy, Duplicated and Fragmented mean complete and single-copy BUSCOs, complete and duplicated BUSCOs, and fragmented BUSCOs, respectively. We used BUSCO v5.4.4 with lineage dataset embryophyta_odb10 to assess these genomes.

|  | **Genome** | **Complete** |  | **Single** |  | **Duplicated** |  | **Fragmented** |  | **Missing** |  | **Total** |
| --- | --- | --- | --- | --- | --- | --- | --- | --- | --- | --- | --- | --- |
| *D. pinnata* | A1 | 1523 | 94.4% | 1363 | 84.4% | 160 | 9.9% | 9 | 0.6% | 82 | 5.1% | 1614 |
|  | A2 | 1440 | 89.2% | 1308 | 81.0% | 132 | 8.2% | 11 | 0.7% | 163 | 10.1% | 1614 |
|  | A3 | 1501 | 93.0% | 1343 | 83.2% | 158 | 9.8% | 13 | 0.8% | 100 | 6.2% | 1614 |
|  | A4 | 1462 | 90.6% | 1319 | 81.7% | 143 | 8.9% | 10 | 0.6% | 142 | 8.8% | 1614 |
|  | A1A3 | 1595 | 98.8% | 191 | 11.8% | 1404 | 87.0% | 6 | 0.4% | 13 | 0.8% | 1614 |
|  | All | 1600 | 99.1% | 12 | 0.7% | 1588 | 98.4% | 5 | 0.3% | 9 | 0.6% | 1614 |
| *C. bipinnatus* | | 1572 | 97.4% | 1418 | 87.9% | 154 | 9.5% | 11 | 0.7% | 31 | 1.9% | 1614 |
| *B. alba* | A1 | 1532 | 94.9% | 1400 | 86.7% | 132 | 8.2% | 11 | 0.7% | 71 | 4.4% | 1614 |
|  | A2 | 1445 | 89.5% | 1340 | 83.0% | 105 | 6.5% | 7 | 0.4% | 162 | 10.0% | 1614 |
|  | B1 | 1440 | 89.2% | 1333 | 82.6% | 107 | 6.6% | 9 | 0.6% | 165 | 10.2% | 1614 |
|  | B2 | 1396 | 86.5% | 1291 | 80.0% | 105 | 6.5% | 11 | 0.7% | 207 | 12.8% | 1614 |
|  | A1B1 | 1588 | 98.4% | 244 | 15.1% | 1344 | 83.3% | 2 | 0.1% | 24 | 1.5% | 1614 |
|  | All | 1608 | 99.6% | 16 | 1.0% | 1592 | 98.6% | 1 | 0.1% | 5 | 0.3% | 1614 |
| *A. artemisiifolia* | | 1,379 | 85.4% | 1,275 | 79.0% | 104 | 6.4% | 101 | 6.3% | 134 | 8.3% | 1,614 |
| *H. annuus* | | 1,590 | 98.5% | 1,407 | 87.2% | 183 | 11.3% | 3 | 0.2% | 21 | 1.3% | 1,614 |
| *S. rebaudiana* | | 1,543 | 95.6% | 1,160 | 71.9% | 383 | 23.7% | 33 | 2.0% | 38 | 2.4% | 1,614 |
| *M. micrantha* | | 1,430 | 88.6% | 1,047 | 64.9% | 383 | 23.7% | 61 | 3.8% | 123 | 7.6% | 1,614 |
| *S. sonchifolius* | | 1,602 | 99.3% | 309 | 19.1% | 1,293 | 80.1% | 6 | 0.4% | 6 | 0.4% | 1,614 |
| *C. nankingense* | | 1,292 | 80.0% | 1,128 | 69.9% | 164 | 10.2% | 127 | 7.9% | 195 | 12.1% | 1,614 |
| *A. annua* | | 1,468 | 91.0% | 1,073 | 66.5% | 395 | 24.5% | 38 | 2.4% | 108 | 6.7% | 1,614 |
| *C. canadensis* | | 1,537 | 95.2% | 1,487 | 92.1% | 50 | 3.1% | 37 | 2.3% | 40 | 2.5% | 1,614 |
| *A. lappa* | | 1,566 | 97.0% | 1,482 | 91.8% | 84 | 5.2% | 21 | 1.3% | 27 | 1.7% | 1,614 |

Note: For tetraploid *D. pinnata* and *B. alba*, A1, A2, A3, A4, B1, B2 stands for different haplotypes.

Table S13. Public RNA-seq datasets statistics. We used these datasets for transcriptome assisted gene prediction.

| **Species** | **Data type** | **# of Bases** | **SRA accession** | **BioProject** |
| --- | --- | --- | --- | --- |
| *D. pinnata* | RNA-seq | 30.6 G | SRR18298150, SRR18298154, SRR18298140 | PRJNA811758 |
| *C. bipinnatus* | RNA-seq | 30.5 G | SRR12816006 | PRJNA636629 |
|  |  |  | SRR5237277 | PRJNA371565 |
|  |  |  | SRR3546768, SRR3546769 | PRJNA321371 |

Note: These datasets were downloaded from NCBI. Because there is no RNA-seq dataset for *B. alba*, we did not use this type of evidence for gene annotation of *B. alba*.

Table S14. Statistics of gene prediction evidence from full-length cDNA mapping.

|  | ***D. pinnata*** | ***C. bipinnatus*** | ***B. alba*** |
| --- | --- | --- | --- |
| CCS reads number | 258,035 | 156,571 | 983,117 |
| Iso-seq3 non-redundant isoforms | 39,277 | 25,818 | 145,889 |
| Ratio of non-redundant isoforms | 15.22% | 16.49% | 14.84% |
| Gmap+blat2hints.pl hits | 39,485 | 25,715 | 148124 |
| Ratio in total | 15.30% | 16.42% | 15.07% |
| Hints summary | | | |
| Total exon part hints | 72,774 | 44,954 | 253,159 |
| Total exon hints | 241,327 | 112,456 | 939,485 |
| Total intron hints | 276,233 | 133,289 | 1,058,071 |

Note: The reads of *B. alba* was much more than that of other plants. Because we did not have RNA-seq for *B. alba*, the more Iso-seq data would be very helpful to gene annotation of *B. alba*.

Table S15. Statistics of gene prediction evidence from homology alignment.

| **Species** | ***D. pinnata*** | ***C. bipinnatus*** | ***B. alba*** |
| --- | --- | --- | --- |
| *Helianthus annuus* | 184,583 | 101,293 | 179,342 |
| *Smallanthus sonchifolius* | 264,684 | 113,535 | 256,512 |
| *Stevia rebaudiana* | 145,951 | 64,418 | 142,961 |
| Hints summary | | | |
| Total CDSpart hints | 2,637,545 | 988,837 | 2,481,730 |
| Total intron hints | 2,042,327 | 709,591 | 1,902,915 |
| Total start hints | 413,151 | 177,115 | 385,870 |
| Total stop hints | 44,345 | 18,129 | 43,232 |

Table S16. Statistics of gene prediction evidence from RNA-seq mapping.

|  | ***D. pinnata*** | ***C. bipinnatus*** |
| --- | --- | --- |
| Number of reads | 101,995,107 | 126,987,381 |
| Average read length | 300 | 240 |
| Uniquely mapped reads number | 42,903,467 | 92,022,593 |
| Uniquely mapped reads % | 42.06% | 72.47% |
| Number of reads mapped to multiple loci | 51,800,846 | 5,684,230 |
| % of reads mapped to multiple loci | 50.79% | 4.48% |
| Hints summary | | |
| Total intron hints | 557,976 | 339,564 |

Note: We used datasets from Table S2 for generating RNA-seq hints.

Table S17. Gene statistics of *D. pinnata*, *C. bipinnatus*, *B. alba* and closely related plant species.

|  | **# of Genes** | **Average CDS Length** | **Average Number Exon of Gene** | **Total Length of Exon** | **Average Exon Length** | **Number of Intron** | **Average Intron Length** |
| --- | --- | --- | --- | --- | --- | --- | --- |
| *D. pinnata* | 181,915 | 1,234 | 4.94 | 224,643,068 | 250 | 716,615 | 438 |
| *C. bipinnatus* | 46,076 | 1,275 | 4.78 | 58,778,603 | 266 | 174,394 | 408 |
| *B. alba* | 165,431 | 1,095 | 4.48 | 181,196,996 | 244 | 575,335 | 445 |
| *H. annuus* | 57,126 | 1,248 | 4.33 | 71,300,168 | 288 | 190,220 | 782 |
| *M. micrantha* | 46,351 | 1,242 | 5.04 | 57,591,516 | 246 | 187,228 | 745 |
| *S. sonchifolius* | 89,960 | 1,091 | 5.06 | 98,187,351 | 215 | 365,506 | 528 |
| *S. rebaudiana* | 44,143 | 1,213 | 5.03 | 53,584,410 | 241 | 177,881 | 565 |
| *A. lappa* | 47,055 | 1,067 | 4.69 | 50,231,733 | 227 | 173,425 | 796 |
| *C. canadensis* | 45,483 | 1,915 | 5.49 | 87,137,144 | 349 | 204,046 | 703 |
| *C. nankingense* | 56,870 | 1,008 | 4.61 | 57,335,697 | 218 | 205,020 | 552 |
| *A. annua* | 63,226 | 1,125 | 5.07 | 71,182,002 | 221 | 257,511 | 726 |
| *A. artemisiifolia* | 37,038 | 1,170 | 5.49 | 43,336,128 | 212 | 166,427 | 609 |

Note: For tetraploid *D. pinnata* and *B. alba*, the gene sets also included allelic genes.

Table S18. BUSCO assessment of the gene sets of *D. pinnata*, *C. bipinnatus*, *B. alba* and other related species. Single-copy, Duplicated and Fragmented mean complete and single-copy BUSCOs, complete and duplicated BUSCOs, and fragmented BUSCOs, respectively. We used BUSCO v5.4.4 with lineage dataset embryophyta_odb10 to assess these gene sets.

|  | **Gene sets** | **Complete** |  | **Single** |  | **Duplicated** |  | **Fragmented** |  | **Missing** |  | **Total** |
| --- | --- | --- | --- | --- | --- | --- | --- | --- | --- | --- | --- | --- |
| *D. pinnata* | A1 | 1451 | 89.9% | 1299 | 80.5% | 152 | 9.4% | 36 | 2.2% | 127 | 7.9% | 1614 |
|  | A2 | 1387 | 85.9% | 1264 | 78.3% | 123 | 7.6% | 31 | 1.9% | 196 | 12.1% | 1614 |
|  | A3 | 1460 | 90.5% | 1324 | 82.0% | 136 | 8.4% | 31 | 1.9% | 123 | 7.6% | 1614 |
|  | A4 | 1405 | 87.1% | 1300 | 80.5% | 105 | 6.5% | 34 | 2.1% | 175 | 10.8% | 1614 |
|  | A1A3 | 1575 | 97.6% | 286 | 17.7% | 1289 | 79.9% | 14 | 0.9% | 25 | 1.5% | 1614 |
|  | All | 1597 | 98.9% | 32 | 2.0% | 1565 | 97.0% | 3 | 0.2% | 14 | 0.9% | 1614 |
| *C. bipinnatus* | | 1560 | 96.7% | 1399 | 86.7% | 161 | 10.0% | 24 | 1.5% | 30 | 1.9% | 1614 |
| *B. alba* | A1 | 1378 | 85.4% | 1264 | 78.3% | 114 | 7.1% | 84 | 5.2% | 152 | 9.4% | 1614 |
|  | A2 | 1277 | 79.1% | 1191 | 73.8% | 86 | 5.3% | 92 | 5.7% | 245 | 15.2% | 1614 |
|  | B1 | 1253 | 77.6% | 1150 | 71.3% | 103 | 6.4% | 104 | 6.4% | 257 | 15.9% | 1614 |
|  | B2 | 1231 | 76.3% | 1132 | 70.1% | 99 | 6.1% | 93 | 5.8% | 290 | 18.0% | 1614 |
|  | A1B1 | 1533 | 95.0% | 538 | 33.3% | 995 | 61.6% | 36 | 2.2% | 45 | 2.8% | 1614 |
|  | All | 1588 | 98.4% | 49 | 3.0% | 1539 | 95.4% | 13 | 0.8% | 13 | 0.8% | 1614 |
| *A. artemisiifolia* | | 1,379 | 85.4% | 1,275 | 79.0% | 104 | 6.4% | 101 | 6.3% | 134 | 8.3% | 1,614 |
| *H. annuus* | | 1,590 | 98.5% | 1,407 | 87.2% | 183 | 11.3% | 3 | 0.2% | 21 | 1.3% | 1,614 |
| *S. rebaudiana* | | 1,543 | 95.6% | 1,160 | 71.9% | 383 | 23.7% | 33 | 2.0% | 38 | 2.4% | 1,614 |
| *M. micrantha* | | 1,430 | 88.6% | 1,047 | 64.9% | 383 | 23.7% | 61 | 3.8% | 123 | 7.6% | 1,614 |
| *S. sonchifolius* | | 1,602 | 99.3% | 309 | 19.1% | 1,293 | 80.1% | 6 | 0.4% | 6 | 0.4% | 1,614 |
| *C. nankingense* | | 1,292 | 80.0% | 1,128 | 69.9% | 164 | 10.2% | 127 | 7.9% | 195 | 12.1% | 1,614 |
| *A. annua* | | 1,468 | 91.0% | 1,073 | 66.5% | 395 | 24.5% | 38 | 2.4% | 108 | 6.7% | 1,614 |
| *C. canadensis* | | 1,537 | 95.2% | 1,487 | 92.1% | 50 | 3.1% | 37 | 2.3% | 40 | 2.5% | 1,614 |
| *A. lappa* | | 1,566 | 97.0% | 1,482 | 91.8% | 84 | 5.2% | 21 | 1.3% | 27 | 1.7% | 1,614 |

Note: For tetraploid *D. pinnata* and *B. alba*, A1, A2, A3, A4, B1, B2 stands for different haplotypes.

Table S19. Statistics of functional annotation for the gene sets based on different databases.

|  | ***D. pinnata*** | ***C. bipinnatus*** | ***B. alba*** |
| --- | --- | --- | --- |
| Total gene number | 181,915 | 46,076 | 165,443 |
| NR hit | 159,897 | 39,062 | 136,285 |
| NR hit % | 87.90% | 84.78% | 82.38% |
| KEGG hit | 125,672 | 30,616 | 108,393 |
| KEGG hit % | 69.08% | 66.45% | 65.52% |
| Uniprot (Swiss-Prot) hit | 122,957 | 31,229 | 112,430 |
| Uniprot (Swiss-Prot) hit % | 67.59% | 67.78% | 67.96% |
| InterPro hits | 171,204 | 43,610 | 149,700 |
| InterPro hits % | 94.11% | 94.65% | 90.48% |
| InterPro term | 134,211 | 34,586 | 123,587 |
| InterPro term % | 73.77% | 75.06% | 74.70% |
| GO terms | 99,102 | 25,626 | 92,059 |
| GO terms % | 54.48% | 55.62% | 55.64% |
| All function | 173,686 | 44,074 | 151,025 |
| All function % | 95.48% | 95.66% | 91.29% |

Note: For tetraploid *D. pinnata* and *B. alba*, the gene sets also included allelic genes.

Table S20. Statistics of annotated transcription factors.

| **TF family** | ***D. pinnata*** | ***C. bipinnatus*** | ***B. alba*** | **TF family** | ***D. pinnata*** | ***C. bipinnatus*** | ***B. alba*** |
| --- | --- | --- | --- | --- | --- | --- | --- |
| bHLH | 802 | 218 | 754 | ZF-HD | 71 | 22 | 83 |
| ERF | 789 | 229 | 914 | CO-like | 70 | 17 | 70 |
| MYB | 709 | 186 | 684 | NF-YA | 63 | 12 | 66 |
| C2H2 | 662 | 155 | 556 | GeBP | 62 | 10 | 46 |
| NAC | 472 | 132 | 506 | HB-other | 61 | 9 | 37 |
| WRKY | 427 | 115 | 395 | DBB | 49 | 12 | 38 |
| bZIP | 403 | 110 | 339 | YABBY | 48 | 13 | 24 |
| MYB_related | 330 | 86 | 366 | BES1 | 45 | 12 | 51 |
| GRAS | 302 | 79 | 299 | E2F/DP | 45 | 12 | 41 |
| G2-like | 244 | 70 | 237 | ARR-B | 44 | 18 | 42 |
| HD-ZIP | 237 | 69 | 251 | NF-YC | 44 | 12 | 42 |
| C3H | 234 | 64 | 202 | GRF | 40 | 16 | 41 |
| LBD | 215 | 62 | 284 | SRS | 38 | 9 | 39 |
| Dof | 168 | 48 | 174 | BBR-BPC | 24 | 8 | 30 |
| B3 | 167 | 54 | 189 | CAMTA | 24 | 8 | 25 |
| TCP | 164 | 40 | 199 | CPP | 24 | 10 | 20 |
| Trihelix | 162 | 49 | 181 | LSD | 23 | 8 | 16 |
| GATA | 156 | 43 | 144 | EIL | 18 | 5 | 22 |
| MIKC_MADS | 155 | 48 | 130 | RAV | 15 | 5 | 19 |
| Nin-like | 137 | 24 | 100 | VOZ | 12 | 3 | 11 |
| ARF | 136 | 41 | 155 | HB-PHD | 8 | 2 | 7 |
| FAR1 | 130 | 120 | 96 | S1Fa-like | 8 | 2 | 7 |
| TALE | 130 | 36 | 131 | SAP | 8 | 1 | 10 |
| AP2 | 128 | 35 | 130 | NF-X1 | 7 | 2 | 8 |
| HSF | 121 | 35 | 113 | Whirly | 7 | 2 | 6 |
| NF-YB | 113 | 28 | 103 | HRT-like | 4 | 1 | 4 |
| M-type_MADS | 95 | 37 | 149 | LFY | 4 | 1 | 2 |
| SBP | 93 | 22 | 82 | STAT | 4 | 1 | 3 |
| WOX | 74 | 17 | 62 |  |  |  |  |

Note: The TFs were annotated by PlantTFDB (<http://planttfdb.gao-lab.org/prediction.php>).

Table S21. The summary of annotated tRNAs.

| **tRNA name** | ***D. pinnata*** | ***C. bipinnatus*** | ***B. alba*** |
| --- | --- | --- | --- |
| tRNA-Ala | 241 | 69 | 231 |
| tRNA-Arg | 292 | 72 | 294 |
| tRNA-Asn | 174 | 43 | 181 |
| tRNA-Asp | 353 | 68 | 238 |
| tRNA-Cys | 148 | 37 | 92 |
| tRNA-Gln | 143 | 55 | 148 |
| tRNA-Glu | 278 | 78 | 260 |
| tRNA-Gly | 344 | 131 | 316 |
| tRNA-His | 123 | 48 | 102 |
| tRNA-Ile | 189 | 70 | 314 |
| tRNA-iMet | 115 | 22 | 81 |
| tRNA-Leu | 329 | 92 | 346 |
| tRNA-Lys | 235 | 66 | 223 |
| tRNA-Met | 191 | 58 | 285 |
| tRNA-Phe | 164 | 50 | 151 |
| tRNA-Pro | 338 | 71 | 259 |
| tRNA-Ser | 296 | 74 | 305 |
| tRNA-Thr | 271 | 54 | 198 |
| tRNA-Trp | 120 | 28 | 96 |
| tRNA-Tyr | 108 | 39 | 128 |
| tRNA-Val | 249 | 249 | 747 |
| tRNA-Sup | 14 | 0 | 3 |
| tRNA-SeC | 2 | 0 | 0 |
| Total | 4,717 | 1,474 | 4,998 |

Note: tRNA-Sup is suppressor tRNA. tRNA-iMet is initial tRNA-Met. tRNA-SeC is Selenocysteine tRNAs.

Table S22. The summary of annotated rRNAs and other ncRNAs.

|  | ***D. pinnata*** | ***C. bipinnatus*** | ***B. alba*** |  |
| --- | --- | --- | --- | --- |
| rRNAs | | | | |
| 5S rRNA | 29,533 | 4,337 | 5,495 |  |
| 5.8S rRNA | 6,872 | 2,168 | 5,344 |  |
| 18S rRNA | 7,453 | 2,169 | 5,366 |  |
| 28S rRNA | 7,456 | 2,154 | 5,283 |  |
| Total rRNA | 51,314 | 10,828 | 21,488 |  |
| ncRNA | | | | |
| snoR71 | 4,608 | 219 | 910 |  |
| Intron_gpII | 934 | 219 | 1,911 |  |
| U2 | 899 | 157 | 191 |  |
| Hammerhead_3 | 430 | 90 | 91 |  |
| U5 | 368 | 77 | 196 |  |
| U1 | 313 | 54 | 149 |  |
| U6 | 222 | 77 | 243 |  |
| U4 | 209 | 41 | 69 |  |
| Plant_SRP | 164 | 16 | 78 |  |
| MIR169_5 | 150 | 14 | 72 |  |
| snoZ103 | 125 | 22 | 44 |  |
| mir-395 | 115 | 2 | 69 |  |
| Plant_U3 | 101 | 34 | 77 |  |
| IsrR | 100 | 14 | 96 |  |
| Histone3 | 87 | 17 | 28 |  |
| MIR169_2 | 83 | 22 | 73 |  |
| snoZ43 | 79 | 5 | 16 |  |
| snoR60 | 78 | 4 | 18 |  |
| snosnR60_Z15 | 77 | 4 | 23 |  |
| SNOR75 | 62 | 7 | 19 |  |
| MIR159 | 61 | 21 | 61 |  |
| Intron_gpI | 60 | 9 | 102 |  |
| mir-399 | 57 | 13 | 50 |  |
| SNORD14 | 56 | 17 | 31 |  |
| MIR171_1 | 44 | 11 | 67 |  |
| others | 1,740 | 401 | 1,144 |  |
| Total ncRNA | 11,222 | 1,567 | 5,828 |  |

Table S23. Statistics of annotated transposable elements.

|  | ***D. pinnata*** | | ***C. bipinnatus*** | | ***B. alba*** | |
| --- | --- | --- | --- | --- | --- | --- |
| TE class | Length | Percent | Length | Percent | Length | Percent |
| LTR | 5,531,300,758 | 70.36% | 640,255,436 | 63.05% | 1,921,843,420 | 51.14% |
| DNA | 1,256,785,313 | 15.99% | 158,408,638 | 15.60% | 936,053,978 | 24.91% |
| MITE | 65,474,149 | 0.83% | 12,048,672 | 1.19% | 51,582,284 | 1.37% |
| LINE | 30,161,519 | 0.38% | 4,533,738 | 0.45% | 24,682,382 | 0.66% |
| RC | 4,103,199 | 0.05% | 450,720 | 0.04% | 4,560,814 | 0.12% |
| SINE | 2,864,529 | 0.04% | 726,486 | 0.07% | 3,155,992 | 0.08% |

Table S24. Statistics of closely related genomes.

| **Species Name** | **Trib. / Genus** | **Ploidy** | **Sequencing technology** | **Genome Size (G)** | **Assembly size (G)** | **GC percent** | **Contig N50 size (M)** | **Reference** |
| --- | --- | --- | --- | --- | --- | --- | --- | --- |
| *D. pinnata* | Coreopsideae / Dahlia | 2n=4x=64 | PacBio HiFi + HiC | 3.98 | 7.88 | 37.81% | 29,279,538 | This study |
| *C. bipinnatus* | Coreopsideae / Cosmos | 2n=2x=24 | PacBio HiFi + HiC | 1.08 | 1.02 | 36.83% | 52,817,601 | This study |
| *B. alba* | Coreopsideae / Bidens | 2n=4x=48 | PacBio HiFi + HiC | 1.93 | 3.75 | 36.64% | 15,049,174 | This study |
| *A. artemisiifolia* | Heliantheae / Ambrosia | 2n=2x=36 | PacBio HiFi + Omni-C | 1.15 | 1.11 | 37.69% | 15,452,185 | (Battlay et al., 2023) |
| *H. annuus* | Heliantheae / Helianthus | 2n=2x=34 | PacBio CLR + genetic maps | 3.6 | 3.01 | 38.59% | 2,004,740 | (Badouin et al., 2017) |
| *S. rebaudiana* | Eupatorieae / Stevia | 2n=2x=22 | PacBio CLR + Hi-C | 1.16 | 1.42 | 36.98% | 664,928 | (Xu et al., 2021) |
| *M. micrantha* | Eupatorieae / Mikania | 2n=2x=38 | PacBio CLR + Hi-C | 1.86 | 1.79 | 36.20% | 1,353,263 | (Liu et al., 2020) |
| *S. sonchifolius* | Millerieae / Smallanthus | 2n=4x=58 | PacBio HiFi + HiC | 2.6 | 2.72 | 37.44% | 87,391,069 | (Fan et al., 2022) |
| *C. nankingense* | Anthemideae / Chrysanthemum | 2n=2x=18 | Nanopore + Illumina | 3.07 | 2.53 | 35.62% | 130,667 | (Song et al., 2018) |
| *A. annua* | Anthemideae / Artemisia | 2n=2x=18 | Illumina + Roche 454 + PacBio | 1.76 | 1.79 | 35.34% | 9,093 | (Shen et al., 2018) |
| *C. canadensis* | Astereae / Conyza | 2n=2x=18 | PacBio CLR + Hi-C | 0.47 | 0.43 | 34.08% | 1,620,735 | (Laforest et al., 2020) |
| *A. lappa* | Cardueae / Arctium | 2n=2x=36 | PacBio HiFi + HiC | 1.8 | 1.73 | 36.65% | 74,692,580 | (Fan et al., 2022) |

Note: For tetraploid *D. pinnata* and *B. alba*, the allelic chromosomes were built. Thus, the estimated genome size is about half of the assembly size.

Table S25. Statistics of orthogroups in different plants defined by OrthoFinder.

|  | **Number of genes** | **Number of genes in orthogroups** | **Number of unassigned genes** | **Percentage of genes in orthogroups** | **Percentage of unassigned genes** | **Number of orthogroups containing species** | **Percentage of orthogroups containing species** | **Number of species-specific orthogroups** | **Number of genes in species-specific orthogroups** | **Percentage of genes in species-specific orthogroups** |
| --- | --- | --- | --- | --- | --- | --- | --- | --- | --- | --- |
| *A. artemisiifolia* | 36,641 | 32,589 | 4,052 | 88.9 | 11.1 | 16,209 | 39.5 | 640 | 2,038 | 5.6 |
| *A. lappa* | 47,055 | 41,023 | 6,032 | 87.2 | 12.8 | 18,707 | 45.6 | 1,203 | 6,974 | 14.8 |
| *A. annua* | 63,226 | 59,304 | 3,922 | 93.8 | 6.2 | 21,382 | 52.2 | 1,662 | 7,423 | 11.7 |
| *C. nankingense* | 56,870 | 53,601 | 3,269 | 94.3 | 5.7 | 19,926 | 48.6 | 1,075 | 4,949 | 8.7 |
| *C. canadensis* | 45,483 | 44,287 | 1,196 | 97.4 | 2.6 | 15,982 | 39 | 488 | 9,451 | 20.8 |
| *D. pinnata* | 86,256 | 78,653 | 7,603 | 91.2 | 8.8 | 20,685 | 50.5 | 1,989 | 6,939 | 8 |
| *H. annuus* | 57,126 | 55,803 | 1,323 | 97.7 | 2.3 | 17,995 | 43.9 | 477 | 4,718 | 8.3 |
| *M. micrantha* | 46,351 | 44,458 | 1,893 | 95.9 | 4.1 | 17,090 | 41.7 | 909 | 4,571 | 9.9 |
| *S. sonchifolius* | 89,960 | 81,949 | 8,011 | 91.1 | 8.9 | 22,088 | 53.9 | 2,203 | 9,450 | 10.5 |
| *S. rebaudiana* | 44,143 | 42,343 | 1,800 | 95.9 | 4.1 | 17,523 | 42.7 | 444 | 1,253 | 2.8 |
| *V. vinifera* | 25,676 | 24,401 | 1,275 | 95 | 5 | 14,570 | 35.5 | 533 | 3,197 | 12.5 |
| *B. alba* | 81,835 | 78,315 | 3,520 | 95.7 | 4.3 | 21,505 | 52.5 | 2,196 | 11,117 | 13.6 |
| *C. bipinnata* | 46,076 | 44,593 | 1,483 | 96.8 | 3.2 | 18,288 | 44.6 | 349 | 3,416 | 7.4 |

Note: For auto-tetraploid *D. pinnata*, genes from haplotype A1 and A3 were used for this analysis. For allo-tetraploid *B. alba*, genes from haplotype A1 and B1 were used for this analysis. Fifty percent of all genes were in orthogroups with 29 or more genes (G50 was 29) and were contained in the largest 5,895 orthogroups (O50 was 5,895).

Table S26. Gene ID of inulin metabolism genes and transcription factors.

|  | ***D. pinnata*** | ***C. bipinnatus*** | ***B. alba*** |
| --- | --- | --- | --- |
| 1-SST | g7971.t1, g72038.t1 | g6467.t1 | g87334.t1, g31431.t1 |
| 1-FFT | g7978.t1, g72037.t1 | g6466.t1 | g141421.t1, g142078.t1 |
| 1-FEH1 | g170832.t1 | g16289.t1 | g135473.t1, g45620.t1 |
| 1-FEH2 | g102377.t1 | g620.t1 | g127989.t1, g115916.t1 |
| MYB3 | g139364.t1, g40545.t1 | g45853.t1 | g161352.t1, g140656.t1 |
| MYB5 | g95690.t1, g151251.t1 | g28332.t1 | g70126.t1, g647.t1 |
| MYB17 | g138339.t1, g15121.t1 | g41241.t1 | g95583.t1, g4765.t1 |

Table S27. The Pfam domain annotation of 1-FEH1 and 1-FEH2.

| 1-FEH1 gene | protein length | Pfam ID | Domain description | Start | End | E-value |
| --- | --- | --- | --- | --- | --- | --- |
| Dpin.g157936 | 301 | PF00251 | Glycosyl hydrolases family 32 N-terminal domain | 50 | 93 | 9.90E-08 |
| Dpin.g157936 | 301 | PF08244 | Glycosyl hydrolases family 32 C terminal | 96 | 291 | 3.30E-26 |
| Dpin.g170832 | 559 | PF00251 | Glycosyl hydrolases family 32 N-terminal domain | 34 | 351 | 4.00E-97 |
| Dpin.g170832 | 559 | PF08244 | Glycosyl hydrolases family 32 C terminal | 354 | 549 | 8.50E-28 |
| Cbip.g16289 | 523 | PF00251 | Glycosyl hydrolases family 32 N-terminal domain | 36 | 283 | 2.90E-69 |
| Cbip.g16289 | 523 | PF08244 | Glycosyl hydrolases family 32 C terminal | 319 | 513 | 3.00E-27 |
| Balb.g135473 | 558 | PF00251 | Glycosyl hydrolases family 32 N-terminal domain | 35 | 351 | 1.50E-97 |
| Balb.g135473 | 558 | PF08244 | Glycosyl hydrolases family 32 C terminal | 354 | 548 | 3.10E-26 |
| Balb.g45620 | 509 | PF00251 | Glycosyl hydrolases family 32 N-terminal domain | 1 | 302 | 1.20E-86 |
| Balb.g45620 | 509 | PF08244 | Glycosyl hydrolases family 32 C terminal | 305 | 499 | 1.90E-26 |
| 1-FEH2 gene | protein length | Pfam ID | Domain description | Start | End | E-value |
| Dpin.g102377 | 580 | PF00251 | Glycosyl hydrolases family 32 N-terminal domain | 50 | 367 | 6.00E-97 |
| Dpin.g102377 | 580 | PF08244 | Glycosyl hydrolases family 32 C terminal | 370 | 564 | 6.50E-26 |
| Dpin.g32657 | 238 | PF08244 | Glycosyl hydrolases family 32 C terminal | 28 | 222 | 7.90E-28 |
| Cbip.g620 | 525 | PF00251 | Glycosyl hydrolases family 32 N-terminal domain | 4 | 312 | 2.10E-90 |
| Cbip.g620 | 525 | PF08244 | Glycosyl hydrolases family 32 C terminal | 315 | 509 | 9.30E-27 |
| Balb.g115916 | 524 | PF00251 | Glycosyl hydrolases family 32 N-terminal domain | 1 | 311 | 2.90E-92 |
| Balb.g115916 | 524 | PF08244 | Glycosyl hydrolases family 32 C terminal | 314 | 508 | 7.00E-27 |
| Balb.g127989 | 524 | PF00251 | Glycosyl hydrolases family 32 N-terminal domain | 1 | 311 | 3.60E-92 |
| Balb.g127989 | 524 | PF08244 | Glycosyl hydrolases family 32 C terminal | 314 | 508 | 9.40E-27 |

**Reference:**

Badouin, H., Gouzy, J., Grassa, C. J., Murat, F., Staton, S. E., Cottret, L., . . . Langlade, N. B. (2017). The sunflower genome provides insights into oil metabolism, flowering and Asterid evolution. *Nature, 546*(7656), 148-+. doi:10.1038/nature22380

Battlay, P., Wilson, J., Bieker, V. C., Lee, C., Prapas, D., Petersen, B., . . . Hodgins, K. A. (2023). Large haploblocks underlie rapid adaptation in the invasive weed Ambrosia artemisiifolia. *Nat Commun, 14*(1), 1717. doi:10.1038/s41467-023-37303-4

Fan, W., Wang, S., Wang, H., Wang, A., Jiang, F., Liu, H., . . . Zhang, Y. (2022). The genomes of chicory, endive, great burdock and yacon provide insights into Asteraceae palaeo-polyploidization history and plant inulin production. *Molecular Ecology Resources, 22*(8), 3124-3140. doi:<https://doi.org/10.1111/1755-0998.13675>

Laforest, M., Martin, S. L., Bisaillon, K., Soufiane, B., Meloche, S., & Page, E. (2020). A chromosome-scale draft sequence of the Canada fleabane genome. *Pest Management Science, 76*(6), 2158-2169. doi:<https://doi.org/10.1002/ps.5753>

Liu, B., Yan, J., Li, W., Yin, L., Li, P., Yu, H., . . . Wan, F. (2020). Mikania micrantha genome provides insights into the molecular mechanism of rapid growth. *Nature Communications, 11*(1), 340. doi:10.1038/s41467-019-13926-4

Shen, Q., Zhang, L., Liao, Z., Wang, S., Yan, T., Shi, P., . . . Tang, K. (2018). The Genome of Artemisia annua Provides Insight into the Evolution of Asteraceae Family and Artemisinin Biosynthesis. *Molecular Plant, 11*(6), 776-788. doi:<https://doi.org/10.1016/j.molp.2018.03.015>

Song, C., Liu, Y., Song, A., Dong, G., Zhao, H., Sun, W., . . . Chen, S. (2018). The Chrysanthemum nankingense Genome Provides Insights into the Evolution and Diversification of Chrysanthemum Flowers and Medicinal Traits. *Molecular Plant, 11*(12), 1482-1491. doi:<https://doi.org/10.1016/j.molp.2018.10.003>

Xu, X. Y., Yuan, H. Y., Yu, X. Q., Huang, S. Z., Sun, Y. M., Zhang, T., . . . Yang, Y. H. (2021). The chromosome-level Stevia genome provides insights into steviol glycoside biosynthesis. *Horticulture Research, 8*(1). doi:10.1038/s41438-021-00565-4
