# Supplementary material for: The genomes of Dahlia pinnata, Cosmos bipinnatus, and Bidens alba in tribe Coreopsideae provide insights into polyploid evolution and inulin biosynthesis
Source: Gigascience. 2024 Jun 13;13:giae032. doi: 10.1093/gigascience/giae032 (PMC11170221; doi:10.1093/gigascience/giae032)
Supplement: giae032_GIGA-D-23-00392_Revision_1 [file giae032_giga-d-23-00392_revision_1.pdf]

GigaScience

The genomes of Dahlia pinnata, Cosmos bipinnatus and Bidens alba in tribe Coreopsideae provide insights into polyploid evolution and inulin biosynthesis

--Manuscript Draft--

Manuscript Number:	GIGA-D-23-00392R1
Full Title:	The genomes of Dahlia pinnata, Cosmos bipinnatus and Bidens alba in tribe Coreopsideae provide insights into polyploid evolution and inulin biosynthesis
Article Type:	Research

Funding Information:	Funder	Grant
	the National Key R&D Program of China (2021YFC2600101)	Not applicable
	the Agricultural Science and Technology Innovation Program of CAAS	Not applicable
	the Elite Young Scientists Program of CAAS	Not applicable
	Key Laboratory of Shenzhen (ZDSYS20141118170111640)	Not applicable

Abstract:

Background: The Coreopsideae tribe, a subset of the Asteraceae family, encompasses economically vital genera like Dahlia, Cosmos, and Bidens, which are widely employed in medicine, horticulture, ecology, and food applications. Nevertheless, the lack of reference genomes hinders evolutionary and biological investigations in this tribe.
Results: Here, we present three haplotype-resolved chromosome-level reference genomes of the tribe Coreopsideae, including two famous flower plants Dahlia pinnata and Cosmos bipinnatus, and one invasive weed plant Bidens alba, with assembled genome sizes 3.93 G, 1.02 G and 1.87 G, respectively. We found that Gypsy transposable elements contribute mostly to the larger genome size of D. pinnata, and multiple chromosome rearrangements have occurred in tribe Coreopsideae. Besides the shared whole genome duplication (WGD-2) in the Heliantheae alliance, our analyses showed that D. pinnata and B. alba each underwent an independent recent WGD-3 event: in D. pinnata it is more likely to be a self-WGD, while in B. alba it is from the hybridization of two ancestor species. Further, we identified key genes in the inulin metabolic pathway, and found that the pseudogenization of 1-FEH1 and 1-FEH2 genes in D. pinnata and the deletion of three key residues of 1-FFT proteins in C. bipinnatus and B. alba may probably explain why D. pinnata produces much more inulin than the other two plants.
Conclusions: Collectively, the genomic resources for the Coreopsideae tribe will promote phylogenomics in Asteraceae plants, facilitate ornamental molecular breeding improvements and inulin production, and help prevent invasive weeds.

Corresponding Author:	Wei Fan<br>Chinese Academy of Agricultural Sciences<br>shenzhen, guangdong CHINA
Corresponding Author Secondary Information:	
Corresponding Author's Institution:	Chinese Academy of Agricultural Sciences
Corresponding Author's Secondary Institution:	
First Author:	Hengchao Wang
First Author Secondary Information:	
Order of Authors:	Hengchao Wang
	Dong Xu
	Fan Jiang
	Sen Wang
	Anqi Wang

Powered by Editorial Manager® and ProduXion Manager® from Aries Systems Corporation

	Hangwei Liu
	Lihong Lei
	Wanqiang Qian
	Wei Fan
**Order of Authors Secondary Information:**	
**Response to Reviewers:**	Reviewer reports:<br><br>Reviewer #1: Summary<br>Wang et. al focuses on the assembly, annotation and analysis of three species within the Coreopsideae tribe of the Asteraceae family; Dahlia pinnata, Cosmos bipinnatus, and Bidens alba. With only one genome assembly reported in this tribe previously (Bidens hawaiensis), these genomes represent a big step in understanding the biology of species in this tribe. Using chromosome-level assemblies, the authors find that: D. pinnata is an autotetraploid, B. alba is an allotetraploid, the genera Cosmos and Bidens are more closely related (diverged ~7mya) than either is to Dahlia, and take an initial dive into the biosynthesis of inulin and how it may be differentially regulated across these species. While the data is of high quality and the analysis is thorough, there are a few places where clarification is required as detailed below:<br><br>Major Comments<br>* Double check the Bidens picture in Figure1 as it appears to be Bidens pilosa and not Bidens alba (B. alba has only two awns per achene compared to 3 or more in B. pilosa). Contrary to the text, B. alba should not be considered a variety of B. pilosa (which is hexaploid) and should be referred only as B. alba.<br>Reply: We have double checked our sample and the picture. The Bidens studied here is B. alba, as confirmed by the karyotype analysis. In the old picture, most achenes have two awns, but some seem have three awns, which may be caused by low resolution. We used a new Bidens picture with high resolution, and removed the description taking B. alba as a variety of B. pilosa.<br><br><br>* It's confusing if data presented are representing haplotypes of a single subgenome or if haplotypes were "forced" within subgenomes to compare across each (for Dahlia and Bidens). While the subgenome discussion comes later, this distinction needs to be made and be clear for data in Figures 1 and S3<br>Reply: Thank you for this suggestion. We have added the explanation “Note that the reference genomes for each species were shown. C. bipinnata is diploid, so the haploid genome was used as reference genome. In contrast, D. pinnata and B. alba are tetraploids, their haploid genomes containing two subgenomes were used as their reference genomes.” in the figure legends of Figures 1, and have added the explanation “As C. bipinnata is diploid, so the haploid genome was used as reference genome. In contrast, D. pinnata and B. alba are tetraploids, the heatmap showed Hi-C signals among all chromosomes.” in the figure legends of Figure S3.<br><br><br>* When using Orthofinder to identify orthologs across Asteraceae genomes (for analyses in Figs 3 and S6), were genes pulled from annotations derived from across subgenomes or were a single subgenome represented in these analyses? If gene annotations were used as defined in Lines 139-141, this could greatly reduce power for identifying single-gene orthogroups for phylogenomic comparisons and has the potential to skew the analyses. Could species level comparisons be done for single haplotypes instead?<br>Reply: For Othofinder analyses, all species used gene annotations from their reference (haploid) genomes. As D. pinnata and B. alba are tetraploid, the gene annotations from both their two subgenomes were included. The orthologous group construction is not afraid of polyploids, because that all the duplicated genes will be assigned into the same orthologous group. To identify the single-copy orthologous group, the diploid species required to have one gene copy, while the tetraploid species (D. pinnata and B. alba) required to have one or two gene copy, and then only one of the two gene copies was kept by random selection. Therefore, our method is an alternative method to that of using a single haplotype for tetraploid species.

Because tetraploids usually lost some duplicated genes through evolution, genes on single haplotype may be incomplete, as shown in Figure 1E. If we use the genes on single haplotype, we may have the risk of losing some orthogroups.

* As subgenomes of either Dahlia or Bidens could not be fully phased using Kmer-based methods, are there other methods that could be explored for this phasing? With data totals from both Hifi and HiC presented, I would expect subgenomes to be resolvable. Especially in the case of an allotetraploid like B. alba. More clarification as to how these methods work (or don't work) would aid in understanding the authors analyses. Additionally, some attempt with at least one other method is encouraged since this is a main take-away from the work.
Reply: HiFi and HiC can only resolve the haplotype assembly, but will not help in dividing subgenomes.

To divide subgenomes of polyploids, current methods mostly utilized the structure difference between the subgenomes. Kmer-based methods depended on the high frequent transposable elements (TEs), which account for most of genomes. However, these methods have a prerequisite: the sequence difference between the two subgenomes should be large enough, i.e. the two ancestors should have experienced different evolution history and accumulated enough differences. Because all the three plants in this study expanded TEs very recently, the difference are not large enough, as showed in Figure 2 and Figure S5, we cannot divide the subgenomes for Dahlia or Bidens by the Kmer-based methods, or any other methods by comparing the structure difference between the subgenomes.

A more effective way to distinguish subgenomes is by sequencing and analyzing their ancestor genomes. However, this is out of ability at current stage. In future, with the availability of more diploid genomes in this tribe Coreopsideae, we will eventually be able to divide the subgenomes by comparing with their ancestor species.

* The inulin biosynthesis pathway is an interesting one to highlight among these genomes! For the pseudogene determination of 1-FEH1 and 1-FEH2 in Dahlia, did you look at more than predicted gene translation output from Orthofinder? What does the gene sequence say in comparison to the truncated predicted translations used for align in Figs. S13-S14? These should be reported, or at least mentioned, in determining that these copies are pseudogenized (and to what extent)
Reply: We aligned the collected public genes to the reference genomes to manually identify any missing genes of the gene sets and to check the gene structures.

We added a new Table S27, showing the Pfam domain annotation of 1-FEH1 and 1-FEH2. Genes of glycosyl hydrolases family 32 contains conservative N-terminal domain and C terminal. However, the N-terminal domain of Dpin.g157936 (1-FEH1) was severely truncated, and the N-terminal domain of Dpin.g32657 was even lost. Thus, these two genes must be pseudogenized.

* In a similar vein, It would be interesting to know if there are any genomic scars in the Bidens FFT and SST loci corresponding to where these genes may have been right next to each other on each chromosome. Since Cosmos and Bidens are more closely related, and even Dahlia shares this organization, I would think there would be some evidence of this at the genomic level looking at gene synteny across these regions.
Reply: Yes, you are right. There are pseudogenized 1-FFT genes nearing 1-SST genes on ChrA1-10 and ChrB1-10 of Bidens. The synteny across these regions were shown in Figure 6B. We now highlighted the pseudogenes in red, and added a Supplemental Figure S14 to indicate that these pseudogenes lost the first of the catalytic triads, which is necessary for their function. Considering the Bidens is an invasive weed and has the fastest mutation rate, it is not strange that the functional genes 1-SST and 1-FFT located on different chromosomes.

Minor Comments

Powered by Editorial Manager® and ProduXion Manager® from Aries Systems Corporation

* Lines 66-67 - C3 and C4 mention feels out of place
Reply: We removed this sentence.

* Lines 227-229 - unclear what is meant by "1 haplotype and 2 haplotypes", please clarify
Reply: One haplotype means one subgenome, and two haplotypes mean two subgnomes. In tetraploid species, there are four haplotypes in the nucleus. We clarified the haplotype in the text, and revised the sentence to “we analyzed the distribution of synonymous substitution rate (Ks) of homologous gene pairs within one haplotype (subgenome) of D. pinnata, two haplotypes of D. pinnata, C. bipinnata, and H. annuus. In comparison, we also analyzed the distribution of Ks between one haplotype of D. pinnata and C. bipinnata.”.

* More introduction to inulin (type of compound, biosynthesis, etc) early on may help bridge the readers understanding of why it's a primary focus of the manuscript
Reply: We added the following sentence to describe the function of inulin in the second paragraph of the Introduction section. “In addition, most plants in the Asteraceae employed inulin as the primary reserve carbohydrates, instead of starches, which may contribute to strong capability of environmental adaptation for these plants [14-17].”
References cited are flowing:

14.Fan W, Wang S, Wang H, Wang A, Jiang F, Liu H, et al. The genomes of chicory, endive, great burdock and yacon provide insights into Asteraceae palaeo-polyploidization history and plant inulin production. Molecular Ecology Resources. 2022;22 8:3124-40. doi:https://doi.org/10.1111/1755-0998.13675.
15.Shen F, He H, Huang X, Deng Y and Yang XZ. Insights into the convergent evolution of fructan biosynthesis in angiosperms from the highly characteristic chicory genome. New Phytologist. 2023;238 3:1245-62. doi:10.1111/nph.18796.
16.Valluru R and Van den Ende W. Plant fructans in stress environments: emerging concepts and future prospects. Journal of Experimental Botany. 2008;59 11:2905-16. doi:10.1093/jxb/ern164.
17.Wang S, Wang A, Chen R, Xu D, Wang H, Jiang F, et al. Haplotype-resolved chromosome-level genome of hexaploid Jerusalem artichoke provides insights into its origin, evolution, and inulin metabolism. Plant Communications. 2023:100767. doi:https://doi.org/10.1016/j.xplc.2023.100767.

* Lines 307-308 - Rewrite for clarity. TF regulating these same genes mentioned before?
Reply: We rewrite the sentence “In addition to these functional genes, previous studies also reported some important transcription factors regulating the expression of the key functional genes.” as “In addition to these functional genes, previous studies also reported in chicory, MYB3 and MYB5 can enhance promoter activities of 1-FEH genes in response to abiotic stress and hormonal cues, while MYB17 activates promoters of both synthesis (1-SST and 1-FFT) and degradation (1-FEH) genes under stress.”.

* Line 309 - "decomposing" should be changed to something like "metabolism" or "breakdown"
Reply: Corrected.

* Line 316 and the rest of the manuscript - When referring to a specific gene, it should be italicized and all caps. Ex "1-SST"
Reply: Corrected.

* Figure 3A description calls stars, "pentagrams"
Reply: Corrected.

* Figure S16 is very difficult to interpret, even in light of the amino acid alignments

provided. I also feel that it distracts and doesn't add much to overall interpretations. I would encourage authors to consider removing
Reply: We removed Figure S16.

Reviewer #2: The Asteraceae family is the largest angiosperm family, which account for around 10% of all flowering plants, and include many plants employed in medicine, horticulture, ecology, food resources, etc. The authors assembled three chromosome-level genomes of Dahlia pinnata, Cosmos bipinnatus, and Bidens alba in the tribe Coreopsideae. In terms of a series of analyses, they scanned the WGD events, chromosome rearrangements, the potential reasons of genome expansion. Finally, they identified the genes involved in inulin metabolic pathway, and based on the comparison of fructan systhesis genes, they pointed out the possible reasons why D. pinnata produces much more inulin than the other two plants. Overall, the work is sound, especially the quality of three new assembled genomes, and the manuscript is well prepared. However, I still have some major concerns as follows.

First of all, even the author mentioned the reason why they sequenced these three genomes, I was just wondering the importance of these three species in the whole Asteraceae or what is their new insights for asteraceous species study even for angiosperms.
Reply: In the whole Asteraceae, the tribe Coreopsideae stands for one of the 45 recognized tribes. Coreopsideae belongs to the Heliantheae alliance, which includes several important economic species such as sunflower, Jerusalem artichoke, and Smallanthus sonchifolius (yacon). The sequencing of three species in this study may also benefit the breeding of sunflower, Jerusalem artichoke and yacon, by gene editing technologies with gene resources from closely-related species.

Second, it is well known that inulin is a key innovation trait for Asteraceae. To me, the analyses on the of inulin pathway are far away from sufficiency. So far, there are over 20 genomes of asteraceous species available, especially those species like chicory (which has high level inulin). I suggest a more complete genome comparison work on inulin including more asteraceous species even species from outgroup like Scaevola.
Reply: Thank you for this suggestion. We have added more Asteraceae species to study genes for inulin metabolism and regulation, which can be found on Figure 6CDE and Supplementary Figure S11. We added the sentence “By phylogenetic analysis, we inferred the evolutionary history of the inulin genes in related Asteraceae plants, such as Cichorium intybus and Arctium lappa (Supplemental Fig. S11).” to the section of “Pseudogenization in one of the two copies of 1-FEH1 and 1-FEH2 genes in D. pinnata”.

Scaevola taccada is a coastal shrub. a member of Goodeniaceae that is one of the closest outgroups of Asteraceae. There may be no inulin in Scaevola, as no literature reported inulin from Scaevola. Thus, we do not use it as outgroup. However, we add the sentence “In addition to plants in Coreopsideae, further research could use all available Asteraceae plants and plants in closely related outgroup, such as Scaevola taccada in Goodeniaceae [60], to explore why Asteraceae plants take inulin as the primary reserve carbohydrates.” in the Discussion section and cited the following paper: “60.Shen F, Qin YJ, Wang R, Huang X, Wang Y, Gao TA, et al. Comparative genomics reveals a unique nitrogen-carbon balance system in Asteraceae. Nature Communications. 2023;14 1 doi:10.1038/s41467-023-40002-9.”

In addition, only comparing the genes of fructan systhese genes in these three species is not strong enough to make the conclusion that D. pinnata produces much more inulin than the other two plants. Because this is totally based on the well annotation of these genomes (in fact, gene annotation is full of challenge), cloning these genes and RT-PCR or similar methods which can scan these genes expression are highly recommended.
Reply: The genome assembly and gene annotation has been greatly improved with HiFi reads, full length transcript, and so many homology resources. Thus, we think our gene annotations for inulin metabolism genes are of high quality and can be used for downstream analyses.

Powered by Editorial Manager® and ProduXion Manager® from Aries Systems Corporation

	We used the well-studied published genes as the query genes to detect homologous genes in our gene sets and genomes, and manually checked the multiple sequences alignment of the reference and the newly found genes. Although there are mutations, these genes aligned from head to tail.<br><br>Based on full length transcriptome data, we also calculated the expression of inulin genes for C. bipinnatus and B. alba, and compared them with that of D. pinnata (see Supplementary Figure S13). The two synthesis (1-SST and 1-FFT) genes of D. pinnata expressed more than that of C. bipinnatus and B. alba.<br><br>In the end, the main purpose of this project is to finish the genome assembly of these three species and perform genome based evolutionary analyses. By the way, we identified valuable metabolism genes such as inulin, which can be treated as potential gene resources for future studies. Please forgive that more deeper analyses of these functional genes is out of our interest and ability at current stage.
**Additional Information:**	
**Question**	**Response**
Are you submitting this manuscript to a special series or article collection?	No
**Experimental design and statistics**<br><br>Full details of the experimental design and statistical methods used should be given in the Methods section, as detailed in our Minimum Standards Reporting Checklist. Information essential to interpreting the data presented should be made available in the figure legends.<br><br>Have you included all the information requested in your manuscript?	Yes
**Resources**<br><br>A description of all resources used, including antibodies, cell lines, animals and software tools, with enough information to allow them to be uniquely identified, should be included in the Methods section. Authors are strongly encouraged to cite Research Resource Identifiers (RRIDs) for antibodies, model organisms and tools, where possible.<br><br>Have you included the information requested as detailed in our Minimum Standards Reporting Checklist?	Yes

Powered by Editorial Manager® and ProduXion Manager® from Aries Systems Corporation

**Availability of data and materials**<br><br>All datasets and code on which the conclusions of the paper rely must be either included in your submission or deposited in publicly available repositories (where available and ethically appropriate), referencing such data using a unique identifier in the references and in the “Availability of Data and Materials” section of your manuscript.<br><br>Have you have met the above requirement as detailed in our Minimum Standards Reporting Checklist?	Yes

Manuscript

Click here to access/download;Manuscript;renamed_1a963.docx

The genomes of *Dahlia pinnata*, *Cosmos bipinnatus* and *Bidens alba* in tribe Coreopsideae provide insights into polyploid evolution and inulin biosynthesis

Hengchao Wang[#], Dong Xu[#], Fan Jiang, Sen Wang, Anqi Wang, Hangwei Liu, Lihong Lei, Wanqiang Qian, Wei Fan[*]

Guangdong Laboratory for Lingnan Modern Agriculture (Shenzhen Branch), Genome Analysis Laboratory of the Ministry of Agriculture and Rural Affairs, Agricultural Genomics Institute at Shenzhen, Chinese Academy of Agricultural Sciences, Shenzhen, Guangdong 518120, China

[#] Equal contribution.

[*] Corresponding author.

E-mail: fanwei@caas.cn (Fan W)

Hengchao Wang [0000-0002-8754-4195]; Dong Xu [0000-0001-6180-182X]; Fan Jiang [0000-0003-1359-0970]; Sen Wang [0000-0001-9793-4472]; Anqi Wang [0000-0001-9367-2524]; Hangwei Liu [0000-0002-4931-1307]; Lihong Lei [0000-0002-2524-7523]; Wei Fan [0000-0001-5036-8733]

Abstract

Background: The Coreopsideae tribe, a subset of the Asteraceae family, encompasses economically vital genera like Dahlia, Cosmos, and Bidens, which are widely employed in medicine, horticulture, ecology, and food applications. Nevertheless, the lack of reference genomes hinders evolutionary and biological investigations in this tribe.

Results: Here, we present three haplotype-resolved chromosome-level reference genomes of the tribe Coreopsideae, including two popular flowering plants *Dahlia pinnata* and *Cosmos bipinnatus*, and one invasive weed plant *Bidens alba*, with assembled genome sizes 3.93 G, 1.02 G and 1.87 G, respectively. We found that *Gypsy* transposable elements contribute mostly to the larger genome size of *D. pinnata*, and multiple chromosome rearrangements have occurred in tribe Coreopsideae. Besides the shared whole genome duplication (WGD-2) in the Heliantheae alliance, our analyses showed that *D. pinnata* and *B. alba* each underwent an independent recent WGD-3 event: in *D. pinnata* it is more likely to be a self-WGD, while in *B. alba* it is from the hybridization of two ancestor species. Further, we identified key genes in the inulin metabolic pathway, and found that the pseudogenization of *1-FEH1* and *1-FEH2* genes in *D. pinnata* and the deletion of three key residues of 1-FFT proteins in *C. bipinnatus* and *B. alba* may probably explain why *D. pinnata* produces much more inulin than the other two plants.

Conclusions: Collectively, the genomic resources for the Coreopsideae tribe will promote phylogenomics in Asteraceae plants, facilitate ornamental molecular breeding improvements and inulin production, and help prevent invasive weeds.

Keywords: Coreopsideae; *Dahlia pinnata*, *Cosmos bipinnatus*, *Bidens alba*, whole genome duplication, inulin

Introduction

The tribe Coreopsideae in Asteraceae consists of several economically important species in genera Dahlia, Cosmos and Bidens. *Dahlia pinnata* (NCBI:txid101596) is a popular tuberous-rooted ornamental flower crop, widely grown for cut flowers and potted flowers. It is recognized as the national flower of Mexico, and more than 65,500 cultivars of *D. pinnata* have been bred worldwide [1]. *D. pinnata* also contains a lot of inulin and many nutritional compounds in tuberous roots, which can improve a healthy diet [2]. *Cosmos bipinnatus*, commonly known as the garden cosmos or lace cosmos, is popular not only as a garden or a bedding plant but also as cut flowers [3]. *Bidens alba* (NCBI:txid51260) is a worldwide invasive weed that has been listed among the first class of most malignant invasion in the Invasive Alien Species of China (IASC) [4]. Additionally, both *C. bipinnatus* and *B. alba* have been widely used as traditional medicines, due to their anti-inflammatory, anti-oxidative and anti-bacterial activities [5-8].

Comprising about 550 species, Coreopsideae is a tribe of flowering plants within the Heliantheae alliance of the Asteraceae family [9].Coreopsideae consists of a diverse group of plants primarily found in the Americas, with the highest diversity in North America [10]. It was once thought of as the subtribe Coreopsidinae in the tribe Heliantheae according to the morphology taxonomy [11]. Similar to other Asteraceae sub-lineages, many species in this tribe are rich in inulin, a compound widely utilized in food, health and cosmetic industries as sweetener, dietary fiber, viscosity modifier, fat replacer, and prebiotics [12-14]. In addition, most plants in the Asteraceae employed inulin as the primary reserve carbohydrates, instead of starches, which may contribute to strong capability of environmental adaptation for these plants [15-18]. The tubers of *D. pinnata*, chicory and Jerusalem artichoke are currently the main sources of inulin for industrial extraction [19].

Recently, several economic-important species in the Heliantheae alliance have chromosome-scale reference genomes, including *Helianthus annuus* [20], *Ambrosia artemisiifolia* [21], *Stevia rebaudiana* [22], *Mikania micrantha* [23], *Smallanthus sonchifolius* [15]. However, until now, for the tribe Coreopsideae, only the organelle genomes of *D. pinnata*, *C. bipinnatus* and *B. alba*, as well as a highly fragmented contig-level assembly of the hexaploid *Bidens hawaiensis* were available [24-29]. In this work, we present the chromosome-scale haplotype-resolved genomes of *D. pinnata*, *C. bipinnatus* and *B. alba*, to explore the evolution of polyploidization and mechanism under inulin metabolism.

Results

Chromosome-scale haplotype-resolved assemblies of *D. pinnata*, *C. bipinnatus* and *B. alba*

According to previous studies, *D. pinnata* has a 2n=4x=64 karyotype [30-32], and *C. bipinnatus* has a 2n=2x=24 karyotype [30, 33]. However, in the genus Bidens, many plants have a similar morphology and there are tetraploid and hexaploid populations [26, 34]. Previous reports showed that *B. alba* has a 2n=4x=48 karyotype [26, 35], and our karyotype analysis of *B. alba* by fluorescence in situ hybridization (FISH) delivered the same result (Supplemental Fig. S1).

To build high-quality reference genomes, we sequenced *D. pinnata*, *C. bipinnatus*, and *B. alba* with Pacific Biosciences (PacBio) High-Fidelity (HiFi), and long-range Illumina Hi-C technologies (Supplemental Table S1). Using hifiasm [36] with input of 192.5 Gb (48 ×), 78.0 Gb (72 ×), and 93.2 Gb (48 ×) HiFi data as well as 368.0 Gb (92 ×), 113.7 Gb (105 ×), and 163.1 Gb (85 ×) Hi-C data, we obtained contig assembly sizes 7.86 Gb, 1.02 Gb, and 3.75 Gb with contig N50 sizes 29.28 Mb, 52.82 Mb, and 15.05 Mb for *D. pinnata*, *C. bipinnatus*, and *B. alba*, respectively (Table 1). Because of the low heterozygosity rate, the assembly size of *C. bipinnatus* (1.02 Gb) is similar to the estimated haploid genome size (1.08 Gb) by Kmer analysis (Supplemental Fig. S2).

However, due to the high heterozygosity rates and tetraploid karyotype characters, the assembly sizes of *D. pinnata* (7.86 Gb) and *B. alba* (3.75 Gb) are about two times the estimated haploid genome sizes (3.98 Gb of *D. pinnata* and 1.93 Gb of *B. alba*) by Kmer analysis (Supplemental Fig. S2), implying that we acquired both allelic genomes of the two tetraploid plants. Additionally, we downloaded the reference organelle genomes of closely related plants, aligned them with the contig assemblies, and filtered 4.07 Mb, 0.54 Mb and 8.74 Mb organelle sequences for *D. pinnata*, *C. bipinnatus* and *B. alba*, respectively (Supplemental Table S2-S4). Finally, we used YaHS [37] and Juicebox [38] to anchor the contigs onto chromosome-scale scaffolds (Supplemental Table S5-S7), resulting in 95.5%, 93.7%, and 95.2% anchoring rates, with scaffold N50 sizes 118.5 Mb, 79.4 Mb, and 75.1 Mb for *D. pinnata*, *C. bipinnatus*, and *B. alba*, respectively (Figure 1ABC, Table 1 and Supplemental Table S8). For the two tetraploid genomes *D. pinnata* and *B. alba*, strong Hi-C signals formed along with the diagonals within a chromosome and between chromosomes of the same homologous chromosomes group (Supplemental Fig. S3). Moreover, 90 (70.3%), 14 (58.3%) and 55 (57.3%) candidate telomeres were found on the chromosomes of *D. pinnata*, *C. bipinnatus* and *B. alba*, respectively (Supplemental Table S9-S11).

After assembly, we mapped HiFi data to the genomes of two tetraploid plants (*D. pinnata* and *B. alba*) and checked the reads coverage distribution. The results showed that most contigs of both plants have approximately 24 × mean reads coverage (Figure 1D), which is half of the estimated reads coverage for the haploid genome, indicating that we successfully assembled haplotype-resolved genomes. Besides, the BUSCO complete rates of the genomes are 99.1%, 97.4%, and 99.6% for *D. pinnata*, *C. bipinnatus*, and *B. alba*, respectively, which are higher or comparable to those of related published species (Supplemental Table S12).

Integrating evidence from full-length transcriptome (Iso-seq), short reads transcriptome (RNA-seq) and homologous proteins, Augustus [39] predicted 181,915, 46,076 and 165,431 protein-coding genes for *D. pinnata*, *C. bipinnatus* and *B. alba*, respectively

(Supplemental Fig. S4, Supplemental Table S13-S17). The BUSCO complete rates of the gene sets were 98.9%, 96.7% and 98.4% for *D. pinnata*, *C. bipinnatus* and *B. alba*, respectively (Supplemental Table S18). For the two tetraploid plants, we also compared the BUSCO completeness of genes from one haplotype (one subgenome) and two haplotypes. The results showed that the complete and duplicated rates of two haplotypes were both higher than that of one haplotype (Figure 1E). For gene functional annotation, 173,686 (95.48%) of *D. pinnata*, 44,074 (95.66%) of *C. bipinnatus* and 151,025 (91.29%) of *B. alba* genes were annotated by at least one of NR, KEGG, Uniprot (Swiss-Prot) and InterPro databases (Supplemental Table S19). Based on the gene sets, we predicted transcription factors (TFs) by PlantTFDB [40], and the most abundant TF families were bHLH, ERF, MYB and C2H2 (Supplemental Table S20). Moreover, we also predicted tRNAs, rRNAs and ncRNAs (Supplemental Table S21 and S22).

Recent expansion of *Gypsy* LTRs enlarges the genome of *D. pinnata*

In comparison to *C. bipinnatus* and *B. alba*, *D. pinnata* has a much bigger genome size. Apart from the effect of whole genome duplications, the genome sizes of one haplotype were about 1.99 Gb, 1.08 Gb and 0.97 Gb for *D. pinnata*, *C. bipinnatus* and *B. alba*, respectively. Notably, 1.72 Gb (86%) of *D. pinnata*, 0.82 Gb (76%) of *C. bipinnatus* and 0.73 Gb (75%) of *B. alba* were TEs (Table 1), implying that TEs expansion is the major force that enlarges the genome of *D. pinnata*. Furthermore, we compared the abundances of different TE classes and found that LTRs were the most abundant TEs for all three plants (Supplemental Table S23). Among the LTRs, the *Gypsy* TEs were most abundant (Figure 2A). In addition to the quantities, we also estimated the insertion time of different subclasses of LTRs. These LTR TEs were mainly expanded within the last 1 million years (Figure 2B and Supplemental Fig. S5), which is similar to that of sunflower [20]. Considering that *Gypsy* LTRs were the dominant LTRs and the recent insertion peak of *Gypsy* LTRs was much higher than that of the *Copia* and other LTRs, we speculate that the recent expansion of *Gypsy* LTRs contributed mostly to the larger genome size of *D. pinnata*.

Multiple chromosome rearrangements experienced in tribe Coreopsideae

To resolve the phylogenetic relationships of plants in the tribe Coreopsideae, we performed evolutionary analyses using the gene sets of 12 Asteraceae plants, including *D. pinnata*, *C. bipinnata*, *B. alba*, *Ambrosia artemisiifolia* [21], *Helianthus annuus* [20], *Stevia rebaudiana* [22], *Mikania micrantha* [23], *Smallanthus sonchifolius* [15], *Chrysanthemum nankingense* [41], *Artemisia annua* [42], *Conyza canadensis* [43] and *Arctium lappa* [15], with *Vitis vinifera* as outgroup (Supplemental Table S17 and S24). In total, OrthoFinder assigned 681,319 genes (93.8% of the total) to 40,991 orthogroups [44], and there were 9,121 orthogroups with all species present (Supplemental Table S25). Using 354 orthogroups with a minimum of 46.2% of species having single-copy genes, OrthoFinder called STAG to build a species tree and STRIDE to root this tree (Supplemental Fig. S6). On the other hand, we built a phylogenetic tree using RAxML-NG with the data of 274 single-copy genes for each species (Figure 3A). The two species tree had the same topology and very similar sequence evolution rates (branch lengths), demonstrating that the species tree is credible.

The relationships of the three genera Dahlia, Cosmos and Bidens in the tribe Coreopsideae have been in controversy. Previous phylotranscriptomic analyses supported that the genera Dahlia and Cosmos are sisters, and the genus Bidens is a sister of the last common ancestor of the genera Dahlia and Cosmos [45]. However, other studies by internal transcribed spacer (ITS) data [46] and plastid DNA sequences [10] supported that the genus Cosmos is closer to the genus Bidens, and the genus Dahlia is sister with the last common ancestor of the genera Cosmos and Bidens. Our study with the genome-wide data showed that Cosmos and Bidens are closer than Dahlia (Figure 3A), which is consistent with the studies from ITS and plastid data. Using the molecular clock dating method, we estimated the divergence times among these plants (Figure 3A). In the Heliantheae alliance, the tribe Coreopsideae and the tribe Heliantheae separated 17.48 million years ago (Mya). *D. pinnata* diverged from the last common ancestor of *C. bipinnata* and *B. alba* 14.62 Mya, while *C. bipinnata* and *B. alba* diverged from each other 7.00 Mya. Notably, *B. alba* mutated faster than *C. bipinnatus*,

D. pinnata, and other related plants in Asteraceae. As a notorious invasive weed, the fast mutation rate may contribute to the strong invasive capabilities of *B. alba*.

With the chromosome-scale haplotype-resolved assemblies of *D. pinnata*, *C. bipinnata* and *B. alba*, we studied chromosome conservation and rearrangement among these plants (Figure 3B). From the macro-synteny plot, we showed that only one chromosome group, including the Chr 7 of *D. pinnata*, the Chr 4 of *C. bipinnata* and the Chr 4 of *B. alba*, preserved chromosome-scale collinearity among the three species. Meanwhile, there were three chromosome pairs preserved chromosome-scale collinearity between two species, including Chr 5 of *D. pinnata* and Chr 5 of *C. bipinnata*, Chr 2 of *C. bipinnata* and Chr 8 of *B. alba*, as well as Chr 12 of *C. bipinnata* and Chr 1 of *B. alba*. Further, the other chromosomes of the three plants experienced chromosome fusion and fission events after the last common ancestor of the tribe Coreopsideae.

Genomic evidence supports *D. pinnata* to be autotetraploid

The ploidy of *D. pinnata* has been in controversy. Some scholars proposed *D. pinnata* is an allooctoploid species without evidence [47] and based on chromosome pairing patterns [48]. Using simple-sequence repeat (SSR) and amplified fragment length polymorphism (AFLP) data, Stephan and colleagues indicated that *D. pinnata* has an autooctoploid genome [31]. However, another study suggested *D. pinnata* to be a tetraploid species by comparing chromosome numbers of the close relatives [49]. Based on the chromosome-scale haplotype-resolved assembly, we observed strong head-to-tail collinearity among the four haplotypes in each of the 16 homologous chromosome groups, without any translocation between any two chromosome groups (Figure 4A and Supplemental Fig. S7). These results implied that *D. pinnata* has a tetraploid genome architecture, which is derived from a recent whole genome duplication.

To determine the time of the recent whole genome duplication in *D. pinnata*, we analyzed the distribution of synonymous substitution rate (*Ks*) of homologous gene pairs for one haplotype (subgenome) and two haplotypes of *D. pinnata*, *C. bipinnata*,

H. annuus. In comparison, we also analyzed the distribution of *Ks* between one haplotype of *D. pinnata* and *C. bipinnata*(Figure 4B). For *D. pinnata*, the *Ks* peaks at about 1.4 and 0.5 correspond to WGT-1 and WGD-2, respectively, which are shared in the lineage of the Heliantheae alliance [15]. Besides, *D. pinnata* has a specific *Ks* peak at ~0.03, which is responsible for the recent whole genome duplication (WGD-3). Given that the *Ks* peak corresponding to the divergence between *D. pinnata* and *C. bipinnatus* is ~0.35, and this divergence occurred ~14.62 Mya (Figure 3A), we estimated that the WGD-3 event of *D. pinnata* occurred ~1.25 Mya. Thus, the time of the WGD-3 event was very close to the current, which explains the strong macro-synteny among chromosome haplotypes.

To further study the polyploid history of *D. pinnata*, we constructed the phylogenetic tree of each homologous chromosome group taking each chromosome as a virtual species by the single-copy gene concatenation method. In total, there were 9 homologous groups with ((A1, A2), (A3, A4)) topology and 7 homologous groups with (((A1, A2), A3), A4) topology (Figure 4C). Considering that the WGD-3 of *D. pinnata* occurred just ~1.25 Mya, and *D. pinnata* has the lowest mutation rate among the three plants of the tribe Coreopsideae (Figure 3A), the sequence difference between the duplicated chromosome pairs derived from WGD-3 is still not large enough to distinguish from the sequence difference from the two allelic chromosomes which have high heterozygosity. Additionally, we attempted to phase subgenomes of *D. pinnata* based on subgenome-specific k-mers, resulting in that there were very few subgenome-specific k-mers and thus we failed to phase the subgenomes, due to the very recent LTR insertion (Supplemental Fig. S8). Therefore, it is difficult to get the ((A1, A2), (A3, A4)) topology for all the homologous chromosome groups, and impossible to clearly distinguish the subgenomes with allelic genomes from current data. Taking together all the evidence, we concluded that the ploidy of *D. pinnata* is more likely to be an autotetraploid, which originated from WGD-3 of a single ancestor species.

Genomic evidence reveal *B. alba* as an allotetraploid

To study the evolution history of chromosomes in *B. alba*, we showed the macro-synteny for the 12 groups of homologous chromosomes (Figure 5A and Supplemental Fig. S9). Among the 12 groups, 9 groups (1, 3, 4, 7, 8, 9, 10, 11 and 12) demonstrated holistic chromosome collinearity for the four haplotypes, which was similar to that of *D. pinnata*. In the remaining three groups, holistic macro-synteny exists only in two pairs of haplotypes but not among all four haplotypes, and large fragment translocations occurred among these three homologous chromosome groups. These results suggested that *B. alba* has a tetraploid genome architecture, which is derived from a whole genome duplication, possibly by the hybridization of two ancestor species.

To estimate the time of hybridization event of *B. alba*, we analyzed the distribution of synonymous substitution rate (*Ks*) of homologous gene pairs for 1 haplotype and 2 haplotypes of *B. alba*, *C. bipinnata*, *H. annuus*, and between 1 haplotype of *B. alba* and *C. bipinnata* (Figure 5B). Since the mutation rate of *B. alba* is faster than that of the other plants analyzed (Figure 3A), the *Ks* peaks for WGD-2 and WGT-1 were both right-shifted compared with that of *H. annuus*. In addition, *B. alba* has a specific *Ks* peak at ~0.08, which is caused by the recent whole genome duplication (WGD-3), i.e. the hybridization event. Considering that the Ks peak corresponding to the divergence between *B. alba* and *C. bipinnatus* is ~0.23, and the divergence of these two species occurred at ~7.00 Mya (Figure 3A), the time of the WGD-3 of *B. alba* was estimated to be ~2.43 Mya. Note that the real time for the hybridization event may be a little later than 2.43 Mya, because the divergence time estimation used both the accumulated mutations since the hybridization event and the original sequence divergence of the two ancestor species.

Using the single-copy gene concatenation method, we constructed the phylogenetic trees for the homologous chromosome groups taking each chromosome as a virtual species. For all the 12 homologous groups, the trees showed the same topology ((A1, A2),(B1, B2)) (Figure 5C), indicating that the four chromosome haplotypes could be separated into two pairs, with each representing a subgenome. On the other hand, we

also attempted to phase subgenomes of *B. alba* based on subgenome-specific k-mers, resulting in that there were very few subgenome-specific k-mers and thus we failed to phase the subgenomes, due to the very recent LTR insertion (Supplemental Fig. S10). Taking together both the macro-synteny and the phylogenetic topology evidence, we concluded that the ploidy of *B. alba* is more likely to be an allotetraploid, which originated from WGD-3 by the hybridization of two ancestor species.

Pseudogenization in one of the two copies of 1-FEH1 and 1-FEH2 genes in *D. pinnata*

The tubers of *D. pinnata* encompass large quantities of inulin and have been used as one of the main sources of industry inulin production [12, 19]. There are four key genes involved in the metabolism of inulin. The enzyme sucrose: sucrose 1-fructosyl transferase (1-SST) catalyzes two sucrose molecules to produce 1-kestose and glucose. Then, the enzyme fructan: fructan 1-fructosyl transferase (1-FFT) further elongates linear inulin-type fructans with β(2,1) fructosyl units, taking 1-kestose as the preferential donor. Breakdown of inulin is performed by the action of fructan 1-exohydrolase (1-FEH1 and 1-FEH2) which hydrolyzes terminal fructoses [50]. In addition to these functional genes, previous studies also reported in chicory, MYB3 and MYB5 can enhance promoter activities of *1-FEH* genes in response to abiotic stress and hormonal cues, while MYB17 activates promoters of both synthesis (*1-SST* and *1-FFT*) and degradation (*1-FEH*) genes under stress. [51, 52]. However, the key genes of *D. pinnata* for inulin biosynthesis and breakdown still lack investigation.

Based on homology alignment to known genes, we identified the key genes of functional enzymes and transcription factors for *D. pinnata*, *C. bipinnatus* and *B. alba* (Figure 6A, Supplemental Table S26). By phylogenetic analysis, we inferred the evolutionary history of the inulin genes in related Asteraceae plants, such as *Cichorium intybus* and *Arctium lappa* (Supplemental Fig. S11). In addition, we used the publicly available RNA-seq dataset of *D. pinnata* and full length transcriptome of *C. bipinnatus* and *B. alba* to validate these genes (Supplemental Fig. S12 and Fig. S13), and found

that the two synthesis genes (*1-SST* and *1-FFT*) expressed more in *D. pinnata* that the other two plants, which is consistent with the fact that *D. pinnata* produces much more inulin than *C. bipinnatus* and *B. alba*. For the two biosynthesis genes *1-SST* and *1-FFT*, only one copy was found in *C. bipinnatus* and two copies were discovered in *D. pinnata* and *B. alba* due to the recent WGDs. Meanwhile, the *1-SST* and *1-FFT* genes formed a biosynthetic gene cluster (BGC) in *D. pinnata* and *C. bipinnatus* (Figure 6B), which may contribute to inulin production in the two species [53]. However, the *1-SST* and *1-FFT* genes are located on different chromosomes (Chr 10 and Chr 11) in *B. alba*, and there are five pseudogenized *1-FFT* genes near the *1-SST* genes (Figure 6B and Supplemental Fig. S14). Only some short fragmented micro-synteny were detected between Chr10 and Chr11, and the *1-SST* and *1-FFT* genes located in one of these syntenic fragments that likely to be the relics of WGD-2 (Supplemental Fig. S15). On the other hand, both *D. pinnata* and *B. alba* have two copies of breakdown genes *1-FEH1* and *1-FEH2*, which were derived from their recent species-specific whole genome duplications. However, one copy of *1-FEH1* and one copy of *1-FEH2* have become pseudogene in *D. pinnata* (Figure 6C, 6D, Supplemental Table S27, Fig. S16 and S17). The pseudogenization of *1-FEH1* and *1-FEH2* genes may decrease the breakdown of inulin, thus preserving more inulin in the tuber for *D. pinnata*.

Missing three key residues “DPN” in 1-FFT proteins of *C. bipinnatus* and *B. alba*

As 1-FFT is critical for the inulin polymerization, we compared the difference of the 1-FFT protein for the three plants of the tribe Coreopsideae. Firstly, we aligned the protein sequences of these *1-FFT* genes together (Supplemental Fig. S18). According to the resulting multiple sequence alignment and the knowledge from previous studies [54], we determined the catalytic triads for these proteins (Figure 6E). The g6466 of *C. bipinnatus* and the g141421 of *B. alba* missed three key amino acids “DPN” in the first motif of the catalytic triad, which could impact the function of these proteins.

Furthermore, we predicted the 3D structures of the 1-FFT proteins by AlphaFold [55]. Then, through a superposition of these protein structures, we found that all of them have

very similar structures, containing an N-terminal β-propeller domain, a C-terminal β-sheet domain and a cleft between the two domains. For the catalytic triads, the loss of three residues greatly changed the conformation of 1-FFT (Figure 6E). Importantly, the first residue “D” in the missing “DPN” has been reported acting as the nucleophile and being involved in the catalyzing process [56]. All plant fructan-metabolizing enzymes belong to the glycoside hydrolase 32 (GH32) family and the residues at the catalytic triads are conserved among these enzymes [57]. Hence, the loss of the three residues in 1-FFTs of *C. bipinnatus* and *B. alba* may severely influence their inulin polymerization function.

Discussion

In this study, we present the high-quality reference genomes of three important plants *D. pinnata*, *C. bipinnatus* and *B. alba* in the tribe Coreopsideae of Asteraceae. Thanks to the long and highly accurate PacBio HiFi data and long-range Hi-C data, we achieved the chromosome-scale haplotype-resolved assemblies. To get the final telomere-to-telomere reference genomes for these plants, Oxford Nanopore Technologies (ONT) ultra-long reads or other advanced technologies may be helpful. With the comprehensive annotation of TEs, we found that LTRs especially *Gypsy* were the most abundant LTRs in *D. pinnata*, which were significantly expanded within the last 1 million years and was largely responsible for the larger genome size of *D. pinnata*. Because TEs can modify regulatory networks and create new genes [58], the recent expansion of the *Gypsy* TEs may also contribute to the wide diversity of cultivars of *D. pinnata*.

Phylogenomic analysis clarified that the genera Bidens and Cosmos are close sisters, which diverged from each other ~7.00 Mya. The genus Dahlia separated from the last common ancestor of the genera Bidens and Cosmos 14.62 Mya. With haplotype-resolved chromosomes, we revealed that *D. pinnata* is an autotetraploid plant derived from a recent whole genome duplication. A previous study using genomic in situ

hybridization (GISH) has shown that meiosis chromosome pairing occurs both between and within parental genomes [59], supporting our conclusion. We also showed that *B. alba* is an allotetraploid, which originated from the hybridization of two ancestor species followed by chromosome doubling. The hybridization event was relatively far from the current, so *B. alba* exhibited diploid inheritance features in the meiosis process [34, 60].

Compared with *C. bipinnatus* and *B. alba,* the tubers of *D. pinnata* are much larger and composed of a high proportion of inulin-type fructan. Within the genomes, we identified all copies of the key functional genes and transcription factors for inulin biosynthesis and degradation. We found one of the two copies of *1-FEH1* and *1-FEH2* genes in *D. pinnata* became pseudogenes, which may decrease the breakdown of inulin and thus increase the inulin accumulation in the tuber of *D. pinnata*. The pseudogenization of *1-FEH1* and *1-FEH2* genes may also influence the degree of inulin polymerization. On the other hand, the two copies of *1-FFT* genes in *D. pinnata* preserved canonical residues in the catalytic triads, while the 1-FFT of *C. bipinnatus* and one of the two copies of 1-FFT in *B. alba* lost three residues in the first motif of the catalytic triads, which may severely influence the inulin biosynthesis and probably explain why the efficiency of inulin production of *C. bipinnatus* and *B. alba* are much lower than that of *D. pinnata*. In addition to plants in Coreopsideae, further research could use all available Asteraceae plants and plants in closely related outgroup, such as Scaevola taccada in Goodeniaceae [61], to explore why Asteraceae plants take inulin as the primary reserve carbohydrates.

D. pinnata and *C. bipinnatus* are popular flowers all over the world [3, 62, 63], and *B. alba* is a traditional medical plant although it is also recognized as an invasive plant [60]. The genomic resources of *D. pinnata* and *C. bipinnatus* presented in this study will promote the studies on regulating mechanisms of flower shapes, colors and senescence, and benefit the flower industry by breeding more popular and long-vase

life cultivars of flowers. In addition, the genomic resources of *B. alba* will help develop more efficient and green methods to prevent the invasion of this weed.

Methods

Plant materials, DNA, and RNA sequencing

The tubers of the *Dahlia pinnata* cultivar 'Kelvin floodlight' were purchased from a horticultural company located in Chuzhou, Anhui province, China. The seeds of *Cosmos bipinnatus* were acquired from a seed company located in Shuyang, Jiangsu province. The seeds of *Bidens alba* were collected from the riverside in Shenzhen, Guangdong province. The tubers and seeds were grown on the farm of the Agricultural Genomics Institute at Shenzhen, Chinese Academy of Agricultural Sciences. For *B. alba*, fresh tips of plant roots were collected and analyzed using fluorescence in situ hybridization (FISH) for karyotype analysis.

Tender fresh leaves were collected from one plant per species. 50-days-old *D. pinnata*, 50-days-old *C. bipinnatus* and 40-days-old *B. alba* were used for sequencing. For PacBio HiFi sequencing (Pacific Biosciences, California, USA), the high-quality genomic DNA was extracted using the Qiagen DNeasy Plant Mini Kit (Qiagen, Hilden, Germany). The quality of DNA was assessed using 0.75% agarose gel electrophoresis, Nanodrop and Qubit fluorimeter (Thermo Fisher, Massachusetts, USA). SMRTbellTM Express Template Prep Kit 2.0 was used to create a 20-kilobase DNA SMRTbell library. The library was sequenced using the PacBio Sequel II platform. For genomic DNBSEQ sequencing of *D. pinnata*, a library with 500 bp insert length was prepared without using polymerase chain reaction (PCR) by following standard protocols. Paired-end reads (PE150) were sequenced on DNBSEQ-T7 (RRID:SCR_017981). For Hi–C sequencing, the fresh leaves were shredded and cross-linked with 2% formaldehyde. The DNA was then digested DNA using the MboI enzyme, followed by biotin labeling the ends of fragments. Fragmented DNA was ligated and sheared. The

biotin-labeled fragments were enriched with streptavidin beads, and used to build a sequencing library. This library was sequenced on Illumina HiSeq 2500 (RRID:SCR_016383) by PE150 bp (Illumina, San Diego, USA). Moreover, for full-length transcriptome sequencing (PacBio Iso-Seq), high-quality RNA was extracted by Qiagen kit from root, stem, leaf and flower tissues per species. The standard protocol was used to construct a sequencing library with an insertion size of 0.5–6 kb. This library was sequenced on the PacBio Sequel II platform (RRID:SCR_017990).

Genome assembly

To estimate genome size, we used Kmerfreq [64] to count K-mer frequency based on DNBSEQ reads (for *D. pinnata*) and HiFi reads (for *C. bipinnatus* and *B. alba*). Genome size estimation was accomplished using the concept of GCE (RRID:SCR_017332) [65].

For contigs assembly, we used hifiasm (RRID:SCR_021069) v0.18.5 to assemble contigs by integrating HiFi and Hi-C sequencing reads per species [36]. Because *D. pinnata* and *B. alba* are tetraploid plants, and hifiasm is designed to assemble a diploid genome, the resulting haplotype 1 and haplotype 2 were not properly separated. Thus, we merged two haplotypes, and the merged assembly contained both subgenomes and allelic genomes. For *C. bipinnatus*, a diploid plant, one haplotype can be taken as the reference assembly. Due to the heterozygous character of *C. bipinnatus*, we used purge_dups (RRID:SCR_021173) v1.2.6 with parameter settings "-a 0.70" and "-f 0.65" to remove haplotypic duplications [66].

To filter organelle genomes, the chloroplast and mitochondrion genomes of Coreopsideae plants were downloaded from the NCBI database. We used minimap2 (RRID:SCR_018550) v2.24 with parameters "-cx asm20" to align the contigs of *D. pinnata*, *C. bipinnatus* and *B. alba* onto the reference organelle genomes of Coreopsideae plants [67]. Contigs that are composed of more than 95% organelle sequence and less than 5% divergence were filtered to get the nucleus genomes. The

completeness of the contig assemblies was evaluated by BUSCO (RRID:SCR_015008) v5.4.4 with the embryophyta lineage database of OrthoDB (RRID:SCR_011980) v10 [68].

To ensure the contigs of two tetraploid genomes were not merged contigs, that is, similar sequences from subgenomes or allelic genomes were merged into one contigs in the assembly, we checked the average coverage of contigs based on HiFi data. Firstly, we used minimap2 with parameters "-ax map-hifi -I 16G" to map HiFi reads onto the contigs. Then, we used samtools (RRID:SCR_002105) v1.9 with parameters "-F 0x104" to filter unmapped records and non-primary alignments; we used bedtools (RRID:SCR_006646) v2.30.0 with parameters "genomecov -ibam -bg" to calculate the coverage of contigs. Taking the resulting bedGraph files as input, an in-house Perl script was used to get the average coverage of each contig.

As suggested by YaHS (RRID:SCR_022965), the state-of-the-art Hi-C scaffolding tool, we used the Arima Genomics' mapping pipeline[69] to process Hi-C reads for each species. The pipeline mapped reads in single-end read mode, filtered chimeric joined reads, paired reads, and removed PCR duplications. After that, we used YaHS v1.2a.1 with parameters "--no-contig-ec -e GATC" to construct chromosome-scale scaffolds based on the above clean Hi-C mapping results [37]. For tetraploid plants *D. pinnata* and *B. alba*, the contig assembly contained four haplotypes, and YaHS could group contigs from four homologous chromosomes together. Finally, based on the Hi-C signal features and the telomeres information, we manually curated the scaffolds on Juicebox (RRID:SCR_021172) [38]. The chromosomes of tetraploid plants from the same homologous chromosome group would form strong diagonal Hi-C interaction not only within a chromosome but also between chromosomes. This feature is very helpful for manual curation.

Repeats analysis

For tandem repeats, we utilized Tandem Repeats Finder ((RRID:SCR_022193)) v4.09 to identify tandem repeats [70]. We also identified candidate telomeres on the scaffolds based on the TRF results and telomere sequence of ‘CCCTAAA’ and ‘TTTAGGG’. For transposable elements (TEs), three steps were used to find them: (1) structure-intact TEs (including LTR-RT, DNA transposon, Helitron) were predicted by Extensive *de-novo* TE Annotator (EDTA) v1.9.9 and simultaneously the intact TE library was generated [71]; (2) incomplete TEs were recognized by homology-searching against the above intact TE library, Repbase database (RRID:SCR_021169) v26.05 (plant lineage) with RepeatMasker (RRID:SCR_012954), and protein-coding TE database with RepeatProteinMask v4.1.2; (3) a de novo TE library was built based on the masked genome with identified TEs (all above intact and incomplete TEs) using RepeatModeler (RRID:SCR_015027) v2.0.2, and transposable elements representation learner (TERL) was used to classify the TE sequences in the library [72]. The TE sequences that could be classified as any of the TE classes were used by RepeatMasker to identify the remaining TEs in the genome. Finally, we merged all TE annotations, removed redundancy, and got the final TE annotation.

For estimation of LTR insertion times, we extracted sequences of LTRs based on intact TEs annotated by EDTA and used MUSCLE to align long terminal repeats of each LTR. The pairwise distances from the LTRs were estimated by the APE package in R with the K80 model. The sequence divergence (d) of long terminal repeats of each LTR and the mutation rate of sunflower ($r = 1\times10^{-8}$) were used to calculate the times with the equation $T = d/2r$ [20].

Gene prediction

To predict protein-coding genes, we used Augustus with hints on gene structures, such as intron, exon, part of exon, part of CDS and gene start, derived from PacBio Iso-seq, RNA-seq and protein homology [73]. For Iso-seq, we used lima with parameters “--isoseq --peek-guess” to clip the primer sequence. Then, isoseq3 (RRID:SCR_022749) v3.4.0 was used to refine and cluster CCS reads to get high-quality transcripts. We used

GMAP (RRID:SCR_008992) v2020-10-27 to map the high-quality full-length transcripts to assemblies and the result files were filtered and transferred into hints file by blat2hints.pl in AUGUSTUS (RRID:SCR_008417) [39]. For homology searches, we used miniprot v0.10 with parameters "-ut50 --gtf -I" to map proteins of *Helianthus annuus*, *Smallanthus sonchifolius* and *Stevia rebaudiana* to the genomes [74]. After that, we used aln2hints.pl in GALBA v2022-11-07 with parameters "--prg=miniprot" to filter the resulting alignment files and transfer them into hints files[75]. For Illumina RNA-seq analysis, datasets of *D. pinnata* and *C. bipinnatus* were downloaded from NCBI, and STAR (RRID:SCR_004463) v2.7.10 was used to map reads to the genomes. The result bam files were filtered by filterBam in Augustus with parameters "--uniq --paired --pairwiseAlignments". After sorting by samtools, bam2hints in Augustus was used to generate hints files with parameters "--intronsonly". After comparison of the training parameters derived from BUSCO assessment with eudicots_odb10 and embryophyta_odb10 lineage databases, we chose the parameter files generated with eudicots_odb10 as the species-specific gene prediction models, because we can get more complete gene sets [68]. Based on these gene prediction parameters, integrating hints from Iso-seq, homology search, RNA-seq and TE soft-masked genomes, Augustus v3.5.0 was used with parameters "--hintsfile=hintsfile.gff --gff3=on --softmasking=on --codingseq=on --noInFrameStop=true" to predict transcripts of genes.

Functional annotation

To assign gene functions for the gene sets, we used InterProScan (RRID:SCR_005829) v5.52-86 to annotate genes and got Gene Ontology (GO) terms [76]. We also aligned the protein sequences of genes with KEGG, NR and Swiss-Prot databases by Diamond, an alternative replacement of blast, using 1e−5 as a cutoff and got the best hit. We used PlantTFDB (RRID:SCR_003362) v5.0 to annotate transcription factors [40]. Moreover, tRNAscan-SE (RRID:SCR_008637) v2.0 was used to find transfer RNAs (tRNAs) with default parameters [77] and cmscan from infernal (RRID:SCR_011809) v1.1.4 [78] was used to find other non-coding RNAs (ncRNAs) based on Rfam (RRID:SCR_007891) v14 [79]. RNAmmer (RRID:SCR_017075) v1.2 [80] with

parameters "-S euk -m lsu,ssu,tsu" was used to annotate 8S, 18S and 28S ribosomal RNA (rRNA).

Evolution analysis

For orthogroup construction, we used datasets of 13 species, including *D. pinnata*, *C. bipinnata*, *B. alba*, *Ambrosia artemisiifolia* [21], *Helianthus annuus* [20], *Stevia rebaudiana* [22], *Mikania micrantha* [23], *Smallanthus sonchifolius* [15], *Chrysanthemum nankingense* [41], *Artemisia annua* [42], *Conyza canadensis* [43], *Arctium lappa* [15], *Vitis vinifera* (outgroup). We used OrthoFinder (RRID:SCR_017118) v2.5.5 with parameters '-M msa -A mafft -T fasttree -1 -y -S diamond_ultra_sens' to build orthogroups [44]. For *D. pinnata* and *B. alba*, genes on two haplotypes (A1 and A3 for *D. pinnata*; A1 and B1 for *B. alba*) were employed. OrthoFinder called Species Tree from All Genes (STAG) to build an unrooted species tree [81] and used Species Tree Root Inference from Gene Duplication Events (STRIDE) to root this tree [82]. In addition to this species tree, we also constructed a new species tree based on the traditional single-copy genes concatenation method. Firstly, we identified single-copy genes across species according to the results of phylogenetic hierarchical orthogroups from OrthoFinder. Because *D. pinnata, B. alba* and *S. sonchifolius* are tetraploids, we randomly chose one gene from duplicated genes. Secondly, we used MUSCLE (RRID:SCR_011812) v3.8.31 to do multiple sequence alignment [83] and employed RAxML-NG to construct a species tree with parameters '--all --model GTR+G --tree pars 10 --bs-trees 100 --outgroup Vitis_vinifera' using *V. vinifera* as outgroup [84].

To build a time tree, we used MEGA-CC with calibration time 36.6–45.1 Mya between *H. annuus* and *A. lappa* from TimeTree (RRID:SCR_021162), 8 gamma distribution and general time reversible model according to the multiple sequence alignment (MSA) and the species tree [85]. To detect syntenic gene blocks, we employed MCScanX (RRID:SCR_022067) to find collinear genes [86]. Based on the collinear genes, KaKs_Calculator (RRID:SCR_022068) v2.0 with parameters '-m GMYN' was used to

estimate synonymous substitution rate (*Ks*) for orthologue genes [87]. Circos was used to plot circos plot [88]. For *D. pinnata* and *B. alba*, we also employed JCVI [89] to detect collinearity between different haplotypes. The phylogenetic relationships of chromosomes within each homologous chromosome group were inferred based on the single-copy gene concatenation method. We also employed SubPhaser to phase subgenomes by based on subgenome-specific k-mers [90].

Genome mining for inulin metabolism and regulation genes

To identify the inulin metabolism genes, we collected the sequences of published genes in Asteraceae species involved in the synthesis and degradation of inulin. In total, three 1-SST (APM87555.1, AFB83198.1, CAA08812.1), four 1-FFT (APM87557.1, QJQ28876.1, AAD00558.1, CAA04120.2), two 1-FEH1 (AJW31155.1, CAC19366.1) and five 1-FEH2 (APM87560.1, BAL73222.1, AAP85536.1, AIP90173.1, AJW31156.1) genes were collected and checked in multiple sequence alignments. Then, we collected the published transcription factor genes (CiMYB3: ARO49672.1, CiMYB5: ARO49674.1, CiMYB17: ARO49669.1) from chicory that have been reported to play key roles in inulin metabolism regulation [51, 52]. These genes were aligned to the reference gene sets of *D. pinnata*, *C. bipinnatus* and *B. alba*, by Blast (RRID:SCR_004870) v2.3.0 with parameters "blastp -task blastp -evalue 1e-5". The genes with sequence similarity over 75% and length coverage over 75% were taken as potential inulin metabolism genes in each species. For confirmation, the orthogroups that contained these genes were obtained and checked. Finally, possible pseudo-genes or missed genes were identified through Exonerate (RRID:SCR_016088) v2.4.0 by aligning the collected public genes to the reference genomes [91]. Then, we used MUSCLE to do MSA with the candidate genes, and pyBoxshade [92] to show the MSAs. The gene tree of each gene family was built by RAxML-NG with parameters "--all --model GTR+G --tree pars 20 --bs-trees 1000" based on MSAs of CDS, and the tree figures were plotted by FigTree (RRID:SCR_008515) v1.4.4.

To evaluate the gene expression in *D. pinnata*, we used publicly available RNA-seq datasets and employed STAR v2.7.1a to map them to the reference genes. In the PRJNA811758, there are 3 tissues (leaf, stem and root) with each 3 biological repeats in the normal growing state. The reads count of each gene was acquired from the mapping results, and our in-house Perl program was used to calculate Transcripts Per Millions (TPMs). For gene expression of C. bipinnatus and B. alba, we used minimap2 to align the full length transcriptome data to the gene sets and used in-house Perl program to calculate TPMs. For the gene expression heatmap, we used the pheatmap and RColorBrewer packages of R v.4.2.0 to draw figures based on the TPM. Additionally, the 3D structure for 1-FFT genes was predicted by Alphafold v2.1.0 [55], and was demonstrated by open-source PyMOL (RRID:SCR_000305) v2.5.0.

Data availability

All the whole-genome sequencing data that support this project have been deposited at NCBI with BioProject ID: PRJNA1055312, and China National Genomics Data Center with the Project ID PRJCA017572. The genome assemblies, gene annotations, and other resources are available at Zenodo [93] and AGIS website [94] Customized codes used in this project can be found on GitHub [95]. All additional supporting data are available in the *GigaScience* repository, GigaDB [96].

Additional files

Supplementary Table S1. Sequencing statistics of *D. pinnata*, *B. alba* and *C. bipinnatus*.

Supplementary Table S2. Statistics of reference organelle genomes used for filtering sequence from organelle.

Supplementary Table S3. Statistics of filtered organelle genome sequence.

Supplementary Table S4. Statistics of contig assemblies for *D. pinnata*, *C. bipinnatus* and *B. alba*.

Supplementary Table S5. Statistics of Hi-C reads mapping for *D. pinnata*.

Supplementary Table S6. Statistics of Hi-C reads mapping for *C. bipinnatus*.

Supplementary Table S7. Statistics of Hi-C reads mapping for *B. alba*.

Supplementary Table S8. Statistics of scaffold assemblies for *D. pinnata*, *C. bipinnatus* and *B. alba*.

Supplementary Table S9. Positions of telomeres on the chromosomes of *D. pinnata*.

Supplementary Table S10. Positions of telomeres on the chromosomes of *C. bipinnatus*.

Supplementary Table S11. Positions of telomeres on the chromosomes of *B. alba*.

Supplementary Table S12. BUSCO assessment of the genomes of *D. pinnata*, *C. bipinnatus*, *B. alba* and other related species.

Supplementary Table S13. Public RNA-seq datasets statistics.

Supplementary Table S14. Statistics of gene prediction evidence from full-length cDNA mapping.

Supplementary Table S15. Statistics of gene prediction evidence from homology alignment.

Supplementary Table S16. Statistics of gene prediction evidence from RNA-seq mapping.

Supplementary Table S17. Gene statistics of *D. pinnata*, *C. bipinnatus*, *B. alba* and closely related plant species.

Supplementary Table S18. BUSCO assessment of the gene sets of *D. pinnata*, *C. bipinnatus*, *B. alba* and other related species.

Supplementary Table S19. Statistics of functional annotation for the gene sets based on different databases.

Supplementary Table S20. Statistics of annotated transcription factors.

Supplementary Table S21. The summary of annotated tRNAs.

Supplementary Table S22. The summary of annotated rRNAs and other ncRNAs.

Supplementary Table S23. Statistics of annotated transposable elements.

Supplementary Table S24. Statistics of closely related genomes.

Supplementary Table S25. Statistics of orthogroups in different plants defined by OrthoFinder.

Supplementary Table S26. Gene ID of inulin metabolism genes and transcription factors.

Supplementary Table S27. The Pfam domain annotation of 1-FEH1 and 1-FEH2.

Supplementary Figure S1. Karyotype of *B. alba* by fluorescence in situ hybridization (FISH) technology.

Supplementary Figure S2. Distribution of K-mer (K = 19) frequency in sequencing reads of the three Coreopsideae plants.

Supplementary Figure S3. Hi-C heatmap of chromosomes for *D. pinnata* (A), *C. bipinnatus* (B) and *B. alba* (C).

Supplementary Figure S4. Comparison of gene characteristics among closely related Asteraceae genomes.

Supplementary Figure S5. The estimated insertion time of different types of long terminal repeats (LTR) in *C. bipinnatus* (A) and *B. alba* (B).

Supplementary Figure S6. Species tree built by OrthoFinder.

Supplementary Figure S7. Dot plot of chromosomes alignment for *D. pinnata*.

Supplementary Figure S8. Subgenome (SG) phasing of the *D. pinnata* genome by SubPhaser.

Supplementary Figure S9. Dot plot of chromosomes alignment for *B. alba*.

Supplementary Figure S10. Subgenome (SG) phasing of the *B. alba* genome by SubPhaser.

Supplementary Figure S11. Gene tree constructed of inulin genes and transcription factor genes.

Supplementary Figure S12. Heatmap of inulin metabolism genes and transcription factor genes in *D. pinnata*.

Supplementary Figure S13. Heatmap of inulin metabolism genes and transcription factor genes in *D. pinnata*, *C. bipinnatus* and *B. alba*.

Supplementary Figure S14. Multiple sequence alignment of 1-FFT genes (*g142078.t1* and *g141421.t1*) and pseudogenes (*g31433.t1*, *g31432.t1*, *g87332.t1*, *g87331.t1* and *g87333.t1*) of *B. alba*.

Supplementary Figure S15. Dot plot of synteny gene blocks between A1_10 and A1_11, B1_11 in *B. alba*.**Supplementary Figure S16.** Multiple sequence alignment of 1-FEH1 for closely related species.

Supplementary Figure S17. Multiple sequence alignment of 1-FEH2 for closely related species.

Supplementary Figure S18. Multiple sequence alignment of 1-FFT genes of *D. pinnata*, *C. bipinnatus* and *B. alba*.

Acknowledgments

This work was supported by the National Key R&D Program of China (2021YFC2600101), the Agricultural Science and Technology Innovation Program and the Elite Young Scientists Program of CAAS, and Key Laboratory of Shenzhen (ZDSYS20141118170111640). We thank Yazhi Qin for his assistance in cultivating the plants.

Authors' contributions

H.W. and D.X. prepared plant materials for sequencing, performed genome assembly, gene annotation, evolution, analyses of inulin metabolism and regulation genes; F.J., S.W., A.W., H.L., L.L. and W.Q. provided constructive suggestions. H.W. wrote the draft manuscript. W.F. supervised the project and revised the manuscript.

Competing interests

The authors declare no competing interests.

References

1. Bates D: The growing world of Dahlias. http://www.dahliaworld.co.uk/. 2022.
2. Costa PA, Souza DCd, Ossani PC, Mendes MHA, Silva MLdS, Carvalho EEN, et al. Nutritional and functional compounds in dahlia flowers and roots. Brazilian Journal of Food Technology. 2022;25.
3. Skutnik E, Jędrzejuk A, Rabiza-Świder J, Rochala-Wojciechowska J, Latkowska M and Łukaszewska A. Nanosilver as a novel biocide for control of senescence in garden cosmos. Scientific Reports. 2020;10 1:10274. doi:10.1038/s41598-020-67098-z.
4. IASC: the Invasive Alien Species of China. https://www.iplant.cn/ias/. 2022.
5. Saleem M, Ali HA, Akhtar MF, Saleem U, Saleem A and Irshad I. Chemical characterisation and hepatoprotective potential of Cosmos sulphureus Cav. and Cosmos bipinnatus Cav. Natural Product Research. 2019;33 6:897-900. doi:10.1080/14786419.2017.1413557.
6. Jang I-C, Park J-H, Park E, Park H-R and Lee S-C. Antioxidative and Antigenotoxic Activity of Extracts from Cosmos (Cosmos bipinnatus) Flowers. Plant Foods for Human Nutrition. 2008;63 4:205-10. doi:10.1007/s11130-008-0086-8.
7. Sohn S-H, Yun B-S, Kim S-Y, Choi W-S, Jeon H-S, Yoo J-S, et al. Anti-inflammatory activity of the active components from the roots of Cosmos bipinnatus in lipopolysaccharide-stimulated RAW 264.7 macrophages. Natural Product Research. 2013;27 11:1037-40. doi:10.1080/14786419.2012.686906.
8. Bartolome AP, Villaseñor IM and Yang W-C. Bidens pilosa L. (Asteraceae): Botanical Properties, Traditional Uses, Phytochemistry, and Pharmacology. Evidence-Based Complementary and Alternative Medicine. 2013;2013:340215. doi:10.1155/2013/340215.
9. Mandel JR, Dikow RB, Siniscalchi CM, Thapa R, Watson LE and Funk VA. A fully resolved backbone phylogeny reveals numerous dispersals and explosive diversifications throughout the history of Asteraceae. Proceedings of the National Academy of Sciences. 2019;116 28:14083-8. doi:doi:10.1073/pnas.1903871116.
10. Mort ME, Randle CP, Kimball RT, Tadesse M and Crawford DJ. Phylogeny of Coreopsideae (Asteraceae) inferred from nuclear and plastid DNA sequences. TAXON. 2008;57 1:109-20. http://www.jstor.org/stable/25065952. Accessed 20 May 2024.
11. Crawford DJ and Stuessy TF. The Taxonomic Significance of Anthochlors in the Subtribe Coreopsidinae (Compositae, Heliantheae). American Journal of Botany. 1981;68 1:107-17. doi:https://doi.org/10.1002/j.1537-2197.1981.tb06362.x.
12. Teferra TF. Possible actions of inulin as prebiotic polysaccharide: A review. Food Frontiers. 2021;2 4:407-16. doi:https://doi.org/10.1002/fft2.92.
13. Dwivedi S, Sahrawat K, Puppala N and Ortiz R. Plant prebiotics and human health: Biotechnology to breed prebiotic-rich nutritious food crops. Electronic

Journal of Biotechnology. 2014;17 5:238-45. doi:https://doi.org/10.1016/j.ejbt.2014.07.004.

14. Gupta N, Jangid AK, Pooja D and Kulhari H. Inulin: A novel and stretchy polysaccharide tool for biomedical and nutritional applications. International Journal of Biological Macromolecules. 2019;132:852-63. doi:https://doi.org/10.1016/j.ijbiomac.2019.03.188.

15. Fan W, Wang S, Wang H, Wang A, Jiang F, Liu H, et al. The genomes of chicory, endive, great burdock and yacon provide insights into Asteraceae palaeo-polyploidization history and plant inulin production. Molecular Ecology Resources. 2022;22 8:3124-40. doi:https://doi.org/10.1111/1755-0998.13675.

16. Shen F, He H, Huang X, Deng Y and Yang XZ. Insights into the convergent evolution of fructan biosynthesis in angiosperms from the highly characteristic chicory genome. New Phytologist. 2023;238 3:1245-62. doi:10.1111/nph.18796.

17. Valluru R and Van den Ende W. Plant fructans in stress environments: emerging concepts and future prospects. Journal of Experimental Botany. 2008;59 11:2905-16. doi:10.1093/jxb/ern164.

18. Wang S, Wang A, Chen R, Xu D, Wang H, Jiang F, et al. Haplotype-resolved chromosome-level genome of hexaploid Jerusalem artichoke provides insights into its origin, evolution, and inulin metabolism. Plant Communications. 2023:100767. doi:https://doi.org/10.1016/j.xplc.2023.100767.

19. Zhu Z, He J, Liu G, Barba FJ, Koubaa M, Ding L, et al. Recent insights for the green recovery of inulin from plant food materials using non-conventional extraction technologies: A review. Innovative Food Science & Emerging Technologies. 2016;33:1-9. doi:https://doi.org/10.1016/j.ifset.2015.12.023.

20. Badouin H, Gouzy J, Grassa CJ, Murat F, Staton SE, Cottret L, et al. The sunflower genome provides insights into oil metabolism, flowering and Asterid evolution. Nature. 2017;546 7656:148-+. doi:10.1038/nature22380.

21. Battlay P, Wilson J, Bieker VC, Lee C, Prapas D, Petersen B, et al. Large haploblocks underlie rapid adaptation in the invasive weed Ambrosia artemisiifolia. Nat Commun. 2023;14 1:1717. doi:10.1038/s41467-023-37303-4.

22. Xu XY, Yuan HY, Yu XQ, Huang SZ, Sun YM, Zhang T, et al. The chromosome-level Stevia genome provides insights into steviol glycoside biosynthesis. Hortic Res-England. 2021;8 1 doi:10.1038/s41438-021-00565-4.

23. Liu B, Yan J, Li W, Yin L, Li P, Yu H, et al. Mikania micrantha genome provides insights into the molecular mechanism of rapid growth. Nature Communications. 2020;11 1:340. doi:10.1038/s41467-019-13926-4.

24. Duan S-D, Zheng K-Y, Liu Y, Li X-R, Xiang D-Y, Hao L-H, et al. The complete chloroplast genome and phylogenetic analysis of Dahlia pinnata Cavanilles 1791(Asteraceae: Dahlia). 2023.

25. Jiang M, Chen H, Wang L, You J and Liu C. The complete chloroplast genome sequence of Cosmos bipinnatus, the first of the genus Cosmos. Mitochondrial DNA Part B. 2019;4 2:4204-6. doi:10.1080/23802359.2019.1693298.

26. Huang YL and Kao WY. Chromosome numbers of populations of three varieties of Bidens pilosa in Taiwan. Bot Stud. 2015;56 1:23. doi:10.1186/s40529-015-0107-5.
27. Wu X, Jiang M, Liu M, Wang B, Yu Q, Chen H, et al. Analysis of the complete plastomes of Bidens pilosa L. 1753 (Asteraceae, Coreopsideae) from Beijing, China reveals high genetic diversity and possible misidentifications. Mitochondrial DNA Part B. 2023;8 5:612-8. doi:10.1080/23802359.2023.2189979.
28. Zhang D, Tu J, Ding X, Guan W, Gong L, Qiu X, et al. Analysis of the chloroplast genome and phylogenetic evolution of Bidens pilosa. BMC Genomics. 2023;24 1:113. doi:10.1186/s12864-023-09195-7.
29. Bellinger MR, Datlof EM, Selph KE, Gallaher TJ and Knope ML. A Genome for Bidens hawaiensis: A Member of a Hexaploid Hawaiian Plant Adaptive Radiation. Journal of Heredity. 2022;113 2:205-14. doi:10.1093/jhered/esab077.
30. Veselý P, Bureš P and Šmarda P. Nutrient reserves may allow for genome size increase: evidence from comparison of geophytes and their sister non-geophytic relatives. Ann Bot. 2013;112 6:1193-200. doi:10.1093/aob/mct185.
31. Schie S, Chaudhary R and Debener T. Analysis of a Complex Polyploid Plant Genome using Molecular Markers: Strong Evidence for Segmental Allooctoploidy in Garden Dahlias. The Plant Genome. 2014;7 3:plantgenome2014.01.0002. doi:https://doi.org/10.3835/plantgenome2014.01.0002.
32. Hansen HV and Hjerting JP. Observations on chromosome numbers and biosystematics in Dahlia (Asteraceae, Heliantheae) with an account on the identity of D. pinnata, D. rosea, and D. coccinea. Nordic Journal of Botany. 1996;16 4:445-55. doi:https://doi.org/10.1111/j.1756-1051.1996.tb00256.x.
33. Ohri D, Kumar A and Pal M. Variation in Nuclear DNA and Karyotype in Cosmos. CYTOLOGIA. 1988;53 2:365-7. doi:10.1508/cytologia.53.365.
34. Tereza G-GM, Mansanares ME, Semir J and Solferini VN. Chromosomal studies of three species of Bidens (L.) (Asteraceae). Caryologia. 2006;59 1:14-8. doi:10.1080/00087114.2006.10797892.
35. Vallès J, Maqbool S, Gomez M and Siljak-Yakovlev S. Contribution to knowledge about nuclear DNA amounts in the family Asteraceae: First assessments in one genus and 12 species, with chromosome counts for three taxa. Botanica Serbica. 2017;41:213-9. doi:10.5281/zenodo.1026505.
36. Cheng HY, Jarvis ED, Fedrigo O, Koepfli KP, Urban L, Gemmell NJ, et al. Haplotype-resolved assembly of diploid genomes without parental data. Nat Biotechnol. 2022;40 9:1332-+. doi:10.1038/s41587-022-01261-x.
37. Zhou C, McCarthy SA and Durbin R. YaHS: yet another Hi-C scaffolding tool. Bioinformatics. 2023;39 1 doi:10.1093/bioinformatics/btac808.
38. Durand NC, Robinson JT, Shamim MS, Machol I, Mesirov JP, Lander ES, et al. Juicebox Provides a Visualization System for Hi-C Contact Maps with Unlimited Zoom. Cell Syst. 2016;3 1:99-101. doi:10.1016/j.cels.2015.07.012.

39. Hoff KJ and Stanke M. Predicting Genes in Single Genomes with AUGUSTUS. Curr Protoc Bioinformatics. 2019;65 1:e57. doi:10.1002/cpbi.57.
40. Tian F, Yang DC, Meng YQ, Jin J and Gao G. PlantRegMap: charting functional regulatory maps in plants. Nucleic Acids Res. 2020;48 D1:D1104-D13. doi:10.1093/nar/gkz1020.
41. Song C, Liu Y, Song A, Dong G, Zhao H, Sun W, et al. The Chrysanthemum nankingense Genome Provides Insights into the Evolution and Diversification of Chrysanthemum Flowers and Medicinal Traits. Molecular Plant. 2018;11 12:1482-91. doi:https://doi.org/10.1016/j.molp.2018.10.003.
42. Shen Q, Zhang L, Liao Z, Wang S, Yan T, Shi P, et al. The Genome of Artemisia annua Provides Insight into the Evolution of Asteraceae Family and Artemisinin Biosynthesis. Molecular Plant. 2018;11 6:776-88. doi:https://doi.org/10.1016/j.molp.2018.03.015.
43. Laforest M, Martin SL, Bisaillon K, Soufiane B, Meloche S and Page E. A chromosome-scale draft sequence of the Canada fleabane genome. Pest Management Science. 2020;76 6:2158-69. doi:https://doi.org/10.1002/ps.5753.
44. Emms DM and Kelly S. OrthoFinder: phylogenetic orthology inference for comparative genomics. Genome Biol. 2019;20 1:238. doi:10.1186/s13059-019-1832-y.
45. Zhang C, Huang C-H, Liu M, Hu Y, Panero JL, Luebert F, et al. Phylotranscriptomic insights into Asteraceae diversity, polyploidy, and morphological innovation. Journal of Integrative Plant Biology. 2021;63 7:1273-93. doi:https://doi.org/10.1111/jipb.13078.
46. Kimball RT and Crawford DJ. Phylogeny of Coreopsideae (Asteraceae) using ITS sequences suggests lability in reproductive characters. Molecular Phylogenetics and Evolution. 2004;33 1:127-39. doi:https://doi.org/10.1016/j.ympev.2004.04.022.
47. Bate-Smith EC, Swain T and NÖRdstrom CG. Chemistry and Inheritance of Flower Colour in the Dahlia. Nature. 1955;176 4491:1016-8. doi:10.1038/1761016a0.
48. Gatt M, Ding H, Hammett K and Murray B. Polyploidy and Evolution in Wild and Cultivated Dahlia Species. Annals of Botany. 1998;81 5:647-56. doi:10.1006/anbo.1998.0614.
49. Sorensen PD. Revision of the Genus Dahlia (Compositae, Heliantheae — Coreopsidinae). Rhodora. 1969;71 786:309-65.
50. Van den Ende W, De Coninck B and Van Laere A. Plant fructan exohydrolases: a role in signaling and defense? Trends in Plant Science. 2004;9 11:523-8. doi:https://doi.org/10.1016/j.tplants.2004.09.008.
51. Wei H, Zhao H, Su T, Bausewein A, Greiner S, Harms K, et al. Chicory R2R3-MYB transcription factors CiMYB5 and CiMYB3 regulate fructan 1-exohydrolase expression in response to abiotic stress and hormonal cues. J Exp Bot. 2017;68 15:4323-38. doi:10.1093/jxb/erx210.
52. Wei H, Bausewein A, Greiner S, Dauchot N, Harms K and Rausch T. CiMYB17, a stress-induced chicory R2R3-MYB transcription factor, activates

promoters of genes involved in fructan synthesis and degradation. New Phytol. 2017;215 1:281-98. doi:10.1111/nph.14563.

53. Medema MH, de Rond T and Moore BS. Mining genomes to illuminate the specialized chemistry of life. Nature Reviews Genetics. 2021;22 9:553-71. doi:10.1038/s41576-021-00363-7.

54. De Sadeleer E, Struyf T, Vergauwen R, Le Roy K and Van den Ende W. 1-FFT amino acids involved in high DP inulin accumulation in Viguiera discolor. Frontiers in Plant Science. 2015;6 doi:10.3389/fpls.2015.00616.

55. Jumper J, Evans R, Pritzel A, Green T, Figurnov M, Ronneberger O, et al. Highly accurate protein structure prediction with AlphaFold. Nature. 2021;596 7873:583-+. doi:10.1038/s41586-021-03819-2.

56. Lammens W, Le Roy K, Yuan S, Vergauwen R, Rabijns A, Van Laere A, et al. Crystal structure of 6-SST/6-SFT from Pachysandra terminalis, a plant fructan biosynthesizing enzyme in complex with its acceptor substrate 6-kestose. The Plant Journal. 2012;70 2:205-19. doi:https://doi.org/10.1111/j.1365-313X.2011.04858.x.

57. Versluys M, Porras-Domínguez JR, De Coninck T, Van Damme EJM and Van den Ende W. A novel chicory fructanase can degrade common microbial fructan product profiles and displays positive cooperativity. Journal of Experimental Botany. 2021;73 5:1602-22. doi:10.1093/jxb/erab488.

58. Bourque G, Burns KH, Gehring M, Gorbunova V, Seluanov A, Hammell M, et al. Ten things you should know about transposable elements. Genome Biology. 2018;19 1:199. doi:10.1186/s13059-018-1577-z.

59. Gatt M, Hammett K and Murray B. Confirmation of Ancient Polyploidy in Dahlia (Asteraceae) Species using Genomic In Situ Hybridization. Annals of Botany. 1999;84 1:39-48. doi:https://doi.org/10.1006/anbo.1999.0893.

60. Lamego FP, Charlson D, Delatorre CA, Burgos NR and Vidal RA. Molecular Basis of Resistance to ALS-Inhibitor Herbicides in Greater Beggarticks. Weed Science. 2009;57 5:474-81. doi:10.1614/WS-09-056.1.

61. Shen F, Qin YJ, Wang R, Huang X, Wang Y, Gao TA, et al. Comparative genomics reveals a unique nitrogen-carbon balance system in Asteraceae. Nature Communications. 2023;14 1 doi:10.1038/s41467-023-40002-9.

62. Casey M, Marchioni I, Lear B, Cort AP, Baldwin A, Rogers HJ, et al. Senescence in dahlia flowers is regulated by a complex interplay between flower age and floret position. Frontiers in Plant Science. 2023;13 doi:10.3389/fpls.2022.1085933.

63. Onozaki T and Fujimoto T. Breeding Long Vase Life by Crossing and Selection for Five Generations in Dahlia (Dahlia variabilis) Cut Flowers, and Selection of Fourth-Generation Line 003-15 with Ultra-Long Vase Life. The Horticulture Journal. 2023;92 3:308-22. doi:10.2503/hortj.QH-049.

64. Fan W: Kmerfreq. https://github.com/fanagislab/kmerfreq. Accessed 2022.

65. Liu B, Shi Y, Yuan J, Hu X, Zhang H, Li N, et al. Estimation of genomic characteristics by analyzing k-mer frequency in de novo genome projects. 2013.

66. Guan D, McCarthy SA, Wood J, Howe K, Wang Y and Durbin R. Identifying and removing haplotypic duplication in primary genome assemblies. Bioinformatics. 2020;36 9:2896-8. doi:10.1093/bioinformatics/btaa025.
67. Li H. Minimap2: pairwise alignment for nucleotide sequences. Bioinformatics. 2018;34 18:3094-100. doi:10.1093/bioinformatics/bty191.
68. Waterhouse RM, Seppey M, Simao FA, Manni M, Ioannidis P, Klioutchnikov G, et al. BUSCO Applications from Quality Assessments to Gene Prediction and Phylogenomics. Mol Biol Evol. 2018;35 3:543-8. doi:10.1093/molbev/msx319.
69. Arima: Arima mapping pipeline. https://github.com/ArimaGenomics/mapping_pipeline. 2022.
70. Benson G. Tandem repeats finder: a program to analyze DNA sequences. Nucleic Acids Res. 1999;27 2:573-80. doi:10.1093/nar/27.2.573.
71. Ou S, Su W, Liao Y, Chougule K, Agda JRA, Hellinga AJ, et al. Benchmarking transposable element annotation methods for creation of a streamlined, comprehensive pipeline. Genome Biol. 2019;20 1:275. doi:10.1186/s13059-019-1905-y.
72. da Cruz MHP, Domingues DS, Saito PTM, Paschoal AR and Bugatti PH. TERL: classification of transposable elements by convolutional neural networks. Brief Bioinform. 2021;22 3 doi:10.1093/bib/bbaa185.
73. Stanke M, Diekhans M, Baertsch R and Haussler D. Using native and syntenically mapped cDNA alignments to improve de novo gene finding. Bioinformatics. 2008;24 5:637-44. doi:10.1093/bioinformatics/btn013.
74. Li H. Protein-to-genome alignment with miniprot. Bioinformatics. 2023;39 1 doi:10.1093/bioinformatics/btad014.
75. Brůna T, Li H, Guhlin J, Honsel D, Herbold S, Stanke M, et al. GALBA: Genome Annotation with Miniprot and AUGUSTUS. BMC Bioinformatics. 2023;24(1):327. doi: 10.1186/s12859-023-05449-z.
76. Jones P, Binns D, Chang HY, Fraser M, Li W, McAnulla C, et al. InterProScan 5: genome-scale protein function classification. Bioinformatics. 2014;30 9:1236-40. doi:10.1093/bioinformatics/btu031.
77. Chan PP, Lin BY, Mak AJ and Lowe TM. tRNAscan-SE 2.0: improved detection and functional classification of transfer RNA genes. Nucleic Acids Res. 2021;49 16:9077-96. doi:10.1093/nar/gkab688.
78. Nawrocki EP and Eddy SR. Infernal 1.1: 100-fold faster RNA homology searches. Bioinformatics. 2013;29 22:2933-5. doi:10.1093/bioinformatics/btt509.
79. Kalvari I, Nawrocki EP, Ontiveros-Palacios N, Argasinska J, Lamkiewicz K, Marz M, et al. Rfam 14: expanded coverage of metagenomic, viral and microRNA families. Nucleic Acids Res. 2021;49 D1:D192-D200. doi:10.1093/nar/gkaa1047.
80. Lagesen K, Hallin P, Rodland EA, Staerfeldt HH, Rognes T and Ussery DW. RNAmmer: consistent and rapid annotation of ribosomal RNA genes. Nucleic Acids Res. 2007;35 9:3100-8. doi:10.1093/nar/gkm160.

81. Emms DM and Kelly S. STAG: Species Tree Inference from All Genes. bioRxiv. 2018:267914. doi:10.1101/267914.
82. Emms DM and Kelly S. STRIDE: Species Tree Root Inference from Gene Duplication Events. Molecular Biology and Evolution. 2017;34 12:3267-78. doi:10.1093/molbev/msx259.
83. Edgar RC. MUSCLE: multiple sequence alignment with high accuracy and high throughput. Nucleic Acids Res. 2004;32 5:1792-7. doi:10.1093/nar/gkh340.
84. Kozlov AM, Darriba D, Flouri T, Morel B and Stamatakis A. RAxML-NG: a fast, scalable and user-friendly tool for maximum likelihood phylogenetic inference. Bioinformatics. 2019;35 21:4453-5. doi:10.1093/bioinformatics/btz305.
85. Kumar S, Stecher G, Peterson D and Tamura K. MEGA-CC: computing core of molecular evolutionary genetics analysis program for automated and iterative data analysis. Bioinformatics. 2012;28 20:2685-6. doi:10.1093/bioinformatics/bts507.
86. Wang YP, Tang HB, DeBarry JD, Tan X, Li JP, Wang XY, et al. MCScanX: a toolkit for detection and evolutionary analysis of gene synteny and collinearity. Nucleic Acids Res. 2012;40 7:14. doi:10.1093/nar/gkr1293.
87. Wang D, Zhang Y, Zhang Z, Zhu J and Yu J. KaKs_Calculator 2.0: a toolkit incorporating gamma-series methods and sliding window strategies. Genomics, proteomics & bioinformatics. 2010;8 1:77-80. doi:10.1016/s1672-0229(10)60008-3.
88. Krzywinski M, Schein J, Birol I, Connors J, Gascoyne R, Horsman D, et al. Circos: An information aesthetic for comparative genomics. Genome Res. 2009;19 9:1639-45. doi:10.1101/gr.092759.109.
89. Tang H: JCVI utility libraries. https://github.com/tanghaibao/jcvi. 2022.
90. Jia K-H, Wang Z-X, Wang L, Li G-Y, Zhang W, Wang X-L, et al. SubPhaser: a robust allopolyploid subgenome phasing method based on subgenome-specific k-mers. New Phytologist. 2022;235 2:801-9. doi:https://doi.org/10.1111/nph.18173.
91. Slater GS and Birney E. Automated generation of heuristics for biological sequence comparison. BMC Bioinformatics. 2005;6:31. doi:10.1186/1471-2105-6-31.
92. Baron M: pyBoxshade. https://github.com/mdbaron42/pyBoxshade. 2022.
93. Wang HC, Fan W. Dataset of the genomic study of three plants in Trib. Coreopsideae. Zenodo. 2022. https://doi.org/10.5281/zenodo.10160015.
94. AGIS website: ftp://ftp.agis.org.cn/~fanwei/Coreopsideae_plants/. Accessed 20 May 2024.
95. Wang HC. Code of the genomic study of three plants in Trib. Coreopsideae.Github. 2024. https://github.com/whc2/code_for_Coreopsideae.
96. Wang HC, Xu D, Jiang F, Wang S, Wang A, Liu HW, et al. Supporting data for "The genomes of Dahlia pinnata, Cosmos bipinnatus and Bidens alba in tribe Coreopsideae provide insights into polyploid evolution and inulin biosynthesis" GigaScience Database. 2024. https://doi.org/10.5524/102526.

Figure legends

Figure 1 Genomic features of *D. pinnata*, *C. bipinnata* and *B. alba*

ABC. Circos plots of three plants in tribe Coreopsideae, including *D. pinnata* (**A**), *C. bipinnata* (**B**) and *B. alba* (**C**). Each circos plot has 4 tracks: a, gene density; b, tandem repeat density; c, transposable element (TE) density; d, GC percentage. Feature density and GC percentage were calculated with a 1-Mb window size. A picture with representative traits is in the center of each circos plot. Note that the reference genomes for each species were shown. *C. bipinnata* is diploid, so the haploid genome was used as reference genome. In contrast, *D. pinnata* and *B. alba* are tetraploids, their haploid genomes containing two subgenomes were used as their reference genomes. **D.** The distribution of HiFi reads coverage on the contigs (> 1 Mb) of tetraploid *D. pinnata* and *B. alba*. Each plot has a major peak, suggesting that almost no homologous contigs were collapsed. **E.** The BUSCO completeness of gene sets for 1 haplotype of *D. pinnata* (Dpin_1hap), 2 haplotypes of *D. pinnata* (Dpin_2haps: A1 and A3), *C. bipinnata* (Cbip), 1 haplotype of *B. alba* (Balb_1hap) and 2 haplotypes of *B. alba* (Balb_2haps: A1 and B1).

Figure 2 Distributions and variations of transposable elements

A. Histogram of different types of transposable elements (TEs) for *D. pinnata*, *C. bipinnata* and *B. alba*. **B.** The estimated insertion time of long terminal repeats (LTRs) in *D. pinnata*. The intact LTRs were annotated by EDTA, and pairwise distances from the LTRs were estimated by APE with the K80 model. The sequence divergence (d) of long terminal repeats of each LTR and the mutation rate of sunflower ($r = 1\times10^{-8}$) were used to estimate the times with the equation $T = d/2r$.

Figure 3 Evolution history and chromosome rearrangements in tribe Coreopsideae

A. Phylogenetic trees showing divergence times (Left) and substitution rates (Right) for the three representative plants in tribe Coreopsideae and closely related species. The

blue horizontal bars indicate 95% confidence intervals of the inner nodes. Circle stands for whole genome triplication (WGT), and stars represent whole genome duplications (WGDs). Shapes with a yellow background are whole genome polyploidization events reported previously, while shapes with a red background are those reported in this study. The clade containing the three plants in the tribe Coreopsideae was highlighted with a green background. **B.** Macro-synteny plot of one haplotype of *D. pinnata*, *C. bipinnatus* and one haplotype of *B. alba*. The chromosomes showing 1:1:1 relationships among all three species were highlighted in green, while the chromosomes showing 1:1 relationships among only two species were highlighted in yellow. The others showing multiple chromosome rearrangements were colored in grey.

Figure 4 Evolutionary analyses of the tetraploid *D. pinnata* genome

A. Macro-synteny of 4 haplotypes in *D. pinnata*. Each haplotype contains 16 chromosomes. **B.** Synonymous substitution rate per site (*Ks*) distribution for 1 haplotype of *D. pinnata* (Dpin_1hap), 2 haplotypes of *D. pinnata* (Dpin_2haps: A1 and A3), *C. bipinnata* (Cbip), *H. annuus* (Hann) and between 1 haplotype of *D. pinnata* and *C. bipinnata* (Dpin_Cbip). Speciation and whole-genome polyploidization events are pointed out by arrows. **C.** Phylogenetic trees for each of the 16 homologous chromosome groups. In total, 9 chromosome groups with ((A1, A2), (A3, A4)) topology were highlighted in green background, while 7 chromosome groups with (((A1, A2), A3), A4) topology were highlighted in yellow background.

Figure 5 Evolutionary analyses of the tetraploid *B. alba* genome

A. Macro-synteny of 4 haplotypes in *B. alba*. Each haplotype contains 12 chromosomes. **B.** Synonymous substitution rate per site (*Ks*) distribution for 1 haplotype of *B. alba* (Balb_1hap), 2 haplotypes of *B. alba* (Balb_2haps: A1 and B1), *C. bipinnata* (Cbip), *H. annuus* (Hann) and between 1 haplotype of *B. alba* and *C. bipinnata* (Balb_Cbip). Speciation and whole-genome polyploidization events are pointed out by arrows. **C.** Phylogenetic trees for each of the 12 homologous chromosome groups. All 12 chromosome groups held ((A1, A2), (B1, B2)) topology and were highlighted in green background.

Figure 6 Genes for inulin metabolism and regulation

A. Copy number for the fructan-active-enzyme (FAZY) genes 1-SST, 1-FFT, 1-FEH1 and 1-FEH2 and transcription factor genes MYB17, MYB3 and MYB5 in *D. pinnata*, *C. bipinnatus* and *B. alba*. **B.** Micro-synteny of 1-FFT and 1-SST genes in *D. pinnata*, *C. bipinnatus* and *B. alba*. The tandem duplicated pseudogenes of 1-FFT of *B. alba* were in red. **C.** Phylogenetic tree of 1-FEH1 genes in *D. pinnata* (Dpin), *C. bipinnatus* (Cbip), *B. alba* (Balb), *A. artemisiifolia* (Aart) and *H. annuus* (Hann). (**D**) Phylogenetic tree of 1-FEH2 genes. **E.** Multiple sequence alignment of catalytic triads for the 1-FFT proteins in *D. pinnata*, *C. bipinnatus*, *B. alba* and *Cichorium intybus* (Up). Three conserved residues involved in the reaction were highlighted in red, and the one residue mutated only in one of two 1-FFT genes from *B. alba* was highlighted in cyan. The ribbon views of the catalytic triads for the protein structures of 1-FFTs of *D. pinnata* (Dpin.g7978, red color) and *C. bipinnatus* (Cbip.g6466, yellow color) (Bottom). The structures were aligned together, and the loss of three residues in *C. bipinnatus* severely impacted its protein structures.

Tables

Table 1 Statistics of genome assembly and annotation

	D. pinnata (4×)	*D. pinnata* (2×)	*C. bipinnatus*	*B. alba* (4×)	*B. alba* (2×)
**Genome assembly**					
**Estimate genome size (Gb)**	7.96	3.98	1.08	3.86	1.93
**Total assembly size (bp)**	7,857,309,314	3,928,656,657	1,015,576,390	3,749,125,044	1,874,562,522
**Number of chromosomes**	64	32	12	48	24
**GC percentage**	37.8	37.8	36.8	36.6	36.6
**Contig N50 (bp)**	29,279,538	29,279,538	52,817,601	15,049,174	15,049,174
**Scaffold N50 (bp)**	118,510,872	118,510,872	79,416,621	75,126,418	75,126,418
**% of sequences anchored to chromosomes**	95.5	95.5	93.7	95.2	95.2
**% of telomeres assembled**	70.3	70.3	58.3	57.3	57.3
**BUSCO completeness assembly**	99.1%	98.8%	97.4%	99.6%	98.4%
**Genome annotation**					
**Length of tandem repeats (bp)**	657,362,351	328,681,176	69,395,159	440,213,780	220,106,890
**Length of TE sequences (bp)**	6,887,204,637	3,443,602,318	816,414,084	2,909,120,353	1,454,560,176
**Number of tRNA genes**	4,717	2,358	1,474	4,998	2,499
**Number of rRNA genes**	51,314	25,657	10,828	21,488	10,744
**Number of protein-coding gene models**	181,915	90,958	46,076	165,431	82,716
**Total CDS size (bp)**	224,643,068	112,321,534	58,778,603	181,196,996	90,598,498
**BUSCO completeness of gene set**	98.9%	97.6%	96.7%	98.4%	95.0%

Note: Two haplotypes of *D. pinnata* (A1 and A3) and *B. alba* (A1 and B1) were assessed by BUSCO.

Figure 1

Click here to access/download;Figure;Fig1.png

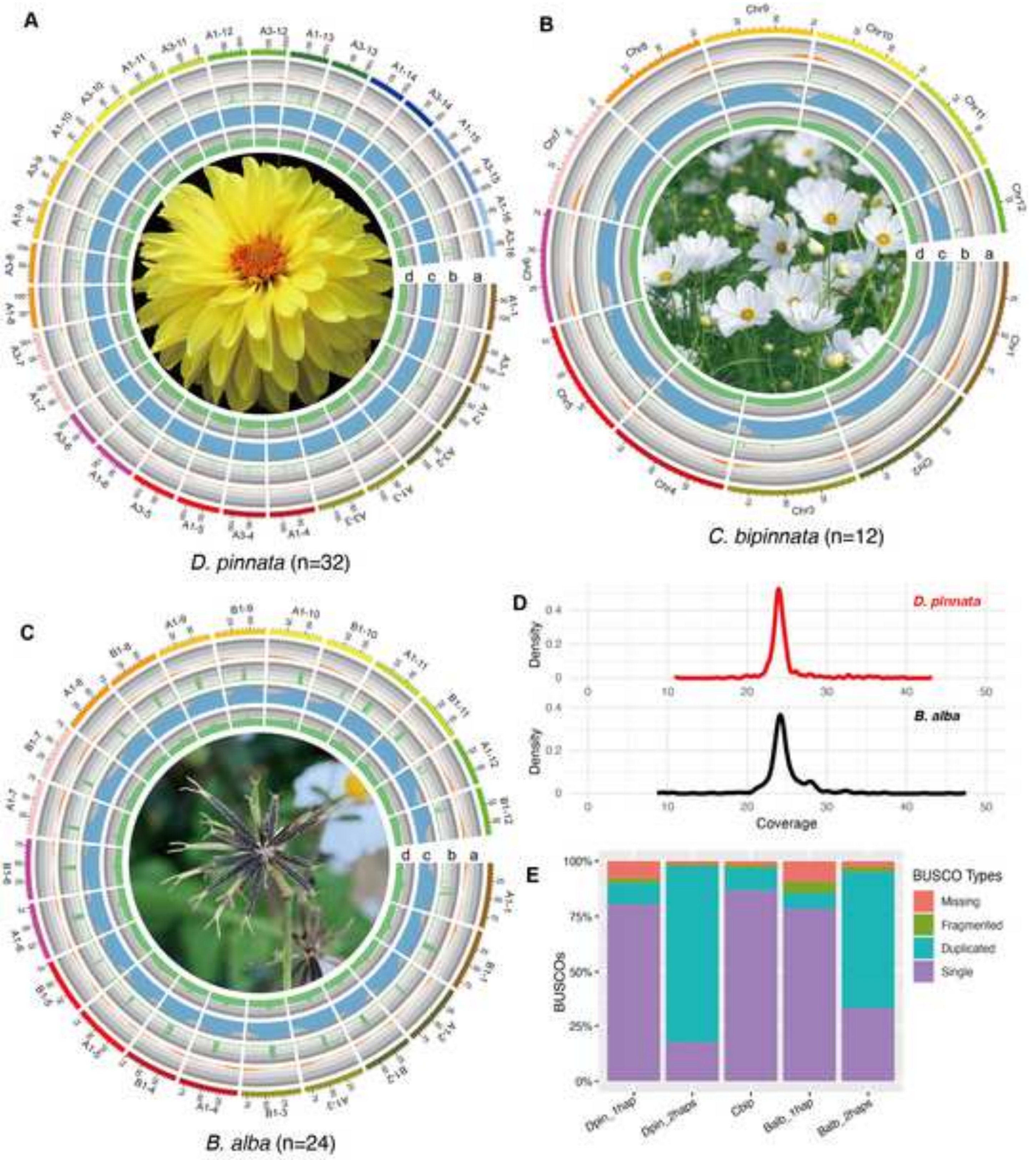

Figure 2

Click here to access/download;Figure;Fig2.png

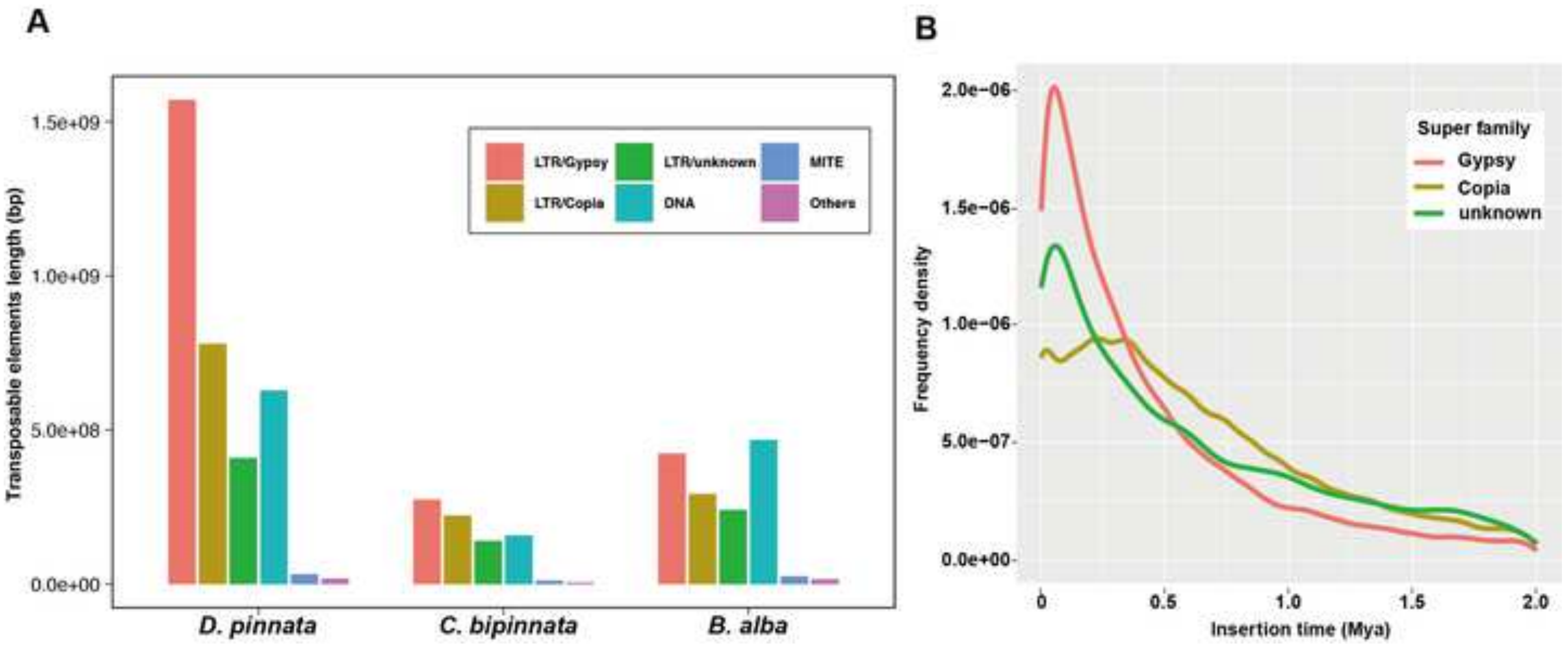

Figure 3

Click here to access/download;Figure;Fig3.png

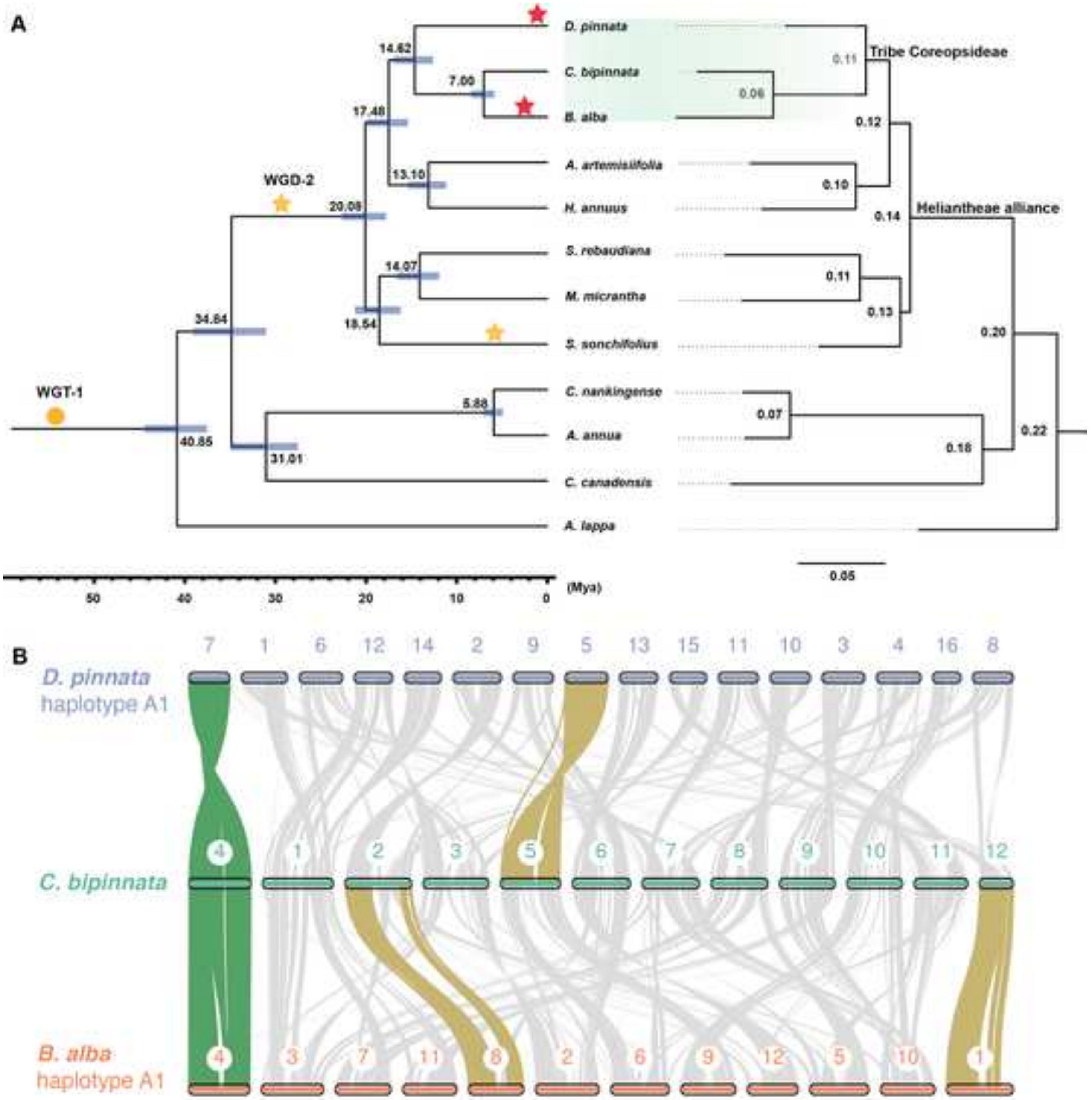

Figure 4 Click here to access/download;Figure;Fig4.png

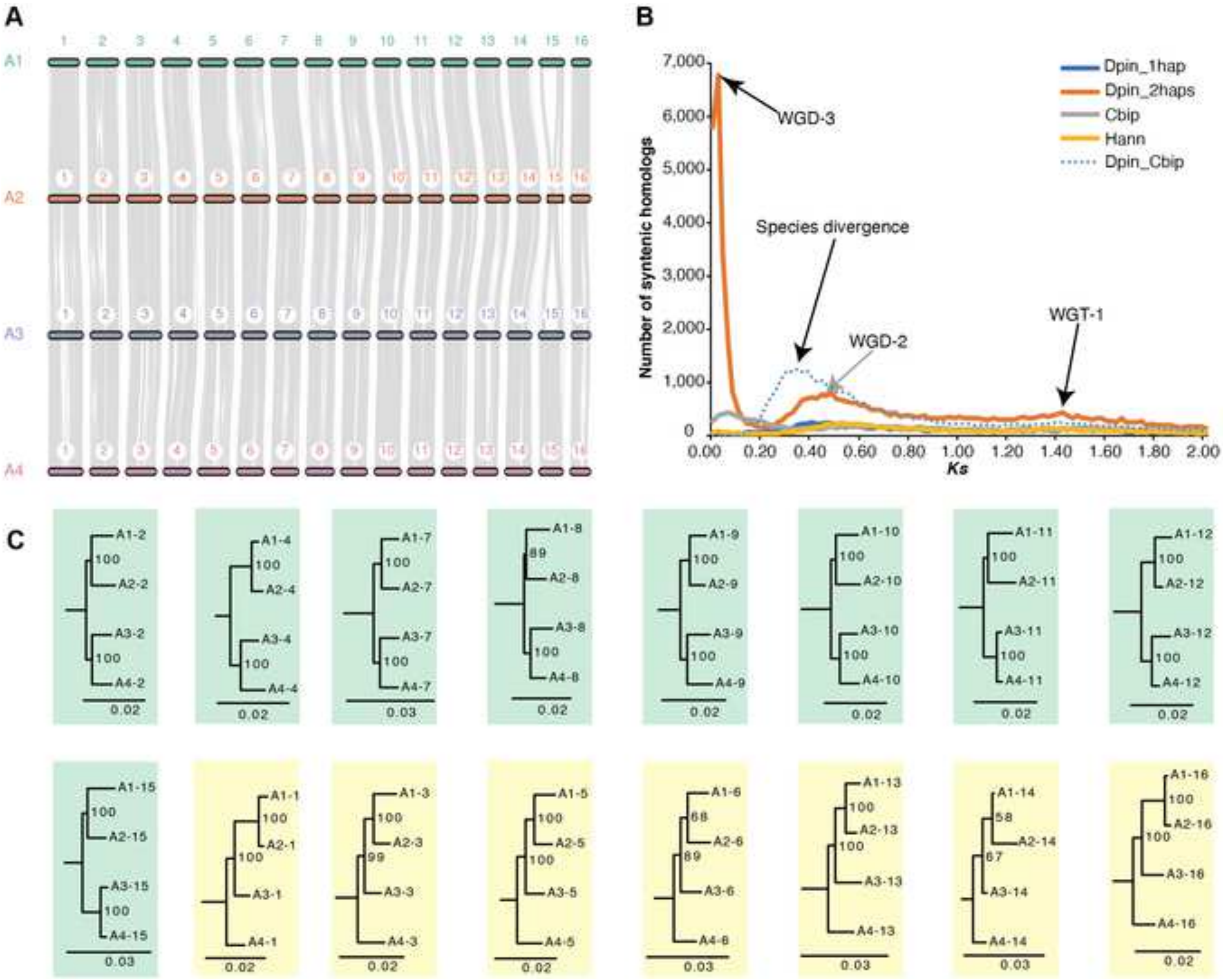

Figure 5

Click here to access/download;Figure;Fig5.png

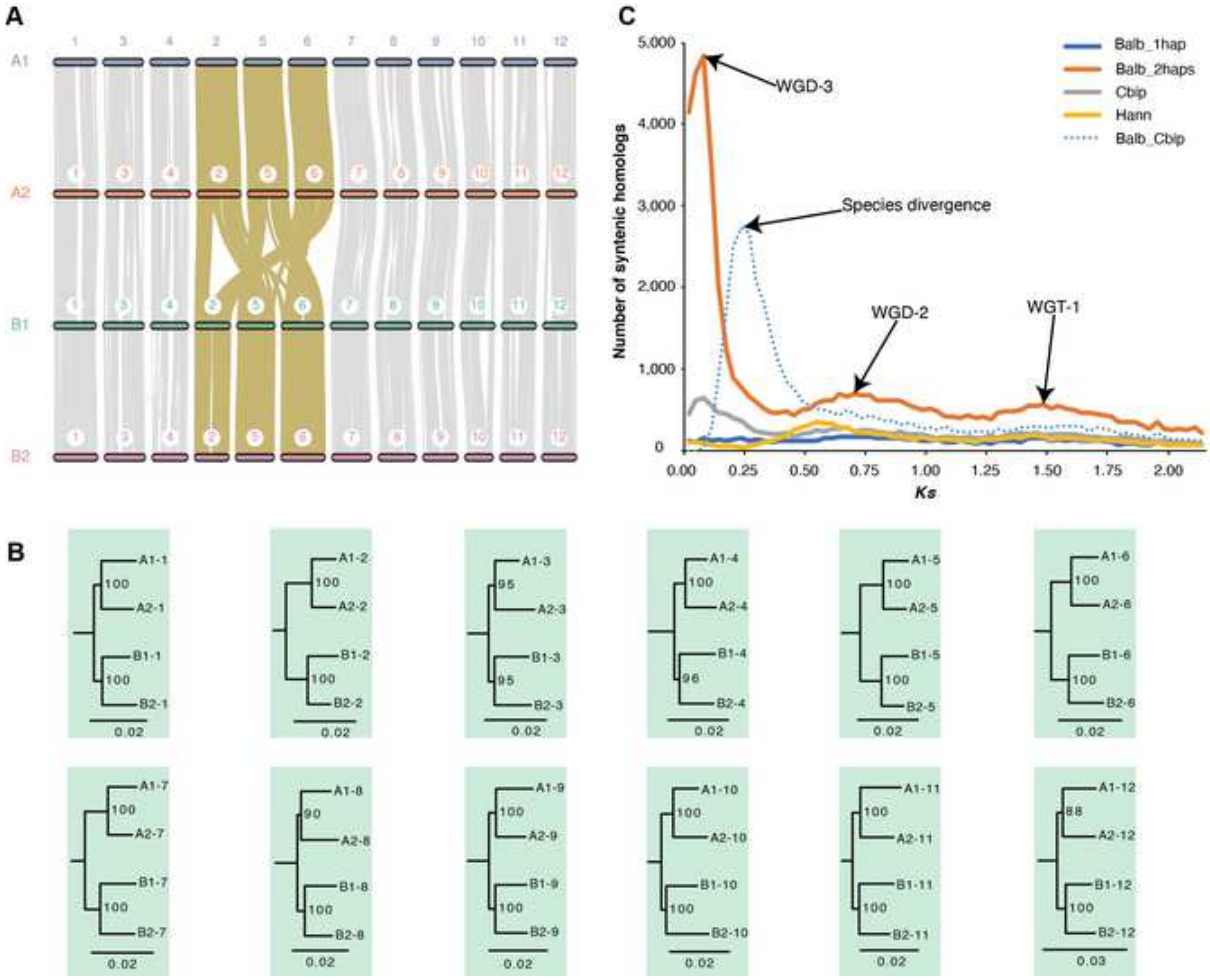

Figure 6

Click here to access/download;Figure;Fig6.png

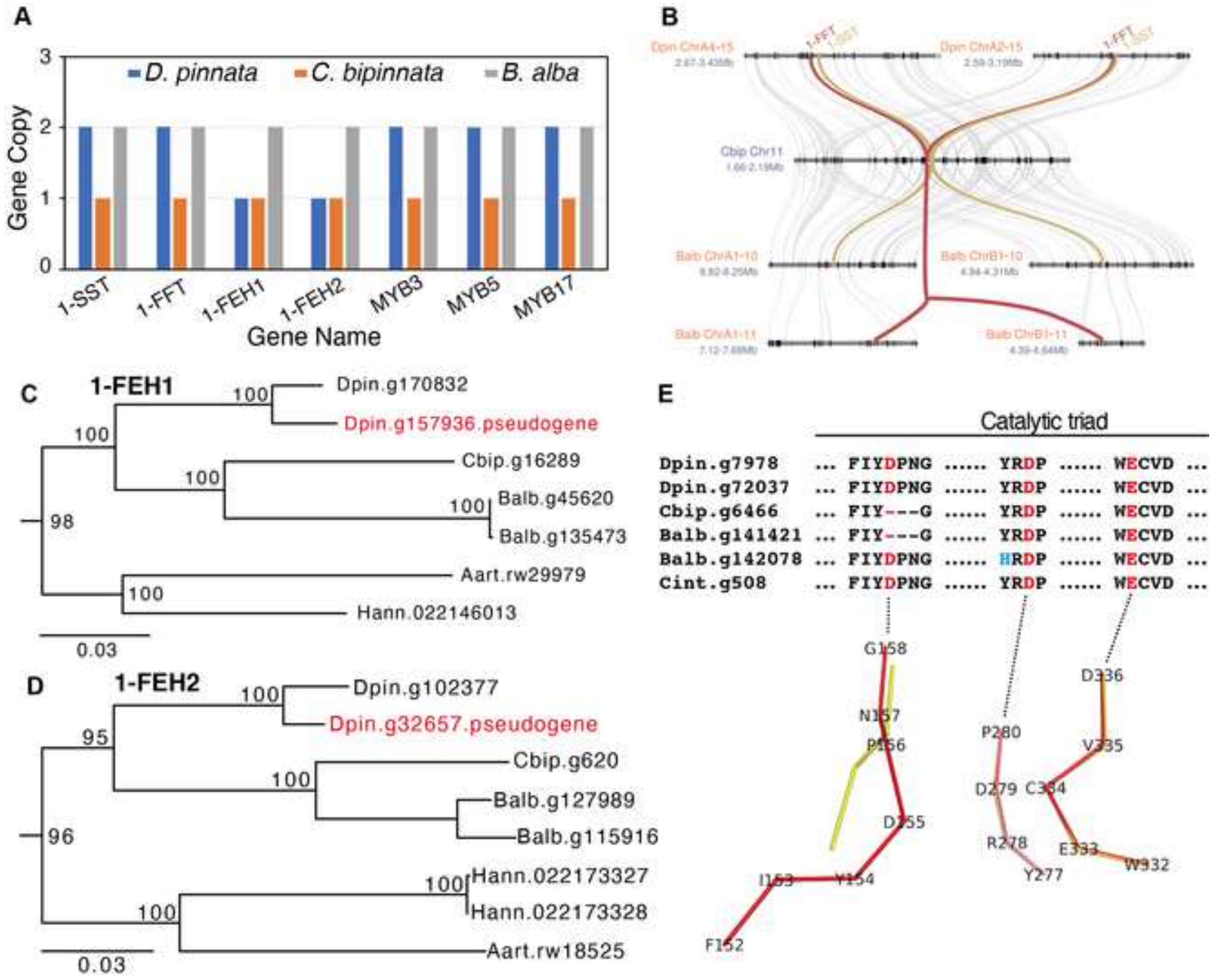

Click here to access/download
Supplementary Material
supp text2.docx

Personal Cover
Click here to access/download;Personal Cover;cover letter2.docx

Dear Editor,

Thank you for the efficient processing of our manuscript, and thanks for your and the reviewers' comments and suggestions to our work. We are delighted to submit the revised manuscript entitled "The genomes of *Dahlia pinnata*, *Cosmos bipinnatus* and *Bidens alba* in tribe Coreopsideae provide insights into polyploid evolution and inulin biosynthesis" to *GigaScience*.

By integrating the PacBio HiFi and Hi-C sequencing reads, we present reference genomes for three plants of the tribe Coreopsideae: *Dahlia pinnata*, *Cosmos bipinnatus* and *Bidens alba*. Using the chromosome-scale haplotype-resolved reference genomes and high-quality gene annotations, we performed phylogenomic analyses and investigated the genes involved in inulin metabolism. The key findings include:

(1) The *Gypsy* transposable elements enlarge the genome of *D. pinnata*.
(2) Multiple chromosome rearrangements have occurred in the tribe Coreopsideae.
(3) *D. pinnata* is an autotetraploid, instead of an octoploid reported by many previous publications.
(4) *B. alba* is an allotetraploid, originated from the recent whole genome duplication (WGD-3) by the hybridization of two ancestor species.
(5) The pseudogenization of exohydrolases of *D. pinnata* and the deletion of 1-FFT key residues of *C. bipinnatus* and *B. alba* may partially explain why *D. pinnata* produces more inulin than the other two plants.

We have revised the manuscript according to you and the reviewers' comments.

"Please include a point-by-point within the 'Response to Reviewers' box in the submission system. Please ensure you describe additional experiments that were carried out and include a detailed rebuttal of any criticisms or requested revisions that you disagreed with. Please also ensure that your revised manuscript conforms to the journal style, which can be found in the Instructions for Authors on the journal homepage. If the data and code has been modified in the revision process please be sure to update the public versions of this too."
Reply: We added scripts used for gene expression calculation based on Isoseq data to GitHub, and updated the readme file.

Thank you for your time in handling our manuscript. If I can provide you with any further information or assistance, please feel free to contact me at fanwei@caas.cn.

Best regards,

Wei Fan, Professor, on behalf of all authors
Agricultural Genomics Institute at Shenzhen
Chinese Academy of Agricultural Sciences
Shenzhen 518120, China
E-mail: fanwei@caas.cn
