# Supplementary material for: The genomes of Dahlia pinnata, Cosmos bipinnatus, and Bidens alba in tribe Coreopsideae provide insights into polyploid evolution and inulin biosynthesis
Source: Gigascience. 2024 Jun 13;13:giae032. doi: 10.1093/gigascience/giae032 (PMC11170221; doi:10.1093/gigascience/giae032)
Supplement: giae032_Response_to_Reviewer_Comments_Original_Submission [file giae032_response_to_reviewer_comments_original_submission.pdf]

# Author's Response To Reviewer Comments

Reviewer reports:

Reviewer #1: Summary
Wang et. al focuses on the assembly, annotation and analysis of three species within the Coreopsideae tribe of the Asteraceae family; Dahlia pinnata, Cosmos bipinnatus, and Bidens alba. With only one genome assembly reported in this tribe previously (Bidens hawaiensis), these genomes represent a big step in understanding the biology of species in this tribe. Using chromosome-level assemblies, the authors find that: D. pinnata is an autotetraploid, B. alba is an allotetraploid, the genera Cosmos and Bidens are more closely related (diverged ~7mya) than either is to Dahlia, and take an initial dive into the biosynthesis of inulin and how it may be differentially regulated across these species. While the data is of high quality and the analysis is thorough, there are a few places where clarification is required as detailed below:

Major Comments
* Double check the Bidens picture in Figure1 as it appears to be Bidens pilosa and not Bidens alba (B. alba has only two awns per achene compared to 3 or more in B. pilosa). Contrary to the text, B. alba should not be considered a variety of B. pilosa (which is hexaploid) and should be referred only as B. alba.
Reply: We have double checked our sample and the picture. The Bidens studied here is B. alba, as confirmed by the karyotype analysis. In the old picture, most achenes have two awns, but some seem have three awns, which may be caused by low resolution. We used a new Bidens picture with high resolution, and removed the description taking B. alba as a variety of B. pilosa.

* It's confusing if data presented are representing haplotypes of a single subgenome or if haplotypes were "forced" within subgenomes to compare across each (for Dahlia and Bidens). While the subgenome discussion comes later, this distinction needs to be made and be clear for data in Figures 1 and S3
Reply: Thank you for this suggestion. We have added the explanation "Note that the reference genomes for each species were shown. C. bipinnata is diploid, so the haploid genome was used as reference genome. In contrast, D. pinnata and B. alba are tetraploids, their haploid genomes containing two subgenomes were used as their reference genomes." in the figure legends of Figures 1, and have added the explanation "As C. bipinnata is diploid, so the haploid genome was used as reference genome. In contrast, D. pinnata and B. alba are tetraploids, the heatmap showed Hi-C signals among all chromosomes." in the figure legends of Figure S3.

* When using Orthofinder to identify orthologs across Asteraceae genomes (for analyses in Figs 3 and S6), were genes pulled from annotations derived from across subgenomes or were a single subgenome represented in these analyses? If gene annotations were used as defined in Lines 139-141, this could greatly reduce power for identifying single-gene orthogroups for phylogenomic comparisons and has the potential to skew the analyses. Could species level comparisons be done for single haplotypes instead?
Reply: For Othofinder analyses, all species used gene annotations from their reference (haploid) genomes. As D. pinnata and B. alba are tetraploid, the gene annotations from both their two subgenomes were included. The orthologous group construction is not afraid of polyploids, because that all the duplicated genes will be assigned into the same orthologous group. To identify the single-copy orthologous group, the diploid species required to have one gene copy, while the tetraploid species (D. pinnata and B. alba) required to have one or two gene copy, and then only one of the two gene copies was kept by random selection. Therefore, our method is an alternative method to that of using a single haplotype for tetraploid species.

Because tetraploids usually lost some duplicated genes through evolution, genes on single haplotype may be incomplete, as shown in Figure 1E. If we use the genes on single haplotype, we may have the risk of losing some orthogroups.

* As subgenomes of either Dahlia or Bidens could not be fully phased using Kmer-based methods, are there other methods that could be explored for this phasing? With data totals from both Hifi and HiC presented, I would expect subgenomes to be resolvable. Especially in the case of an allotetraploid like B. alba. More clarification as to how these methods work (or don't work) would aid in understanding the authors analyses. Additionally, some attempt with at least one other method is encouraged since this is a main take-away from the work.
Reply: HiFi and HiC can only resolve the haplotype assembly, but will not help in dividing subgenomes.

To divide subgenomes of polyploids, current methods mostly utilized the structure difference between the subgenomes. Kmer-based methods depended on the high frequent transposable elements (TEs), which account for most of genomes. However, these methods have a prerequisite: the sequence difference between the two subgenomes should be large enough, i.e. the two ancestors should have experienced different evolution history and accumulated enough differences. Because all the three plants in this study expanded TEs very recently, the difference are not large enough, as showed in Figure 2 and Figure S5, we cannot divide the subgenomes for Dahlia or Bidens by the Kmer-based methods, or any other methods by comparing the structure difference between the subgenomes.

A more effective way to distinguish subgenomes is by sequencing and analyzing their ancestor genomes. However, this is out of ability at current stage. In future, with the availability of more diploid genomes in this tribe Coreopsideae, we will eventually be able to divide the subgenomes by comparing with their ancestor species.

* The inulin biosynthesis pathway is an interesting one to highlight among these genomes! For the pseudogene determination of 1-FEH1 and 1-FEH2 in Dahlia, did you look at more than predicted gene translation output from Orthofinder? What does the gene sequence say in comparison to the truncated predicted translations used for align in Figs. S13-S14? These should be reported, or at least mentioned, in determining that these copies are pseudogenized (and to what extent)
Reply: We aligned the collected public genes to the reference genomes to manually identify any missing genes of the gene sets and to check the gene structures.

We added a new Table S27, showing the Pfam domain annotation of 1-FEH1 and 1-FEH2. Genes of glycosyl hydrolases family 32 contains conservative N-terminal domain and C terminal. However, the N-terminal domain of Dpin.g157936 (1-FEH1) was severely truncated, and the N-terminal domain of Dpin.g32657 was even lost. Thus, these two genes must be pseudogenized.

* In a similar vein, It would be interesting to know if there are any genomic scars in the Bidens FFT and SST loci corresponding to where these genes may have been right next to each other on each chromosome. Since Cosmos and Bidens are more closely related, and even Dahlia shares this organization, I would think there would be some evidence of this at the genomic level looking at gene synteny across these regions.
Reply: Yes, you are right. There are pseudogenized 1-FFT genes nearing 1-SST genes on ChrA1-10 and ChrB1-10 of Bidens. The synteny across these regions were shown in Figure 6B. We now highlighted the pseudogenes in red, and added a Supplemental Figure S14 to indicate that these pseudogenes lost the first of the catalytic triads, which is necessary for their function. Considering the Bidens is an invasive weed and has the fastest mutation rate, it is not strange that the functional genes 1-SST and 1-FFT located on different chromosomes.

Minor Comments
* Lines 66-67 - C3 and C4 mention feels out of place
Reply: We removed this sentence.

* Lines 227-229 - unclear what is meant by "1 haplotype and 2 haplotypes", please clarify
Reply: One haplotype means one subgenome, and two haplotypes mean two subgnomes. In tetraploid

species, there are four haplotypes in the nucleus. We clarified the haplotype in the text, and revised the sentence to “we analyzed the distribution of synonymous substitution rate (Ks) of homologous gene pairs within one haplotype (subgenome) of D. pinnata, two haplotypes of D. pinnata, C. bipinnata, and H. annuus. In comparison, we also analyzed the distribution of Ks between one haplotype of D. pinnata and C. bipinnata.”.

* More introduction to inulin (type of compound, biosynthesis, etc) early on may help bridge the readers understanding of why it's a primary focus of the manuscript
Reply: We added the following sentence to describe the function of inulin in the second paragraph of the Introduction section. “In addition, most plants in the Asteraceae employed inulin as the primary reserve carbohydrates, instead of starches, which may contribute to strong capability of environmental adaptation for these plants [14-17].” References cited are flowing:

14. Fan W, Wang S, Wang H, Wang A, Jiang F, Liu H, et al. The genomes of chicory, endive, great burdock and yacon provide insights into Asteraceae palaeo-polyploidization history and plant inulin production. Molecular Ecology Resources. 2022;22 8:3124-40. doi:https://doi.org/10.1111/1755-0998.13675.
15. Shen F, He H, Huang X, Deng Y and Yang XZ. Insights into the convergent evolution of fructan biosynthesis in angiosperms from the highly characteristic chicory genome. New Phytologist. 2023;238 3:1245-62. doi:10.1111/nph.18796.
16. Valluru R and Van den Ende W. Plant fructans in stress environments: emerging concepts and future prospects. Journal of Experimental Botany. 2008;59 11:2905-16. doi:10.1093/jxb/ern164.
17. Wang S, Wang A, Chen R, Xu D, Wang H, Jiang F, et al. Haplotype-resolved chromosome-level genome of hexaploid Jerusalem artichoke provides insights into its origin, evolution, and inulin metabolism. Plant Communications. 2023:100767. doi:https://doi.org/10.1016/j.xplc.2023.100767.

* Lines 307-308 - Rewrite for clarity. TF regulating these same genes mentioned before?
Reply: We rewrite the sentence “In addition to these functional genes, previous studies also reported some important transcription factors regulating the expression of the key functional genes.” as “In addition to these functional genes, previous studies also reported in chicory, MYB3 and MYB5 can enhance promoter activities of 1-FEH genes in response to abiotic stress and hormonal cues, while MYB17 activates promoters of both synthesis (1-SST and 1-FFT) and degradation (1-FEH) genes under stress.”.

* Line 309 - "decomposing" should be changed to something like "metabolism" or "breakdown"
Reply: Corrected.

* Line 316 and the rest of the manuscript - When referring to a specific gene, it should be italicized and all caps. Ex "1-SST"
Reply: Corrected.

* Figure 3A description calls stars, "pentagrams"
Reply: Corrected.

* Figure S16 is very difficult to interpret, even in light of the amino acid alignments provided. I also feel that it distracts and doesn't add much to overall interpretations. I would encourage authors to consider removing
Reply: We removed Figure S16.

Reviewer #2: The Asteraceae family is the largest angiosperm family, which account for around 10% of all flowering plants, and include many plants employed in medicine, horticulture, ecology, food resources, etc. The authors assembled three chromosome-level genomes of Dahlia pinnata, Cosmos bipinnatus, and

Bidens alba in the tribe Coreopsideae. In terms of a series of analyses, they scanned the WGD events, chromosome rearrangements, the potential reasons of genome expansion. Finally, they identified the genes involved in inulin metabolic pathway, and based on the comparison of fructan systhesis genes, they pointed out the possible reasons why D. pinnata produces much more inulin than the other two plants. Overall, the work is sound, especially the quality of three new assembled genomes, and the manuscript is well prepared. However, I still have some major concerns as follows.

First of all, even the author mentioned the reason why they sequenced these three genomes, I was just wondering the importance of these three species in the whole Asteraceae or what is their new insights for asteraceous species study even for angiosperms.
Reply: In the whole Asteraceae, the tribe Coreopsideae stands for one of the 45 recognized tribes. Coreopsideae belongs to the Heliantheae alliance, which includes several important economic species such as sunflower, Jerusalem artichoke, and Smallanthus sonchifolius (yacon). The sequencing of three species in this study may also benefit the breeding of sunflower, Jerusalem artichoke and yacon, by gene editing technologies with gene resources from closely-related species.

Second, it is well known that inulin is a key innovation trait for Asteraceae. To me, the analyses on the of inulin pathway are far away from sufficiency. So far, there are over 20 genomes of asteraceous species available, especially those species like chicory (which has high level inulin). I suggest a more complete genome comparison work on inulin including more asteraceous species even species from outgroup like Scaevola.
Reply: Thank you for this suggestion. We have added more Asteraceae species to study genes for inulin metabolism and regulation, which can be found on Figure 6CDE and Supplementary Figure S11. We added the sentence “By phylogenetic analysis, we inferred the evolutionary history of the inulin genes in related Asteraceae plants, such as Cichorium intybus and Arctium lappa (Supplemental Fig. S11).” to the section of “Pseudogenization in one of the two copies of 1-FEH1 and 1-FEH2 genes in D. pinnata”.

Scaevola taccada is a coastal shrub. a member of Goodeniaceae that is one of the closest outgroups of Asteraceae. There may be no inulin in Scaevola, as no literature reported inulin from Scaevola. Thus, we do not use it as outgroup. However, we add the sentence “In addition to plants in Coreopsideae, further research could use all available Asteraceae plants and plants in closely related outgroup, such as Scaevola taccada in Goodeniaceae [60], to explore why Asteraceae plants take inulin as the primary reserve carbohydrates.” in the Discussion section and cited the following paper: “60. Shen F, Qin YJ, Wang R, Huang X, Wang Y, Gao TA, et al. Comparative genomics reveals a unique nitrogen-carbon balance system in Asteraceae. Nature Communications. 2023;14 1 doi:10.1038/s41467-023-40002-9.”

In addition, only comparing the genes of fructan systhese genes in these three species is not strong enough to make the conclusion that D. pinnata produces much more inulin than the other two plants. Because this is totally based on the well annotation of these genomes (in fact, gene annotation is full of challenge), cloning these genes and RT-PCR or similar methods which can scan these genes expression are highly recommended.
Reply: The genome assembly and gene annotation has been greatly improved with HiFi reads, full length transcript, and so many homology resources. Thus, we think our gene annotations for inulin metabolism genes are of high quality and can be used for downstream analyses.

We used the well-studied published genes as the query genes to detect homologous genes in our gene sets and genomes, and manually checked the multiple sequences alignment of the reference and the newly found genes. Although there are mutations, these genes aligned from head to tail.

Based on full length transcriptome data, we also calculated the expression of inulin genes for C. bipinnatus and B. alba, and compared them with that of D. pinnata (see Supplementary Figure S13). The two synthesis (1-SST and 1-FFT) genes of D. pinnata expressed more than that of C. bipinnatus and B. alba.

In the end, the main purpose of this project is to finish the genome assembly of these three species and perform genome based evolutionary analyses. By the way, we identified valuable metabolism genes such

as inulin, which can be treated as potential gene resources for future studies. Please forgive that more deeper analyses of these functional genes is out of our interest and ability at current stage.
