# Supplementary material for: The genomes of Dahlia pinnata, Cosmos bipinnatus, and Bidens alba in tribe Coreopsideae provide insights into polyploid evolution and inulin biosynthesis
Source: Gigascience. 2024 Jun 13;13:giae032. doi: 10.1093/gigascience/giae032 (PMC11170221; doi:10.1093/gigascience/giae032)
Supplement: giae032_Reviewer_1_Report_Original_Submission — Daniel Jones -- 2/5/2024 [file giae032_reviewer_1_report_original_submission.pdf]

Reviewer Report

Title: The genomes of Dahlia pinnata, Cosmos bipinnatus and Bidens alba in tribe Coreopsideae provide insights into polyploid evolution and inulin biosynthesis

Version: Original Submission **Date:** 2/5/2024

Reviewer name: Daniel Jones

Reviewer Comments to Author:

Summary
Wang et. al focuses on the assembly, annotation and analysis of three species within the Coreopsideae tribe of the Asteraceae family; Dahlia pinnata, Cosmos bipinnatus, and Bidens alba. With only one genome assembly reported in this tribe previously (Bidens hawaiensis), these genomes represent a big step in understanding the biology of species in this tribe. Using chromosome-level assemblies, the authors find that: D. pinnata is an autotetraploid, B. alba is an allotetraploid, the genera Cosmos and Bidens are more closely related (diverged ~7mya) than either is to Dahlia, and take an initial dive into the biosynthesis of inulin and how it may be differentially regulated across these species. While the data is of high quality and the analysis is thorough, there are a few places where clarification is required as detailed below:

Major Comments

* Double check the Bidens picture in Figure1 as it appears to be Bidens pilosa and not Bidens alba (B. alba has only two awns per achene compared to 3 or more in B. pilosa). Contrary to the text, B. alba should not be considered a variety of B. pilosa (which is hexaploid) and should be referred only as B. alba.
* It's confusing if data presented are representing haplotypes of a single subgenome or if haplotypes were "forced" within subgenomes to compare across each (for Dahlia and Bidens). While the subgenome discussion comes later, this distinction needs to be made and be clear for data in Figures 1 and S3
* When using Orthofinder to identify orthologs across Asteraceae genomes (for analyses in Figs 3 and S6), were genes pulled from annotations derived from across subgenomes or were a single subgenome represented in these analyses? If gene annotations were used as defined in Lines 139-141, this could greatly reduce power for identifying single-gene orthogroups for phylogenomic comparisons and has the potential to skew the analyses. Could species level comparisons be done for single haplotypes instead?
* As subgenomes of either Dahlia or Bidens could not be fully phased using Kmer-based methods, are there other methods that could be explored for this phasing? With data totals from both Hifi and HiC presented, I would expect subgenomes to be resolvable. Especially in the case of an allotetraploid like B. alba. More clarification as to how these methods work (or don't work) would aid in understanding the authors analyses. Additionally, some attempt with at least one other method is encouraged since this is a main take-away from the work.
* The inulin biosynthesis pathway is an interesting one to highlight among these genomes! For the pseudogene determination of 1-FEH1 and 1-FEH2 in Dahlia, did you look at more than predicted gene

translation output from Orthofinder? What does the gene sequence say in comparison to the truncated predicted translations used for align in Figs. S13-S14? These should be reported, or at least mentioned, in determining that these copies are pseudogenized (and to what extent)
* In a similar vein, It would be interesting to know if there are any genomic scars in the Bidens FFT and SST loci corresponding to where these genes may have been right next to each other on each chromosome. Since Cosmos and Bidens are more closely related, and even Dahlia shares this organization, I would think there would be some evidence of this at the genomic level looking at gene synteny across these regions.

Minor Comments
* Lines 66-67 - C3 and C4 mention feels out of place
* Lines 227-229 - unclear what is meant by "1 haplotype and 2 haplotypes", please clarify
* More introduction to inulin (type of compound, biosynthesis, etc) early on may help bridge the readers understanding of why it's a primary focus of the manuscript
* Lines 307-308 - Rewrite for clarity. TF regulating these same genes mentioned before?
* Line 309 - "decomposing" should be changed to something like "metabolism" or "breakdown"
* Line 316 and the rest of the manuscript - When referring to a specific gene, it should be italicized and all caps. Ex "1-SST"
* Figure 3A description calls stars, "pentagrams"
* Figure S16 is very difficult to interpret, even in light of the amino acid alignments provided. I also feel that it distracts and doesn't add much to overall interpretations. I would encourage authors to consider removing

Methods

Are the methods appropriate to the aims of the study, are they well described, and are necessary controls included? Choose an item.

Conclusions

Are the conclusions adequately supported by the data shown? Choose an item.

Reporting Standards

Does the manuscript adhere to the journal's guidelines on minimum standards of reporting? Choose an item.

Choose an item.

Statistics

Are you able to assess all statistics in the manuscript, including the appropriateness of statistical tests used? Choose an item.

Quality of Written English

Please indicate the quality of language in the manuscript: Choose an item.

Declaration of Competing Interests

Please complete a declaration of competing interests, considering the following questions:

- Have you in the past five years received reimbursements, fees, funding, or salary from an organisation that may in any way gain or lose financially from the publication of this manuscript, either now or in the future?
- Do you hold any stocks or shares in an organisation that may in any way gain or lose financially from the publication of this manuscript, either now or in the future?
- Do you hold or are you currently applying for any patents relating to the content of the manuscript?
- Have you received reimbursements, fees, funding, or salary from an organization that holds or has applied for patents relating to the content of the manuscript?
- Do you have any other financial competing interests?
- Do you have any non-financial competing interests in relation to this paper?

If you can answer no to all of the above, write 'I declare that I have no competing interests' below. If your reply is yes to any, please give details below.

I declare I have no competing interests

I agree to the open peer review policy of the journal. I understand that my name will be included on my report to the authors and, if the manuscript is accepted for publication, my named report including any attachments I upload will be posted on the website along with the authors' responses. I agree for my report to be made available under an Open Access Creative Commons CC-BY license (http://creativecommons.org/licenses/by/4.0/). I understand that any comments which I do not wish to be included in my named report can be included as confidential comments to the editors, which will not be published.

Choose an item.

To further support our reviewers, we have joined with Publons, where you can gain additional credit to further highlight your hard work (see: https://publons.com/journal/530/gigascience). On publication of this paper, your review will be automatically added to Publons, you can then choose whether or not to claim your Publons credit. I understand this statement.

Yes Choose an item.
