# Supplementary material for: The genomes of Dahlia pinnata, Cosmos bipinnatus, and Bidens alba in tribe Coreopsideae provide insights into polyploid evolution and inulin biosynthesis
Source: Gigascience. 2024 Jun 13;13:giae032. doi: 10.1093/gigascience/giae032 (PMC11170221; doi:10.1093/gigascience/giae032)
Supplement: giae032_Reviewer_2_Report_Original_Submission — Xiaozeng Yang -- 2/10/2024 [file giae032_reviewer_2_report_original_submission.pdf]

Reviewer Report

Title: The genomes of Dahlia pinnata, Cosmos bipinnatus and Bidens alba in tribe Coreopsideae provide insights into polyploid evolution and inulin biosynthesis

Version: Original Submission **Date:** 2/10/2024

Reviewer name: Xiaozeng Yang

Reviewer Comments to Author:

The Asteraceae family is the largest angiosperm family, which account for around 10% of all flowering plants, and include many plants employed in medicine, horticulture, ecology, food resources, etc. The authors assembled three chromosome-level genomes of Dahlia pinnata, Cosmos bipinnatus, and Bidens alba in the tribe Coreopsideae. In terms of a series of analyses, they scanned the WGD events, chromosome rearrangements, the potential reasons of genome expansion. Finally, they identified the genes involved in inulin metabolic pathway, and based on the comparison of fructan systhesis genes, they pointed out the possible reasons why D. pinnata produces much more inulin than the other two plants. Overall, the work is sound, especially the quality of three new assembled genomes, and the manuscript is well prepared. However, I still have some major concerns as follows.
First of all, even the author mentioned the reason why they sequenced these three genomes, I was just wondering the importance of these three species in the whole Asteraceae or what is their new insights for asteraceous species study even for angiosperms.
Second, it is well known that inulin is a key innovation trait for Asteraceae. To me, the analyses on the of inulin pathway are far away from sufficiency. So far, there are over 20 genomes of asteraceous species available, especially those species like chicory (which has high level inulin). I suggest a more complete genome comparison work on inulin including more asteraceous species even species from outgroup like Scaevola. In addition, only comparing the genes of fructan systhese genes in these three species is not strong enough to make the conclusion that D. pinnata produces much more inulin than the other two plants. Because this is totally based on the well annotation of these genomes (in fact, gene annotation is full of challenge), cloning these genes and RT-PCR or similar methods which can scan these genes expression are highly recommended.

Methods

Are the methods appropriate to the aims of the study, are they well described, and are necessary controls included? Choose an item.

Conclusions

Are the conclusions adequately supported by the data shown? Choose an item.

Reporting Standards

Does the manuscript adhere to the journal's guidelines on minimum standards of reporting? Choose an item.

Choose an item.

Statistics

Are you able to assess all statistics in the manuscript, including the appropriateness of statistical tests used? Choose an item.

Quality of Written English

Please indicate the quality of language in the manuscript: Choose an item.

Declaration of Competing Interests

Please complete a declaration of competing interests, considering the following questions:

- Have you in the past five years received reimbursements, fees, funding, or salary from an organisation that may in any way gain or lose financially from the publication of this manuscript, either now or in the future?
- Do you hold any stocks or shares in an organisation that may in any way gain or lose financially from the publication of this manuscript, either now or in the future?
- Do you hold or are you currently applying for any patents relating to the content of the manuscript?
- Have you received reimbursements, fees, funding, or salary from an organization that holds or has applied for patents relating to the content of the manuscript?
- Do you have any other financial competing interests?
- Do you have any non-financial competing interests in relation to this paper?

If you can answer no to all of the above, write 'I declare that I have no competing interests' below. If your reply is yes to any, please give details below.

I declare that I have no competing interests.

I agree to the open peer review policy of the journal. I understand that my name will be included on my report to the authors and, if the manuscript is accepted for publication, my named report including any attachments I upload will be posted on the website along with the authors' responses. I agree for my report to be made available under an Open Access Creative Commons CC-BY license (http://creativecommons.org/licenses/by/4.0/). I understand that any comments which I do not wish to be included in my named report can be included as confidential comments to the editors, which will not be published.

Choose an item.

To further support our reviewers, we have joined with Publons, where you can gain additional credit to further highlight your hard work (see: https://publons.com/journal/530/gigascience). On publication of this paper, your review will be automatically added to Publons, you can then choose whether or not to claim your Publons credit. I understand this statement.

Yes Choose an item.
